# Supplementary material for: Critical Offset Magnetic PArticle SpectroScopy for rapid and highly sensitive medical point-of-care diagnostics
Source: Nat Commun. 2022 Nov 24;13:7230. doi: 10.1038/s41467-022-34941-y (PMC9700695; doi:10.1038/s41467-022-34941-y)
Supplement: Supplementary file 1 — Supplementary Information [file 41467_2022_34941_MOESM1_ESM.docx]

**Supplementary file: Critical Offset Magnetic Particle SpectroScopy for rapid and highly sensitive medical point-of-care diagnostic**

***Supplementary Note 1: Description of nodes and dips within MNP spectra***

The behavior of the harmonic spectral signal *A*_n_ in dependency of increasing magnetic offset fields *H*_DC_ follows the Chebyshev polynomials closely. The dependency shows more and more nodes (zero-points) for higher harmonic numbers *n* (Fig. 1) **^1^**

| $A_{n}\left( H_{\mathrm{DC}} \right)=-\frac{2i}{T}M^{'}\left( H_{\mathrm{DC}} \right)*\left( U_{n-1}\left( \frac{H_{\mathrm{DC}}}{H_{\mathrm{AC}}} \right)\cdot\sqrt{1-\left( \frac{H_{\mathrm{DC}}}{H_{\mathrm{AC}}} \right)^{2}} \right),$ | (1) |
| --- | --- |

where *U*_n-1_ represents the Chebyshev polynomials of the second kind and *M’(H*_DC_*)* is the derivative of the magnetic response. These ‘dips’ within a Fourier spectrum of a MPS signal can be explained by two separate signals *S*_pos_ and *S*_neg_, which can be generated from the time signal *S*(*t*)=d*M*(*t*)/d*t*=*S*_pos_(*t*)+*S*_neg_(*t*), which can be seen as the induction signal captured by a receive coil following Faradays’ law.


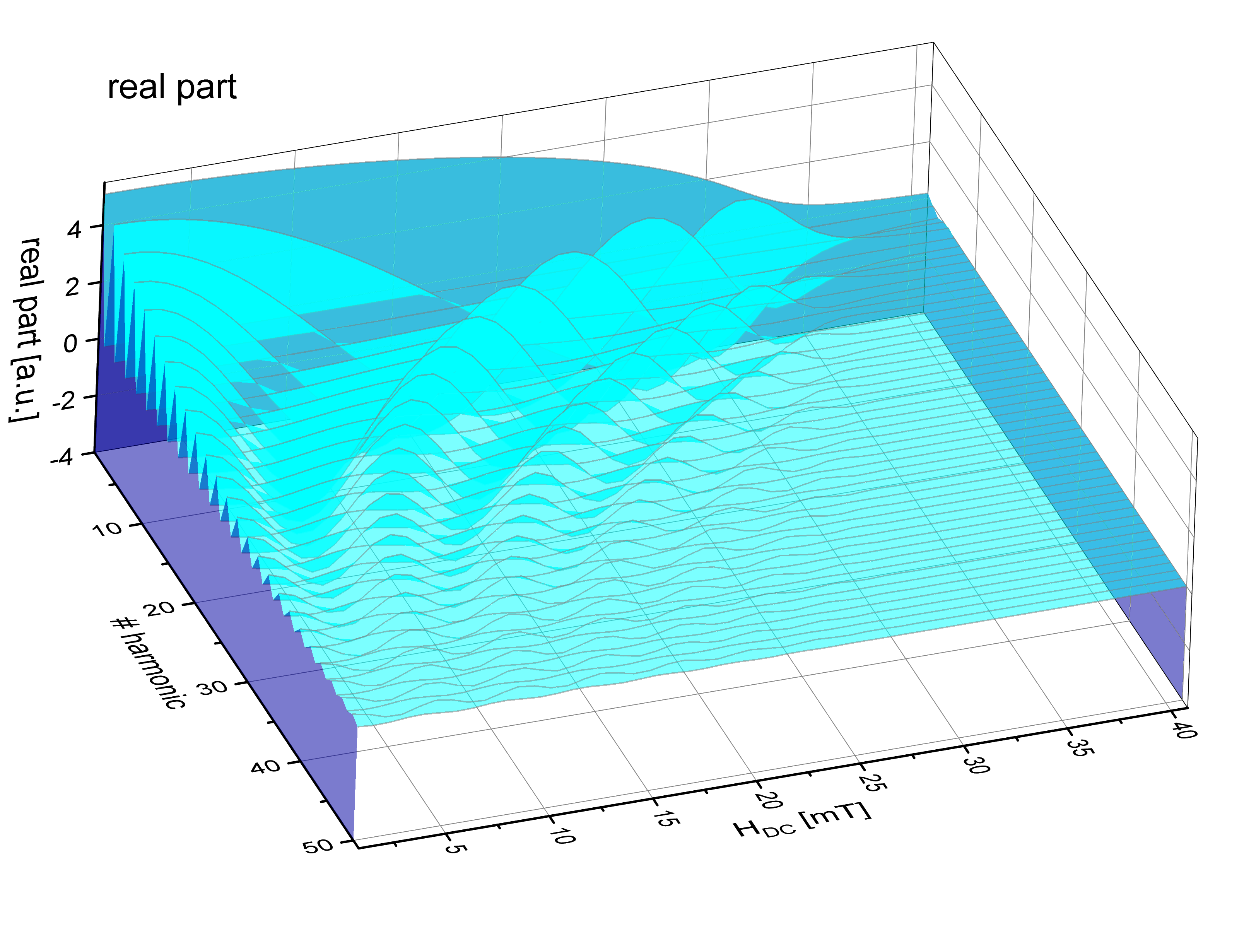


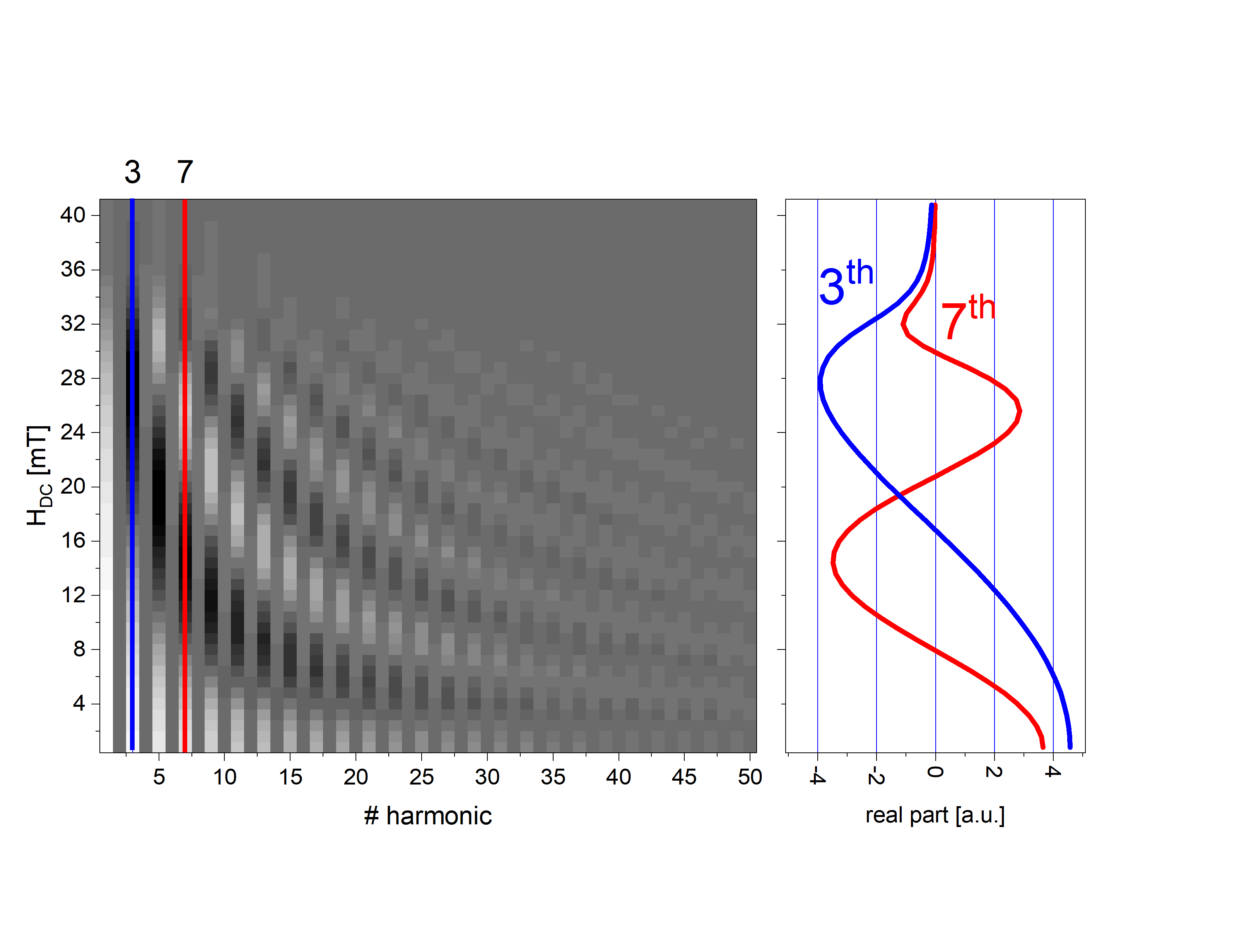


Supplementary figure 1. **Chebyshev polynomial visualization**. **Top** 3D plot of real spectra in dependency of increasing offset magnetic fields *H*_DC_ (*H*_AC_≈40 mT). **Bottom** 2D gray-scaled plots of real part of the spectra. It shows the wave-like shape along the *H*_DC_ axis for the 3^rd^ and 7^th^ harmonic, which follows the Chebyshev polynomials of the second kind convolved with the derivative of the magnetic response.

The signals *S*_pos_ and *S*_neg_ consists of the positive signal peaks and the negative signal peaks of the derivative signal *S* (Fig. 2). The Fourier transforms for both *F*(*S*_pos_) and *F*(*S*_neg_) do not show any ‘dips’ in the absolute spectrum, but the sum of both spectra *F*(*S*_pos_+ *S*_neg_) does. A closer look at a single harmonic *A*_n_ at this specific offset field *H*_DC_ shows destructive interference (both amplitudes almost equal |*A*_n,pos_/*A*_n,neg_|≈1 and a phase difference of almost d*φ*=*φ*_1_-*φ*_2_≈180°):

| $A_{n}\left( H_{\mathrm{DC}}, t \right)=A_{n,neg}\cdot\sin\left( 2\pi\cdot t+\varphi_{n,neg} \right)+A_{n,pos}\cdot\sin\left( 2\pi\cdot t+\varphi_{n,pos}+\Delta\varphi\right).$ | (2) |
| --- | --- |

The phase at such ‘dips’ (Fourier spectrum) or nodes (*A*_n_(*H*_DC_) plots) jumps almost 180° with a steep slope. This implies a high sensitivity to minimal changes, which explains the designation as critical point (CP).


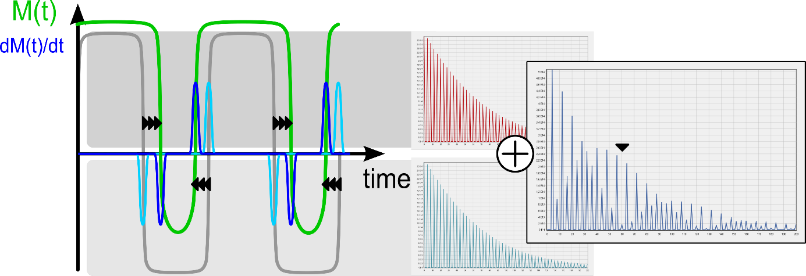


Supplementary figure 2. **Dip-creation by signal interference**. Two magnetization curves over time *M*(*t*) are shown for different offset magnetic fields *H*_DC_. The derivative of both (induction signal) shows positive and negative peaks on the x-axis, which moves to each other due to the asymmetry caused by the offset magnetic field. This asymmetry generates even higher harmonics in the Fourier spectrum. When performing a Fourier transform on either the positive data *S*_pos_ or the negative data *S*_neg_, both spectra consist of even and odd higher harmonics, but without any ‘dips’. In the sum *S*=*S*_pos_+*S*_neg_, the harmonics interfere and produce destructive harmonic signals (dip).

***Supplementary Note 2: Realistic MNPs and simulation model***

Idealized MNP ensembles follow the single particle model and do not show the mentioned phase differences *dφ* (Fig. 3) since they follow the assumption that the particles can instantaneously follow the external magnetic field. They also do not show particle-particle interaction effects.**^2^** In contrast, real particle behavior is described mainly by two types of relaxation effects, Brownian relaxation and Néel relaxation.**^3, 4^** Brownian relaxation is dominant for large particles and describe the rotation of the particle within the surrounding medium, Néel relaxation describes the rotation of the particle magnetization *m* inside the particle, which becomes more dominant for smaller particles. For example, for magnetite particles the transition range between Néel and Brown regime is around 25 nm core diameter.**^5^** Both relaxation types affect the amplitude of the magnetization as well as the phase difference between excitation field and signal, especially in the non-linear case at higher harmonics. The theory for Néel and Brownian relaxation are idealized models, which cannot be directly applied to typical multicore particle systems.**^3^**

The Langevin-function (equation (1) in the manuscript) assumes, that the magnetic moments of the particles follow the external field instantaneously. Given high enough frequencies, this is no longer a valid approximation. In addition, the preferred particles for the given method are nanoparticles with a magnetic moment fixed inside the particle, which maximizes the dependency of the magnetic response on viscosity of the suspending liquid and changes in the particle’s hydrodynamic diameter. It is modelled by the Langevin-equation **^2, 6^**

|  | $\frac{d\mathbf{m}}{dt}=\frac{\mu}{\zeta}\left( \mathbf{m}\times\mathbf{H} \right)\times\mathbf{m}+\sqrt{\frac{2k_{B}T}{\zeta}}\boldsymbol{\lambda}\times\mathbf{m},$ |  | (3) |
| --- | --- | --- | --- |

with *k*_B_ as the Boltzmann constant, *T* as the Temperature, **λ** as normally distributed random vectors with expectation value <**λ**_i_(*t*)>=0 and *ζ*=*κ*·*η*·*R*³ as the Stokes-Einstein diffusion coefficient (viscosity-coefficient) which depends on the viscosity *η* of the surrounding liquid, the hydrodynamic particle diameter *R*, a shape factor *κ* and the magnetic permeability *μ*. In general, the magnetic moment cannot be assumed completely fixed inside the magnetic particles. A first order approximation of a more generalized particle system can be replacing *ζ* with a reduced effective value reflecting the mobility of the magnetization inside the particle, which can only be determined empirically.

Changes in these parameters also change the dependence of the harmonic on *H*_DC_, i.e., it changes the shape of the Chebyshev-like polynomial affecting amplitude, phase and the position of nodes and critical points.

It must be emphasized, that following the theory of Debye **^7^**, which holds true for weak excitation fields, e.g., used in ACS, the characteristic frequency *f*_char_ of MNPs with hydrodynamic diameters of 330 nm can be calculated to about 12 Hz, which is much lower than the frequency used in MPS or COMPASS. But this model applies only for very weak fields. In MPS the rotation rate caused by magnetic moment of the particles and the magnetic fields strengths is much higher than the rotation rate caused by thermal random forces (see equation (3)). This can be described by the critical frequency *f*_crit_ of a particle system. Above this frequency the particles cannot follow an external rotating magnetic field synchronously. In case of a linearly oscillating magnetic field *f*_crit_ can be used as a cut-off frequency estimation for the generation of higher harmonics. This frequency can be calculated according to equation (3) **^8^** to

|  | $f_{\mathrm{crit}}=\frac{m}{2\pi\zeta}\mu H.$ |  | (4) |
| --- | --- | --- | --- |

An exemplary magnetic moment for 25 nm single crystallite magnetite particles (SHP-25, Ocean nanotech, USA) is given as 2·10^-18^ A·m^2^.**^9^** Thus, for 330 nm the upper limit of the magnetic moment can be estimated to be about 2.3·10^-15^ A·m^2^. The actual value will be significantly smaller since those large particles are multi-core particles and it can also be dependent on the applied magnetic field. The assumed value used in the following exemplary simulation is 4·10^-16^ A·m^2^. This gives a critical frequency of *f*_crit_=4·10^6^ T/s·40 mT/2/*π*=25.5 kHz. It is much higher than the characteristic frequency *f*_char_, which one obtains in the diffusion limited small-field range.

To get a better understanding of the COMPASS effect, a basic simulation using Langevin-equation **^2, 5^** (see equation (3)) of two different particle systems with a minimal difference of about 19% in their *ζ*-parameter, which corresponds to, e.g., a small change in the effective hydrodynamic particle diameter of about 6%, were performed. This was done using a simulation framework developed at our department based on (equation (3)) for calculating magnetic fields and non-linear magnetization responses on time-varying magnetic fields **^10^** (simulation parameters can be found in Tab. 1).

Supplementary table 1. **Parameters used for particle signal simulation.** The parameters are oriented on the used APTES-MNP particle system.

| parameter | value |
| --- | --- |
| magnetic moment | *m*=4·10^-16^ A·m^2^ |
| viscosity | *η*=1 mPa·s |
| *ζ*-parameter | *ζ*= *κ*·*η*·*R*^3^ [J·s] |
| shape factor | *κ*=3 (spherical) |
| hydrodynamic diameter | *R*=330 nm |
| “quality factor” | *m*/*ζ*=4·10^6^ T/s |
| Brownian relaxation time | *𝜏*_B_=*ζ*/(*k*_B_*T*) [s] |
| Magnitude of stochastic term in (3) | √(2*k*_B_*T*/*ζ*)·\|**λ**\|  =√(2*k*_B_*T*/*ζ*)·1/√Δ*t*=1·10^-4^ s^-1^ |

At a specific excitation field strength *H*_AC_ and frequency *f*_1_ of the excitation signal, multiple data sets are generated at different offset magnetic fields *H*_DC_. A full set of Chebyshev-like polynomials (1^st^ to 11^th^ harmonic) is generated for both MNP sample variants.


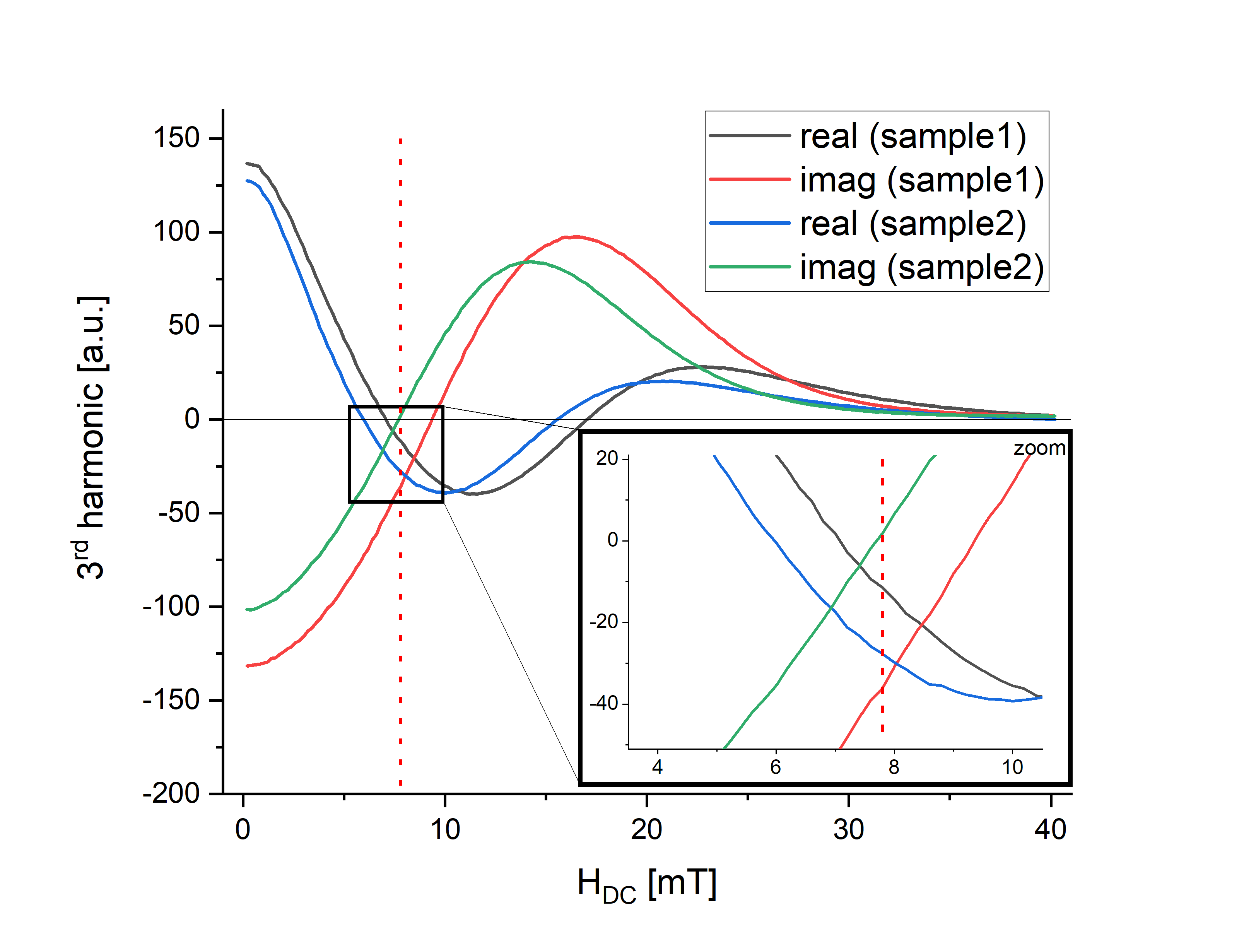


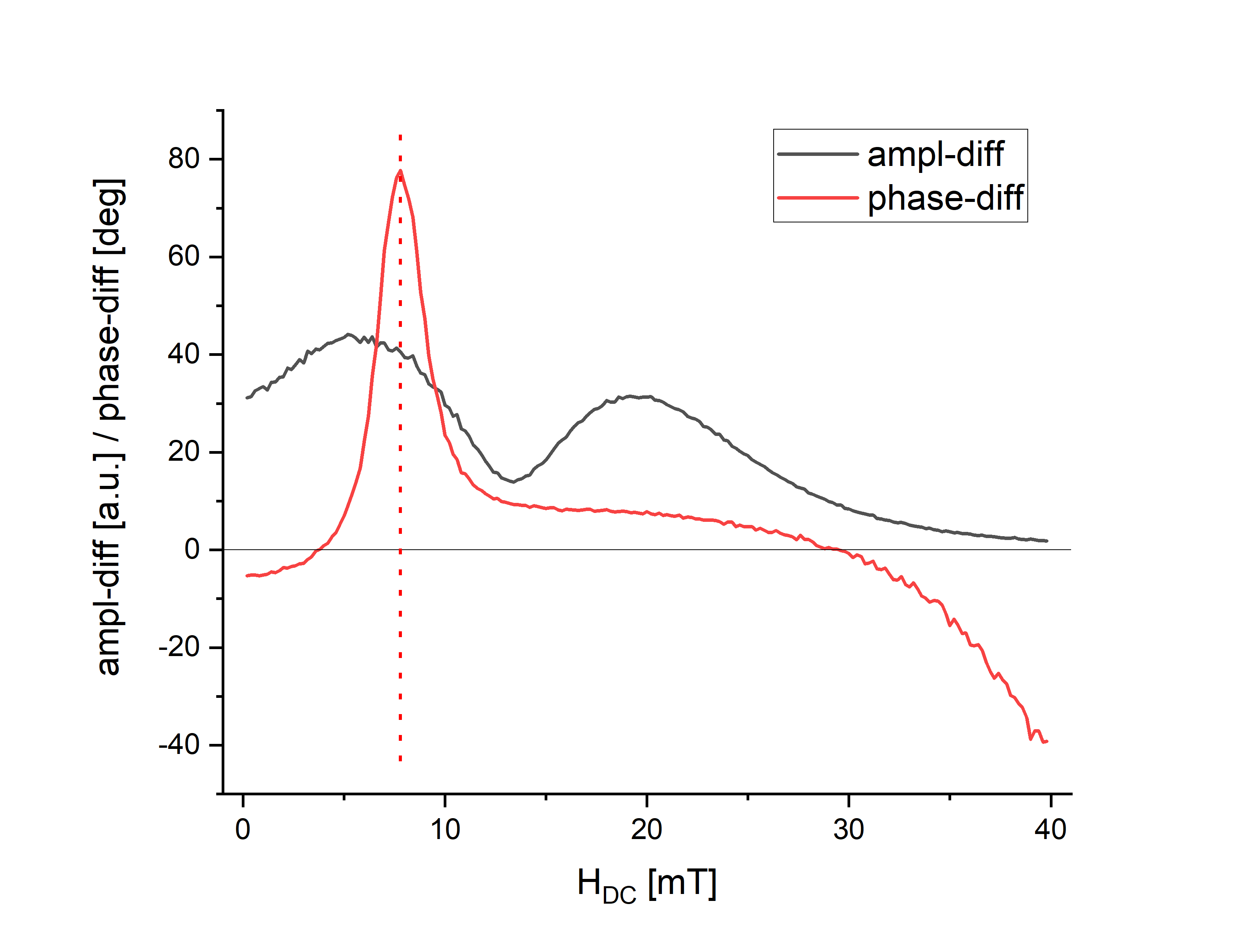


Supplementary figure 3. **Simulation of the COMPASS effect.** **Top** Offset field amplitude dependency of the 3^rd^ harmonic (real and imaginary parts) of a simulated data set. There is a difference between the signals due to the *ζ*-variation. **Bottom** The graphs show the difference of the amplitudes of both samples as well as the phase difference for the third harmonic. As expected, the highest sensitivity lies between the critical points of both samples (red dotted line).

In Fig. 3, an example is given for the 3^rd^ harmonic: Top shows the real and imaginary part of both MNP sample simulations. Due to the *ζ*-parameter variation, the crossing points of real and imaginary signals slightly shift as indicated in the zoom graph (top right). The difference in the amplitude (Fig. 3 bottom) of both samples shows a strong variation of about 5%. The phase difference plot (Fig. 3 bottom) shows an extremum, which means, that at this specific offset magnetic field strength the maximum phase difference between both samples is observable to distinguish the given samples.

To emulate the measurement with a magnetic field gradient *G* in the above-mentioned setup (Fig. 4 in the manuscript & Fig. 9 & Fig. 11), the signal needs to be integrated over offset magnetic field strengths *H*_DC_ present in the sample volume (Fig. 10))

|  | $\hat{S}(t)=\int_{a}^{b} \varrho\left( H_{\mathrm{DC}}+H \right)\cdot S(t, H_{\mathrm{DC}}+H) dH,$ | (5) |
| --- | --- | --- |

with *H*_DC_-*a*=*H*_DC_-Δ*H* and *H*_DC_+*b*=*H*_DC_+Δ*H* as minimum and maximum value of the *H*_DC_-range within the sample (Fig. 5). The density distribution *ρ* depends on the sample shape and gradient shape as well as MNP distribution throughout the sample.


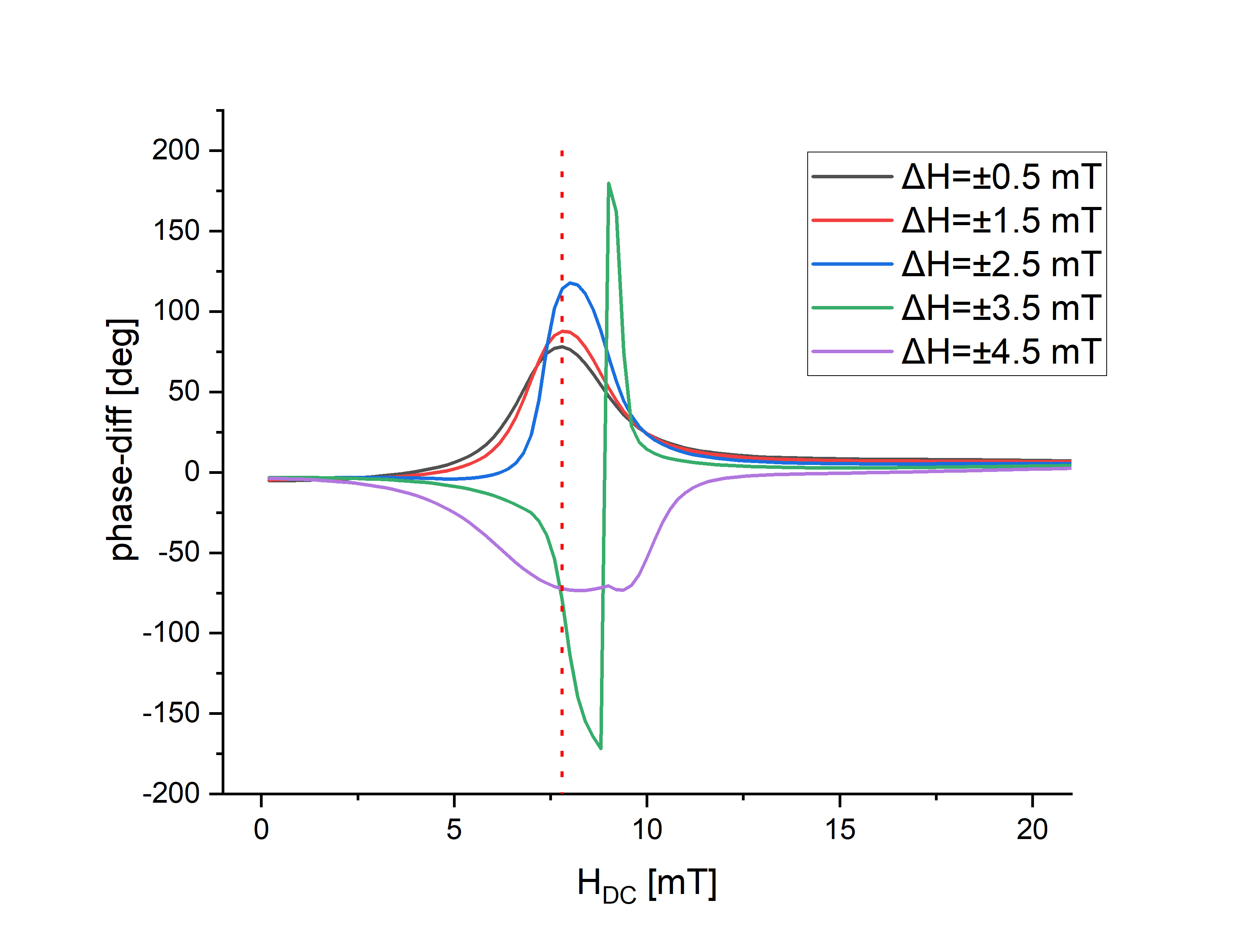


Supplementary figure 4. **Increasing the sensitivity by increasing the magnetic field gradient**. The simulated phase difference of the third harmonic of the two cumulated signals for different integration limits *a* and *b*. The integration limits are Δ*H*=±0.5 mT, Δ*H*=±1.5 mT, Δ*H*=±2.5 mT, Δ*H*=±3.5 mT, Δ*H*=±4.5 mT.

For integration, several parameters can be set: integration limits *a* and *b*, which depend directly on the magnetic field gradient *G* over the sample (range of the integration), and the offset magnetic field *H*_DC_, which defines the center of the integration window.

In Fig. 4, for several gradient strengths *G* (corresponding to different intervals [*a*, *b*]) the phase difference of the integrated signals is shown. For increasing *G* the phase difference between both samples increases significantly over a wider range of offset fields. This effect can be observed multiple times (number of critical points – CPs 🡪 *see supplementary Note 5*) for each harmonic for different offset magnetic fields *H*_DC_ and magnetic field gradients *G*.

***Optimal particle parameters***

The particle system (APTES-MNP-SBA-S1) used has a hydrodynamic diameter of about 330 nm. The size of an IgG S1 antibody is in the range of about 10 nm **^11^** and thus changes the diameter of the bound system approximately by 6% (330 nm 🡪 350 nm). This yields a change of the particle volume of about 19%. The expected change of the *ζ*-parameter would also be 19%, but the actual change will be lower since the magnetization is not necessarily blocked in cluster particles.

Optimal particles should have a blocked magnetization, i.e., they only show Brownian rotation. This is typically true for particles consisting of a single crystallite (magnetite and maghemite) with diameters of approximately 30 nm. With attached linker chemistry preferably with small sizes, e.g., nanobodies with a tenth of the size of conventional antibodies,**^12^** the relative *ζ*-parameter change per antibody will be proportionally much higher.

Furthermore, the concentration of the particle system may have influence on the signal. A lower concentration will catch a higher amount of targets per particle resulting in a higher signal change, but the signal itself is weaker. As the results of the serum experiments in Fig. 7 in the manuscript show, for high and medium antibody concentrations, the AB bindings seem to reach saturation. Thus, for the low concentrated serum, the particle concentration could be lowered to increase the signal change, but this would also increase the noise.

***Supplementary Note 3: APTES-MNP preparation and functionalization with S1 protein***

For initial testing, MNPs functionalized with APTES ((3-aminopropyl) triethoxysilane, Carl Roth, Germany) were used as an exemplary particle system. These MNPs are multicore particles whose crystallites show an average diameter of about 12 nm. They are coated with APTES and produced by alkaline precipitation as already described in Friedrich et al.**^13^** (Fig. 5). Briefly, synthesis procedure used iron (II) and iron (III) salts dissolved in di. H_2_O. Temperature was set to 70°C. After deoxygenizing with argon, 25% NH_3_ (aq) was injected into the iron salt solution. The now blackened SPION dispersion was further stirred for 5 min at room temperature. Next, 3 ml of APTES were added to the dispersion while mixing at 300 rpm. Solution was stirred for another 3 h at 450 rpm and a temperature of 70°C. Afterwards the SPIONs were cooled to room temperature and washed several times with water. Afterwards particles were redispersed in water.

The MNPs produced in this way have a hydrodynamic diameter of about 200 nm with the single crystallites showing a diameter of about 12 nm. SARS-CoV-2-S1 protein (SARS-CoV-2 (2019-nCoV) spike S1-His, Sino Biological, China) is covalently bound to the surface of the particles by binding SBA (N-succinimidyl bromoacetate) over cysteines present in the protein. A 0.05 M borate buffer with pH 8.5 was used during binding. The particle concentration during the functionalization processing was adjusted to 1 mg Fe/ml and 20 mM SBA dissolved in DMF (Carl Roth, Germany) was added. The samples were shaken for 2 h at 1,400 rpm. After that, the particles were washed several times with buffer solution. The obtained MNP-APTES-SBA were redispersed in borate buffer for binding of SARS-CoV-2-S1 protein. The samples were shaken again for 2 h at 1,400 rpm and washed several times with doubly distilled H_2_O. The final MNP-APTES-SBA-S1 possess a concentration of 10 µg S1 protein per 100 µg Fe (determined by UV-VIS measurements). After binding and the last washing step, the hydrodynamic size of the multicore particles (MNP-APTES-S1) is around 330 nm with a PDI of 0.3. Particles were stored in doubly distilled water until further use.

***Standard antibody detection assay***

***ELISA: sample preparation and measurement***

For human CoV-19 ELISA for S1 antibodies (RayBio, Peachtree Corners, GA), the S1 antibodies were diluted from 1,500 ng/ml to 25 ng/ml in the same buffer as used for the other tests. For the ELISA an amount of 100 μl sample were added to each well. After 1 h of incubation at RT under gentle shaking, all liquid was depleted, and the plate was washed four times with washing buffer. 100 μl of prepared Biotinylated Anti-Human IgG Antibody is added to each well followed by an incubation for 1 h as carried out before followed by another washing as described. Next 100 μl of prepared HRP-Streptavidin solution were pipetted into each well, followed by another incubation for 30 min. After washing 100 μl of TMB One-Step Substrate Reagent were applied to the wells. After further 30 min of incubation 50 μl of Stop Solution were added. The OD at 450 nm was detected using a plate reader (SpectraMax iD3, Molecular Devices).


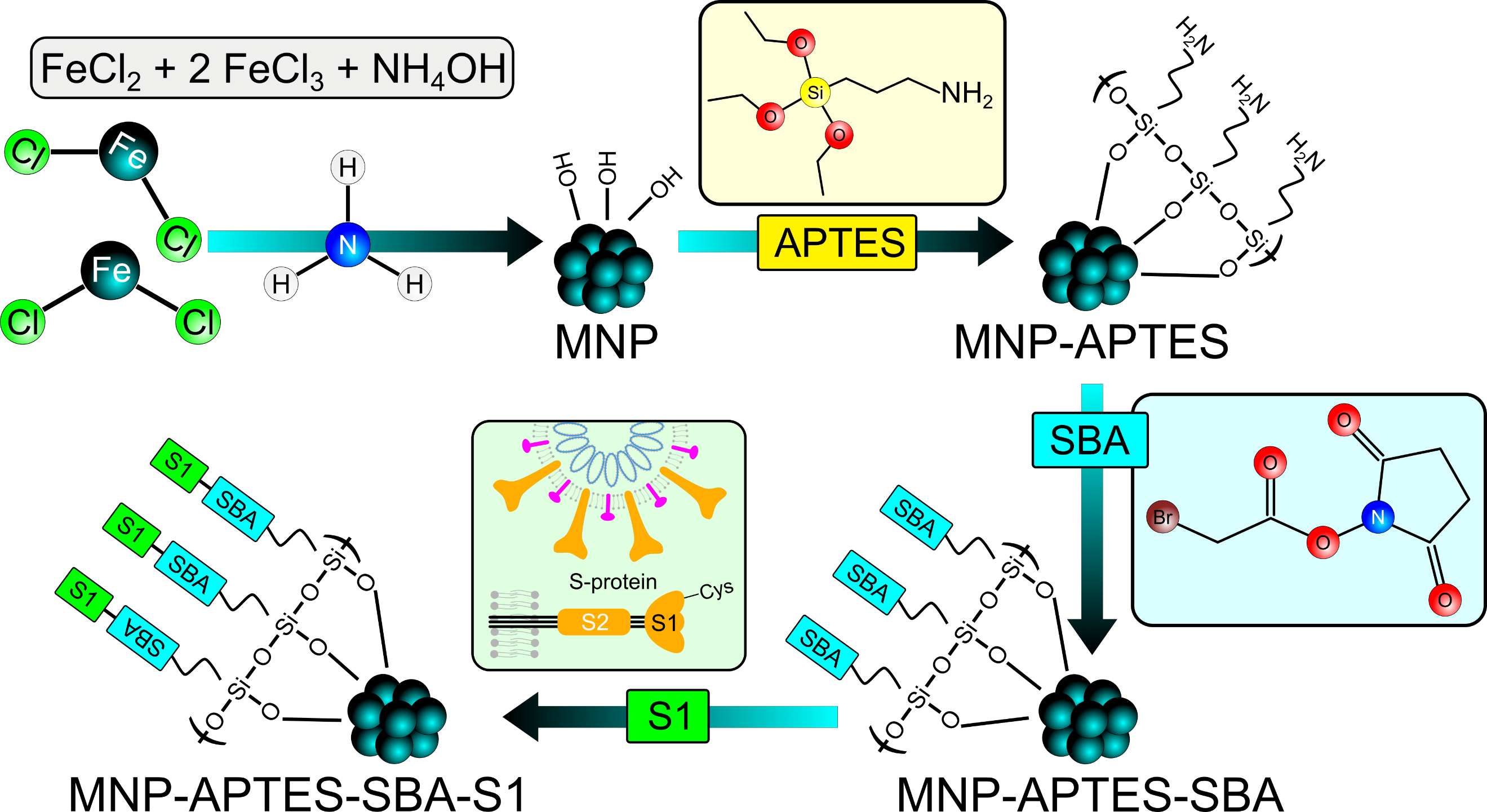


Supplementary figure 5. **Synthesis and functionalization route for MNP-APTES and MNP-APTES-S1 particles.**

***Particle based antibody detection assays***

***Flow cytometry analysis: sample preparation and measurement***

To test the binding selectivity to the correct antibody flow cytometry-analysis was performed with a Gallios flow cytometer (Beckman Coulter, Fullerton, CA, USA). First the antibodies (conc. 1 µg/ml SARS-CoV/SARS-CoV-2 Spike antibody (S1 antibodies, MW: 146.16 kDa), Chimeric Mab, Sino Biological, China) were diluted 1:2,000, 1:5,000, 1:10,000, 1:20,000 (last dilution corresponding to 50 ng/ml) antibodies in a buffer (PBS with 0.1% BSA). For the measurement 25 µl of MNP-APTES-S1 dispersions (iron conc. 100 µg Fe/ml) were added in an 0.5 ml Eppendorf cap. Subsequently 25 µl of antibody dilution or buffer (*ref*) were added. Samples were incubated for 1 h at 4°C. For washing, samples were centrifuged at 18,000 rcf for 10 min, the supernatant discarded and the MNPs redispersed in buffer. For detection, MNPs were further incubated with fluoresceinisothiocyanat (FITC) labeled protein A (1 µg/ml) for 1 h at room temperature (RT), which is known to bind specifically the Fc-region of IgG-antibodies.**^14^** Finally, the MNPs were washed as described before, redispersed and diluted 1:250 in buffer and analyzed for fluorescence by using flow cytometry. The fluorescence bleed through was eliminated by electronic compensation. The acquired data were analyzed with Kaluza software version 2.0 (Gallios, Brea, USA). The gating strategy can be found in Fig. 6.


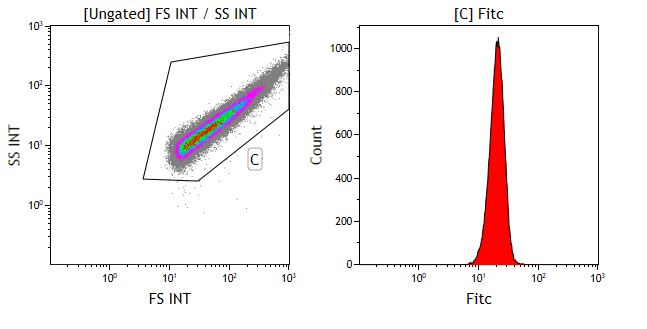


Supplementary figure 6. **Gating strategy for FACS analysis.**

***Sample preparation for COMPASS***

For the detection of S1 antibodies using the COMPASS device, the stock solution was diluted in buffer 1:2,000, 1:5,000 1:10,000, 1:20,000 and 1:200,000 (corresponding to 5 ng/ml for the lowest antibody concentration) as described before. An amount of 25 µl of MNP-APTES-S1 dispersions (100 µg Fe/ml) was added in an 0.5 ml Eppendorf cap. Subsequently 25 µl of antibody dilution (*S*+) or buffer (*ref*) were added. Samples were directly measured in the COMPASS device after careful mixing by pipetting without any further incubation times.

The results can be found in the *supplementary Note 8*.

***Supplementary Note 4: Parameter space evaluation***

To get a better understanding of the signal behavior at the critical points and the behavior of the magnetic field configurations, a more sophisticated experiment was performed to obtain a full data set. For that, the samples were measured with multiple offset magnetic fields *H*_DC_ as well as multiple excitation magnetic field strengths *H*_AC,j_(*t*) to obtain a data set over a defined range of both parameters. For that, an additional large solenoid with diameter of 30 cm has been placed around the AC-field generator box. The DC values were adjusted step-by-step by computer-controlled DC power supply (QPX1200L, Aim-TTi, UK).

The result is a 2D contour plot for real and imaginary parts of the MNP signal depending on the offset magnetic field *H*_DC_ and excitation magnetic field *H*_AC,j_(*t*) for each harmonic *n*.

The range of the offset magnetic field spans 0 mT up to 20 mT in 0.1 mT steps. The range of the excitation magnetic field spans 0.6 mT up to 21.6 mT in 0.3 mT steps. In sum 200·36=7,200 individual measurements were performed for each full data set. The basic excitation frequency was *f*_1_=20 kHz.

Fig. 7 shows a full data set of a reference sample as an example. With increasing excitation magnetic field *H*_AC_ (y-axis) the Chebyshev polynomial like shape as well as the nodes shows up more clearly. In the profile plot of the 3^rd^ harmonic for a specific excitation magnetic field *H*_AC_=17 mT, it can be seen, that around a node or critical point (real and imaginary part crosses near the x-axis) the phase shows a shift of about 180°.


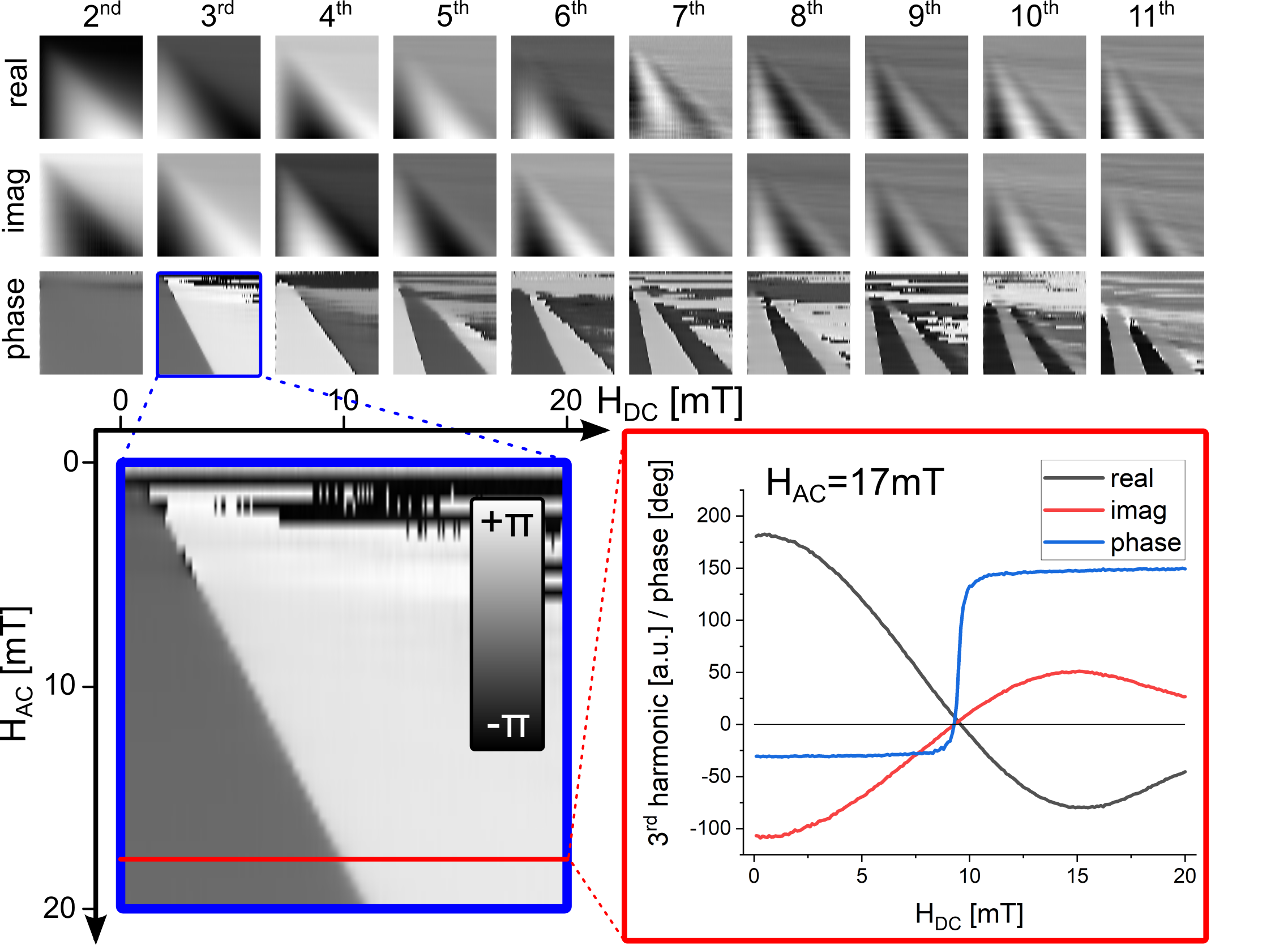


Supplementary figure 7. **Full data set acquisition.** Full data set (7,200 single measurements) for a reference sample. **Top** Real, imaginary and phase 2D contour plot for the 2^nd^ to the 11^th^ higher harmonic of *f*_1_=20 kHz. **Bottom** Exemplary phase plot of the 3^rd^ higher harmonic and the real, imaginary and phase profile for a specific excitation magnetic field *H*_AC_=17 mT.


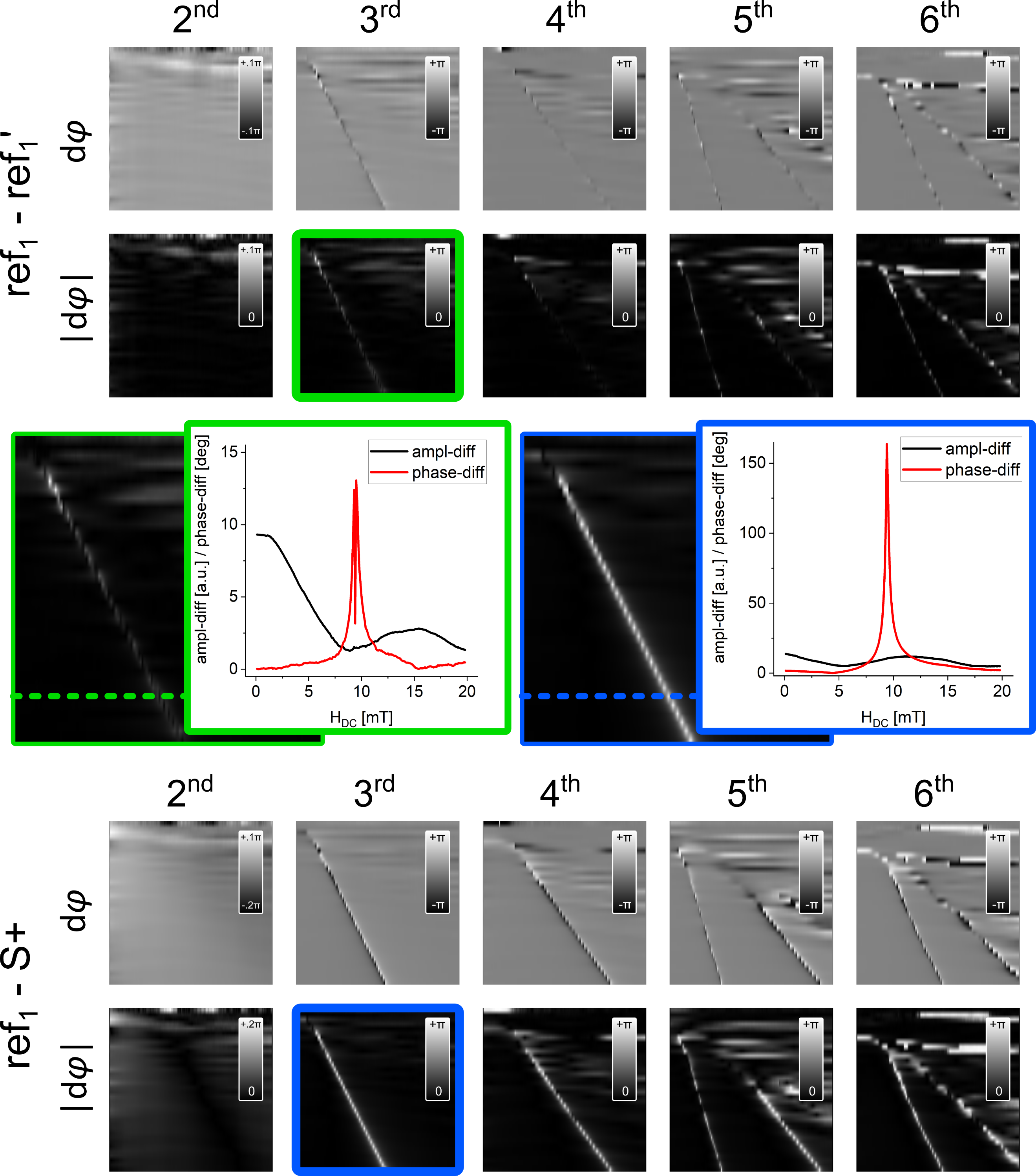


Supplementary figure 8. **Subtraction plots of three full data sets**. **Top** The phase difference for higher harmonics (2^nd^ to 6^th^) of two reference samples (*ref*_1_-*ref*_1_’) are shown (first row). **Bottom:** the phase difference for higher harmonics (2^nd^ to 6^th^) of a reference sample and a binding sample (*ref*_1_-*S*+) are shown (first row). For better visualization, the absolute values are plotted (second row). **Middle** Comparison of the 3^rd^ higher harmonic for both subtractions: the increased phase difference can be clearly seen for the binding sample.

With this full data set, a more specific view on the COMPASS effect can be visualized when subtracting these data from different samples. In Fig. 8, the subtraction was performed for three data sets: subtraction between two reference samples (*ref*_1_-*ref*_1_’) and subtraction between a reference sample and a binding sample (*ref*_1_-*S+*). In the top rows, the phase differences for each higher harmonic are shown. For a better visualization, the absolute values for the phase differences are provided in the second row. For example, the data of the 3^rd^ harmonic of each difference (*ref*_1_-*ref*_1_’) and (*ref*_1_-*S*+) are shown in more detail in the center: at a first glance, along the Chebyshev nodes high differences can be seen. Even in the difference between both reference samples this is visible. But a view on the profile plot along a specific excitation magnetic field strength (*H*_AC_=18 mT) shows a 13 times higher phase difference peak for binding versus reference. It must be mentioned that the difference of the amplitude, which is also provided in the profile plot, also shows a 10 times higher signal at the position of the phase difference peak. Combining both parameters (amplitude and phase) provides a 13·10=130-fold increase in the difference between binding and non-binding samples.

Furthermore, the graph in Fig. 9 offers even more information about the onset of exploited signal behavior. Above an excitation magnetic field amplitude *H*_AC_ of about 2.5 mT and an offset magnetic field *H*_DC_ of about 2 mT, the phase difference is clearly detectable. Below these values, the signal can be still measured but with much less SNR. This is a possible explanation why this effect was not reported before in ACS experiments or common MPS experiments.


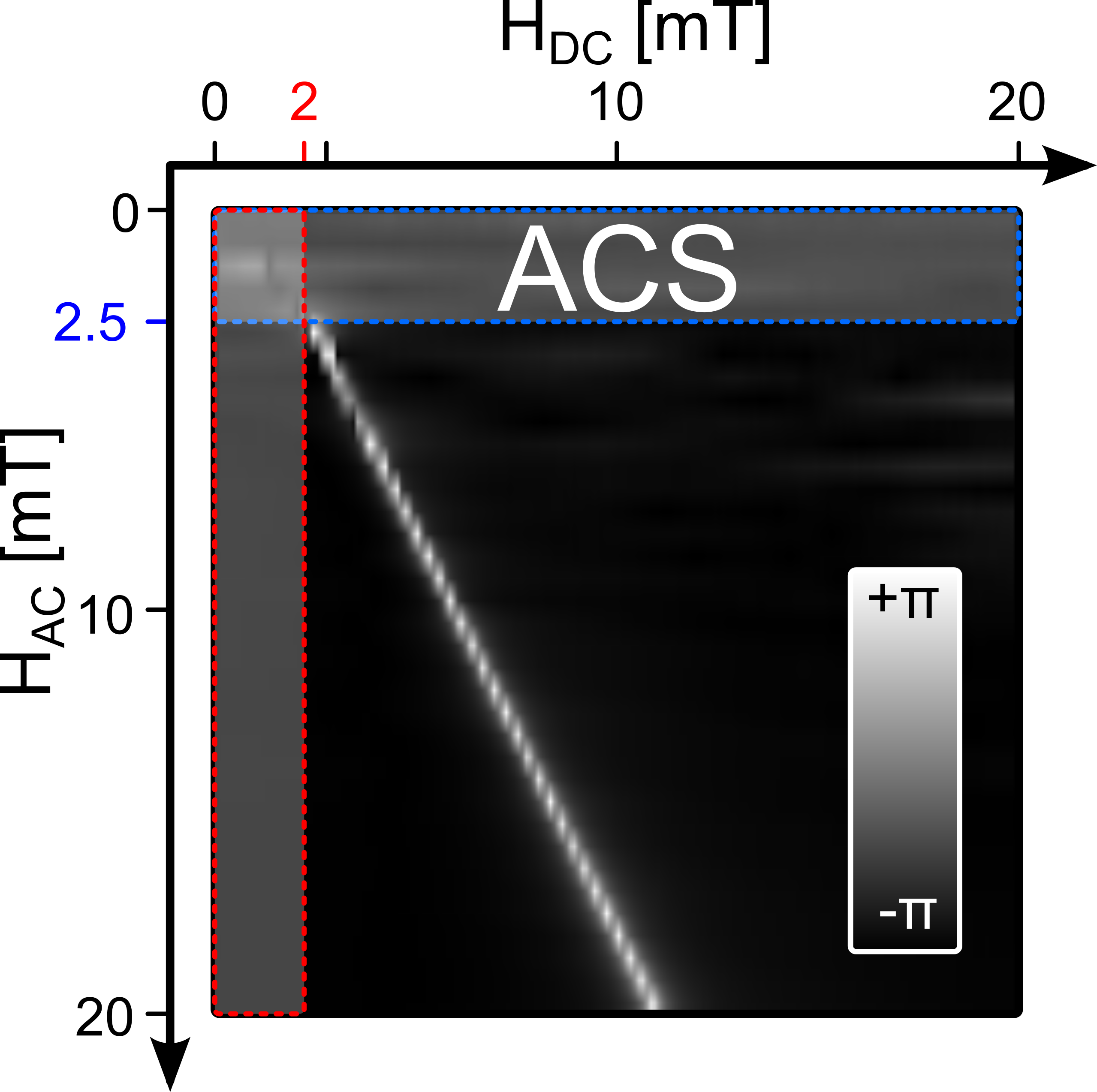


Supplementary figure 9. **Parameter space for COMPASS.** A closer look at the absolute phase difference of the 3^rd^ harmonic between a reference sample (*ref*) and a binding sample (*S*+) indicates the ‘starting points’ of the proposed COMPASS effect. Below *H*_DC_=2 mT offset magnetic field and *H*_AC_(*t*)=2.5 mT excitation magnetic field strength, the effect is much weaker and cannot be measured.

***Supplementary Note 5: Mobile COMPASS device***

The measurement system is similar to common MPS devices as known from the literature.**^15, 16^** Only few modifications turn the MPS device into a highly sensitive MNP measuring COMPASS device. The first is adding a strong magnetic field gradient along the area of interest (measurement chamber) of the MPS device. In case of the proposed mobile modified MPS device, a strong permanent magnet (10 mm³ Neodym N52) is placed near the transmit/receive (tx/rx) unit. In Fig. 10, a CAD drawing of the modular tx/rx unit is shown with the positioned magnet. The bore provides space for 0.5 ml samples (Eppicaps). The gradiometric receive coils are placed as closely as possible to the sample to guarantee high SNR. Only one receive coil receives signal from the sample, while the second one is wired antiparallelly to suppress the signal induced directly by the excitation coil as well as environmental noise.**^17^** The transmit coil (tx) is placed around the receive coils. To adjust the gradiometric receive coil, the entire rx-inset can be moved and adjusted in relation to the tx-coil to suppress the excitation signal as much as possible (at least 60 dB). The coil parameters for transmit coil are 4×9 windings (90×0.1 mm litz wire, Rupalit, Pack LitzWire, Germany) resulting in a magnetic field of 2.1 mT per ampere. The receive coil uses 20 windings for each coil (12×0.04 mm litz wire, Rupalit, Pack LitzWire, Germany) resulting in a resistance of about 2 Ω. The transmit chain is optimized for 20 kHz and can generate magnetic fields up to 50 mT with a current of approximately 25 A.

To avoid heating issues and thus potential instabilities, which can affect the signal quality, the transmit system is typically driven in a pulsed mode with a low duty-cycle of 20 ms/1,000 ms=5%.


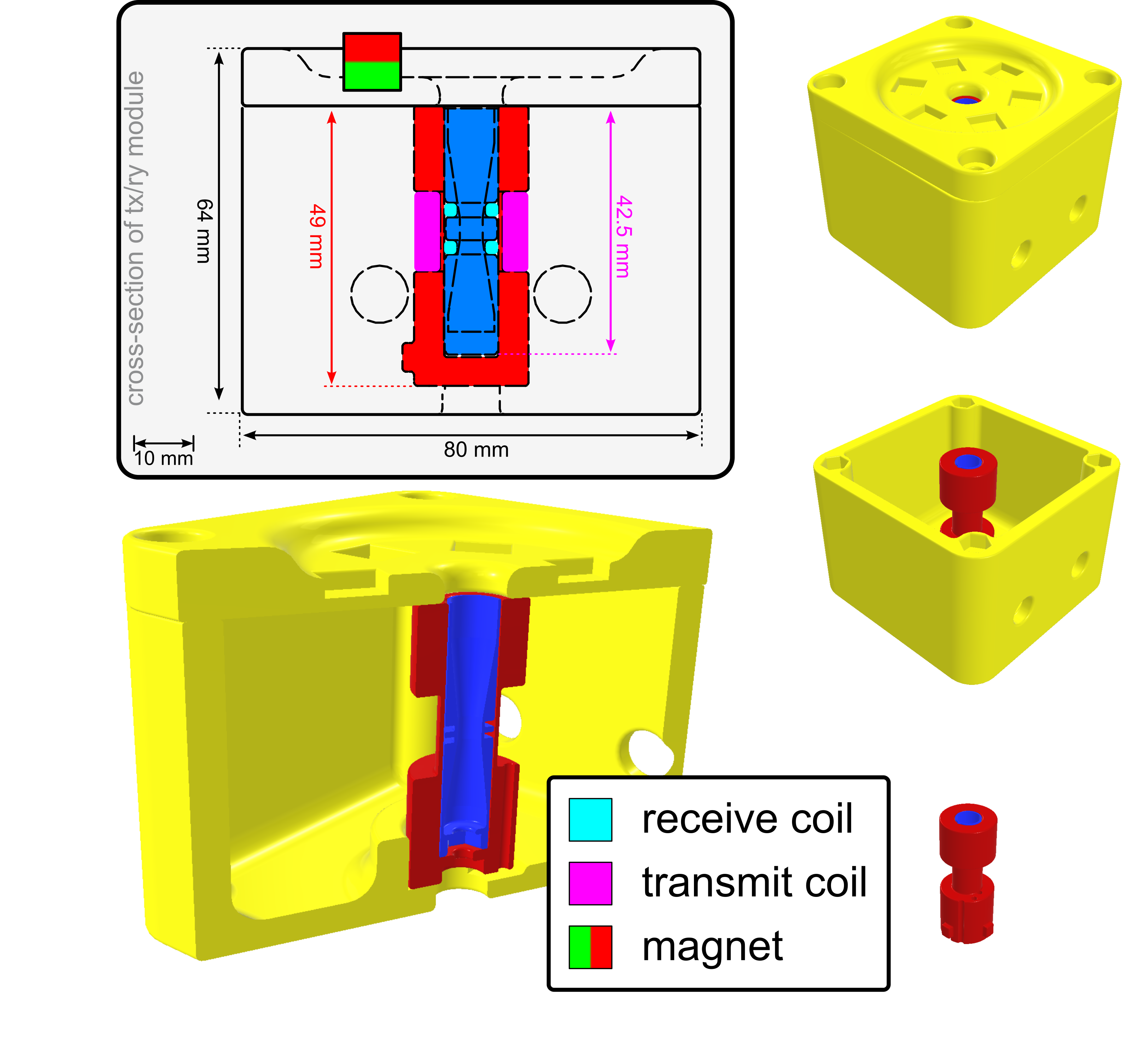


Supplementary figure 10. **CAD drawing/3D-renderings of the tx/rx module**. The modular box consists of a transmit coil, an adjustable gradiometric coil and a permanent magnet for magnetic field generation within the measurement chamber. The tx/rx module can be connected via two BNC adapters to the main system.

The tx/rx module is connected via two coaxial cables to the main device, which serves as stand-alone system consisting, among other parts, of a microcontroller (PSoC 5LP, Cypress Semiconductors, USA – firmware generation with PSoC Creator 4.4), a BLE module (HC-05, AZ-Delivery, Germany), an audio amplifier (TDA7294, STMicroelectronics, Switzerland), and rx-filter components. The power supply, here a battery pack and can be connected via a standard USB-B connector.

The positioning of the permanent magnet (Neodym N52) generates in the sensitive area, the center of the tx/rx module, a strong gradient magnetic field. In Fig. 11, a simulation of the magnetic field vectors is presented for the entire volume of the gradiometric receive coil, where only in the upper area the sample is positioned.**^18^**


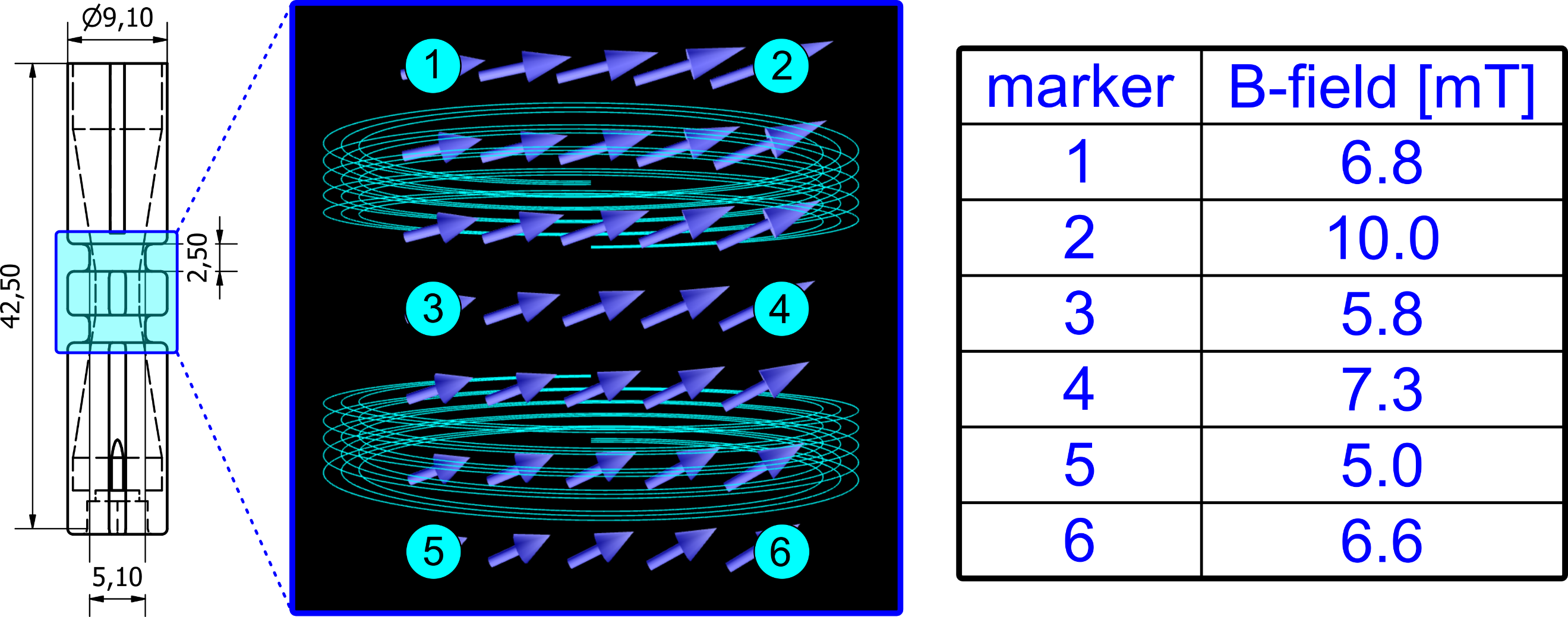


Supplementary figure 11. **Magnetic field gradient strength within the gradiometric receive coil or measurement chamber**. The magnetic field strengths are in the range between 5.8 mT to 10.0 mT for the upper area of the gradiometric rx coil.

Four buttons and a 2-line display are used for setting up the device, perform measurements and display all required results without using additional hardware components. For additional information about the experiments, such as signal graphs or debugging information, all data are broadcasted via Bluetooth and can be analyzed via mobile equipment and a dedicated host software (Embarcadero RAD Studio 11). Fig. 12 shows on the left the mobile COMPASS device. At the right side, some pictures show the real tx/rx module, the tx/rx coil system and the entire box with additional permanent magnets. Most parts are 3D-printed with FDM (N2, Raise, USA) or SLA (Form3, Formlabs, USA) method.


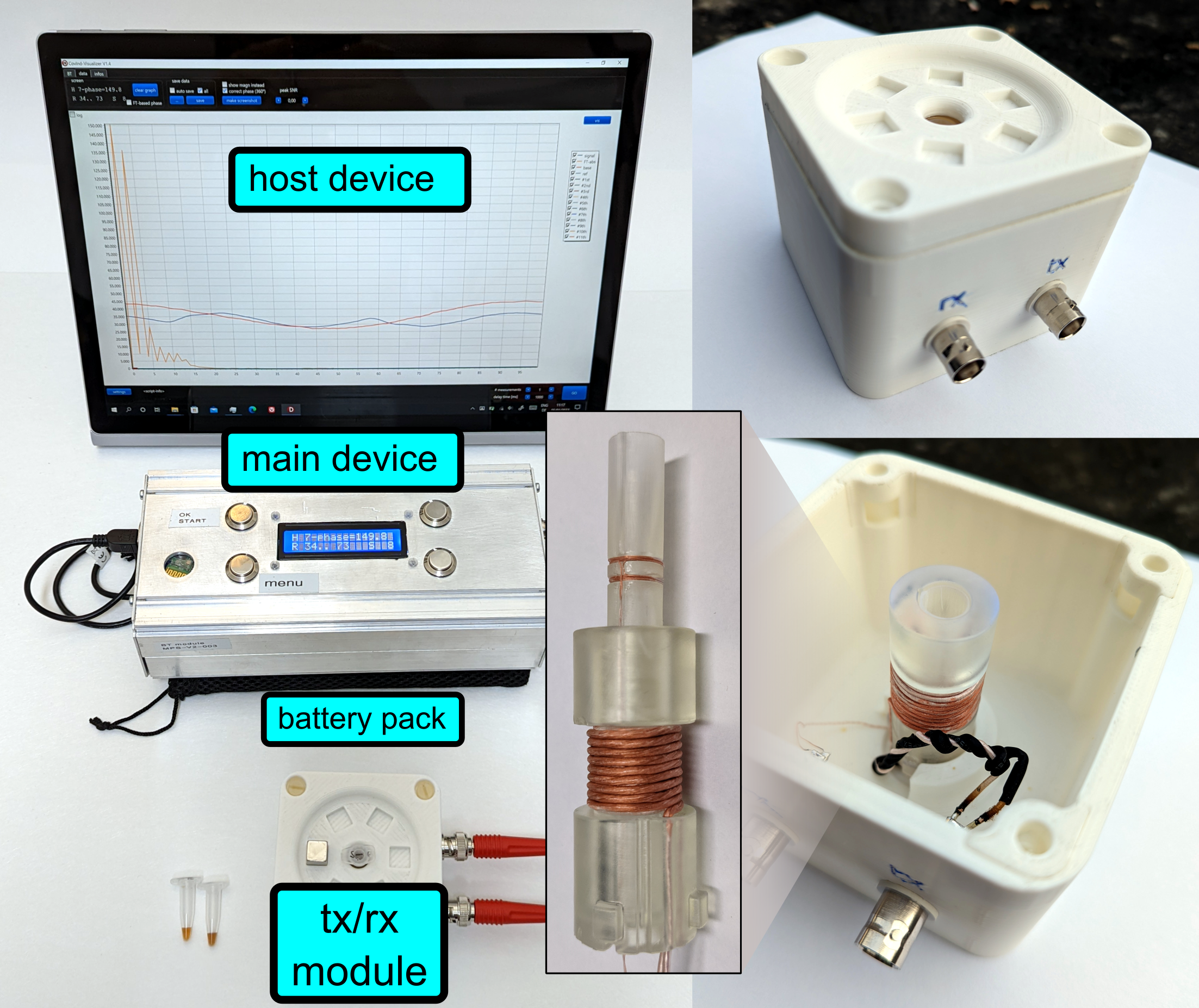


Supplementary figure 12. **Pictures of the mobile COMPASS device**. **Left** Mobile modified MPS device with the main device consisting of all necessary hardware components such as microcontroller, amplifier and filters, the transmit/receive module for 0.5 ml Eppicaps and a battery pack as power supply. **Right** Pictures of the tx/rx module and the inner coil system.

***COMPASS operating points***

To set up a COMPASS device for highly sensitive measurements, the optimal operating points must be adjusted. As mentioned before, critical points (CPs) are found at specific field ratios of *H*_AC_ and *H*_DC_ (*H*_AC_>*H*_DC_) for each higher harmonic *n*, where the number of nodes *N*_n_ per harmonic depends on the harmonic number n

|  | $N_{n}=n \mathrm{div} 2 \mathrm{for} n> 0$. | (6) |
| --- | --- | --- |

With that, critical points CP_n,m_ can be uniquely assigned.


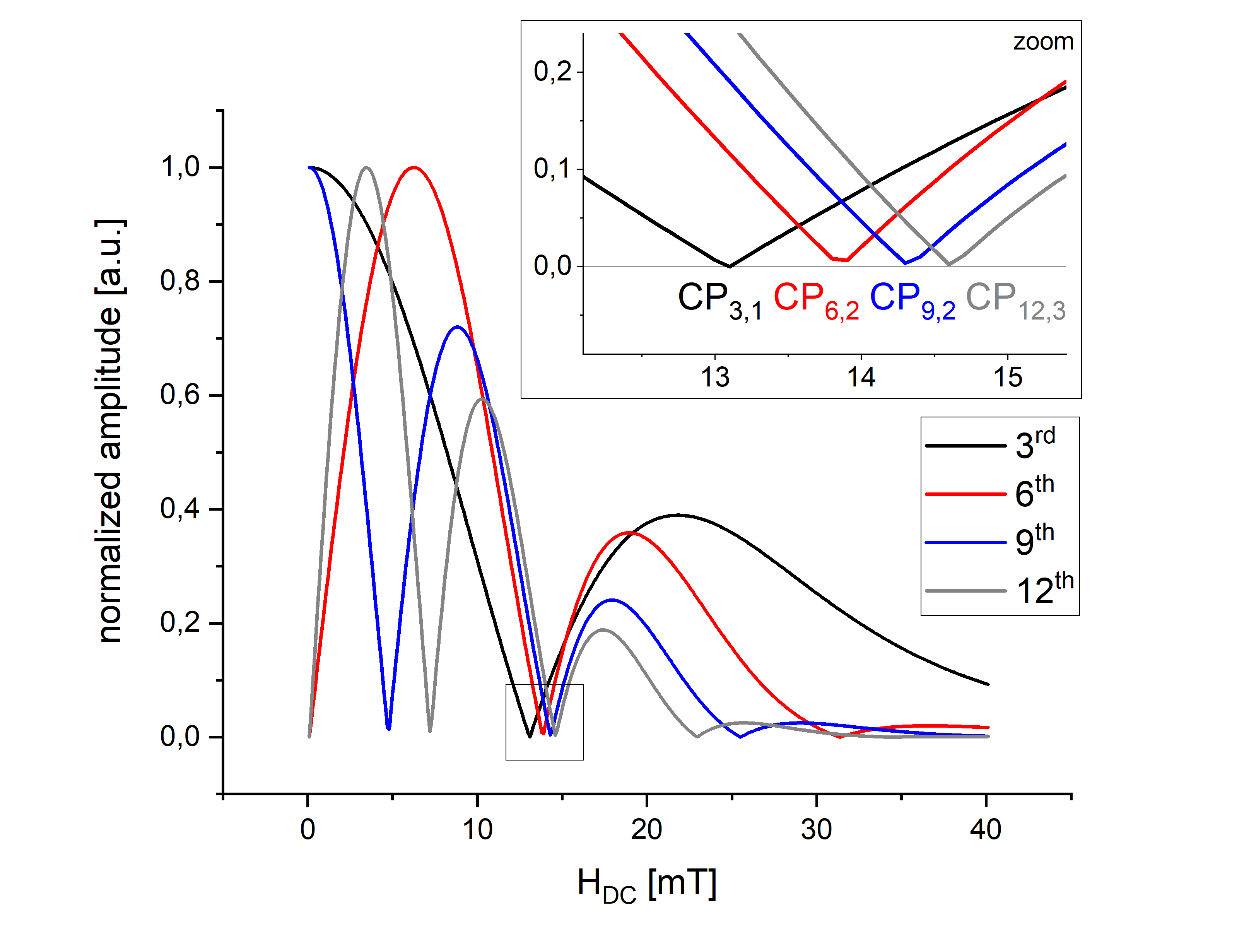


Supplementary figure 13. **Critical point distribution for different harmonics**. Amplitude plots *A*_n_(*H*_DC_) of simulated data for the 3^rd^, 6^th^, 9^th^, and 12^th^ harmonic show multiple critical points CP_i,j_ (*H*_AC_≈40 mT). With a limited set of harmonics, areas with high density of CPs can be found.

The graph in Fig. 13 shows normalized absolute plots *A*_n_(*H*_DC_) of the 3^rd^, 6^th^, 9^th^, and 12^th^ harmonic of simulated data. For a limited selection of harmonics, which is the case for realistic receive chains due to signal-to-noise limitation as well as the resonant behavior of the receive chain, the critical points accumulate in specific areas, e.g., CP_3,1_, CP_6,2_, CP_9,2_, CP_12,3_. The operation point of a COMPASS device should preferably be chosen to cover such an area to analyze critical points for as many harmonics as possible for higher robustness and redundancy.

For calibration, three steps are required:

1. Placing a reference sample in the COMPASS device.
2. Set up the offset magnetic field *H*_DC_ by positioning and/or adjusting of a permanent magnet or electromagnet.
3. Perform an amplitude sweep for *H*_AC_ to find the desired operating point.

***Supplementary Note 6: Data processing***

Data processing is performed completely within the PSoC microcontroller (PSoC 5LP, Cypress Semiconductors, USA) using the on-chip resources. The digital-to-analog (DAC) converter generates a customizable sinusoidal excitation signal (data length of 10,000 samples at a sampling rate of 1 MS/s, which corresponds to 10 ms length) feeding the audio amplifier (TDA7294, STMicroelectronics, Switzerland) for generation of the required time varying magnetic field. After passive filtering and pre-amplification two onboard 12 bit analog-to-digital (ADC) converter are used for interleaved data acquisition of the incoming signal from the gradiometric coil. Controlling both the transmit and receive channel with the same chip and a single reference clock results in a low phase jitter and allows for high resolution and accuracy even with the comparatively low sampling rates (2 MS/s) and bit-resolution values. The acquired data are directly processed on the chip. In a first step, the entire data set (20,000 sample points) is cut into sub data sets containing 100 samples each corresponding to one full period of the excitation frequency of 20 kHz. This reduces the on-chip calculation time for the following sine and cosine transformation to a few milliseconds without sacrificing SNR and enables repetition times below 1 s. A combined sine and cosine transformation for the first eleven higher harmonics is used to calculate the phase and amplitude for each harmonic.

For an independent data visualization, a 2-line display in the main device is used. The data can also be broadcasted automatically through an external host software to an external device like a desktop computer or laptop for evaluating additional information and for better visualization.

***Supplementary Note 7: Measurement protocol for COMPASS***

The measurement protocol for testing with COMPASS is optimized for receiving clear and robust results rapidly within seconds. For that, a differential measurement of two identical probes split from the original prepared MNP-APTES-S1 batch is used (Fig. 14 (1)). The to-be-measured substance is directly given in the sample probe (Fig. 14 (2)) and can be instantaneously measured with COMPASS without further washing processes or conjugation or incubation times (Fig. 14 (4)). The same amount of a buffer solution is added in the reference sample (Fig. 14 (3)) before being measured with COMPASS (Fig. 14 (4)). The results are available within seconds (Fig. 14 (5)).


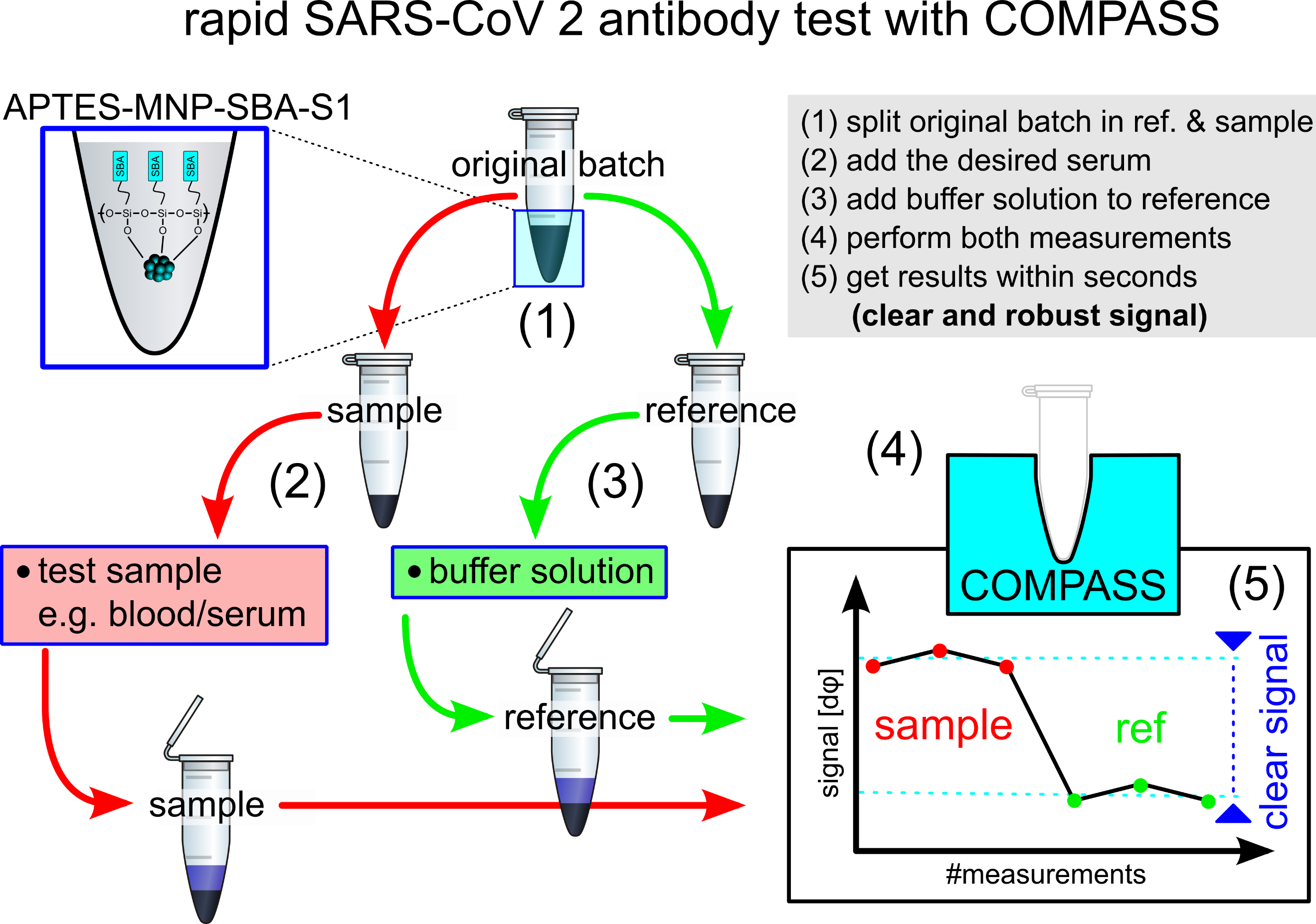


Supplementary figure 14. **Sketch of the measurement protocol**. Starting from a prepared MNP-APTES-S1 batch, two identical probes are prepared: **sample** and **reference** (1). The to-be-measured substance is directly added in the sample vial without any further washing step or incubation time (2) before being measured in the COMPASS device (4). The reference sample is filled with buffer solution (3) and measured (4). Within seconds, a clear and robust result is available (5).

It has to be mentioned, that the antibody-antigen reaction kinetic time scale is in the range of minutes and less (supplementary Note 9), while a major part of the binding processes happens within the first seconds after combination.**^19-21^**

***Supplementary Note 8: Sensitivity compared to ELISA and flow cytometry-analysis***

To evaluate the sensitivity of the proposed methods within the flexible MPS device, a comparison with ELISA as well as flow cytometry-analysis were performed.

For flow cytometry, a sample series with antibody dilutions of 1:2,000, 1:5,000, 1:10,000, and 1:20,000 (corresponding to 50 ng/ml antibodies) as well as a reference sample (*ref*) and a non-binding sample (1:2,000) with a MERS antibody (neg. control) were prepared and measured within a flow cytometer and the modified MPS device. The results in Fig. 15 show a sensitivity of antibodies detection of dilutions between 1:2,000 and 1:10,000.


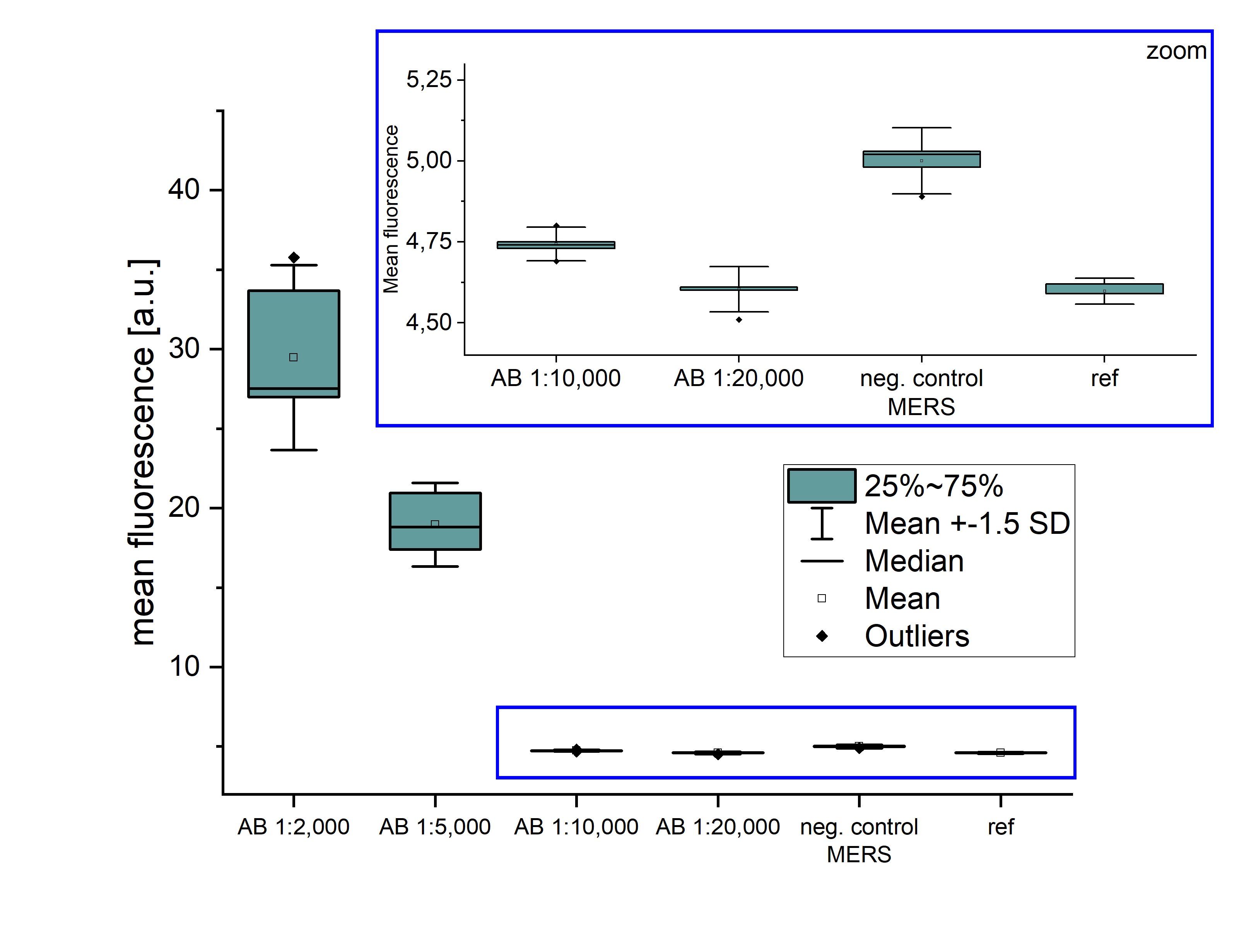


Supplementary figure 15. **Measurement results of flow cytometry-analysis.** Mean fluorescence of MNP-APTES-S1 after incubation with antibodies and Protein A-FITC for different dilutions (sample number *n*=9). Detection of antibodies was possible up to a dilution of 1:10,000 with flow cytometry-analysis.

In Fig. 16, the results of the COMPASS experiments are shown. Top: The single experiments of the measuring sequence (*ref*, 1:2,000, 1:5,000, 1:10,000, 1:20,000, neg. control) show a clear trend in phase difference of the 7^th^ and 9^th^ higher harmonics. Each sample was measured 5 times without any averaging (acquisition time 10 ms each). A closer look at the values reveals a sensitivity, which lies beyond the flow cytometry measurements.


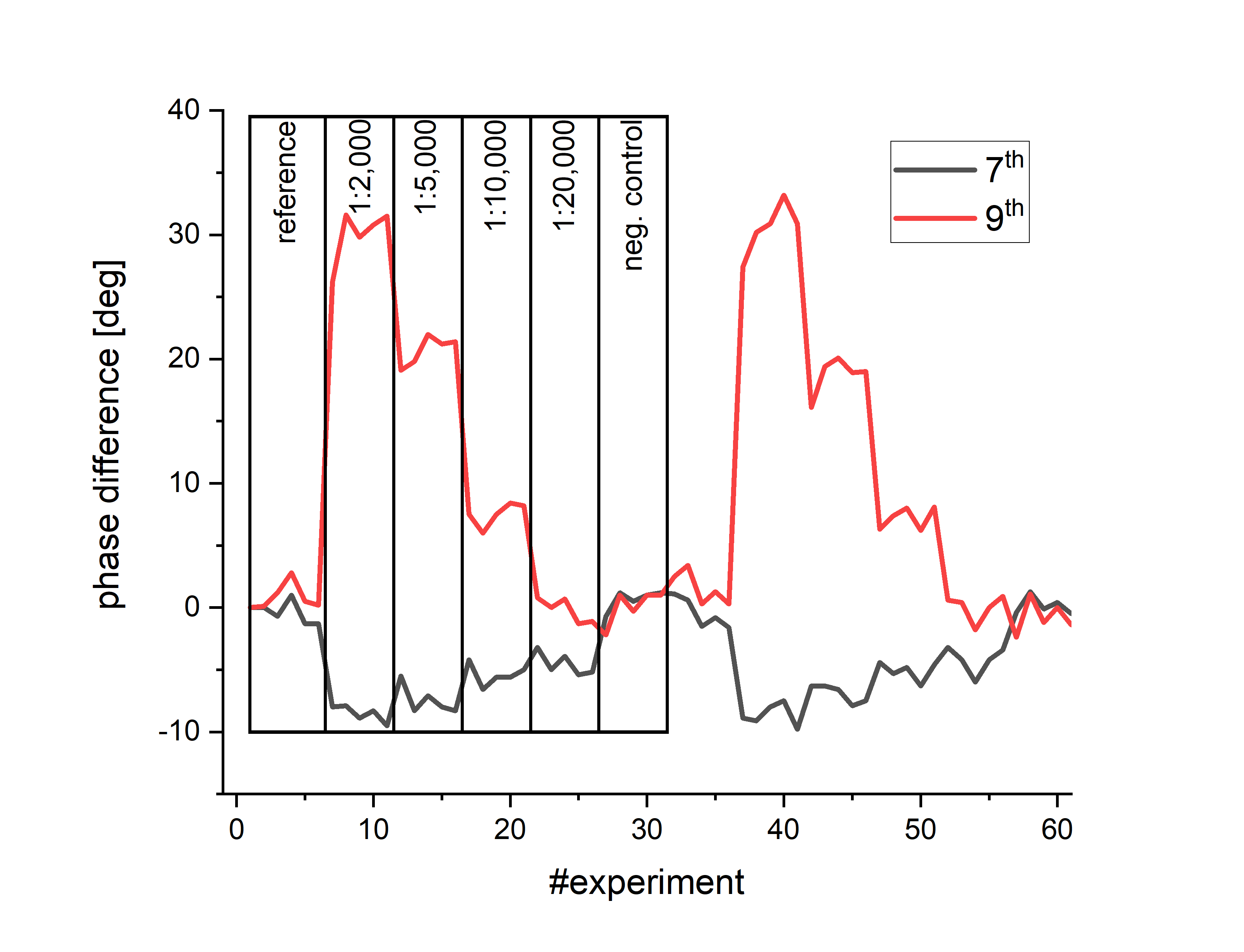


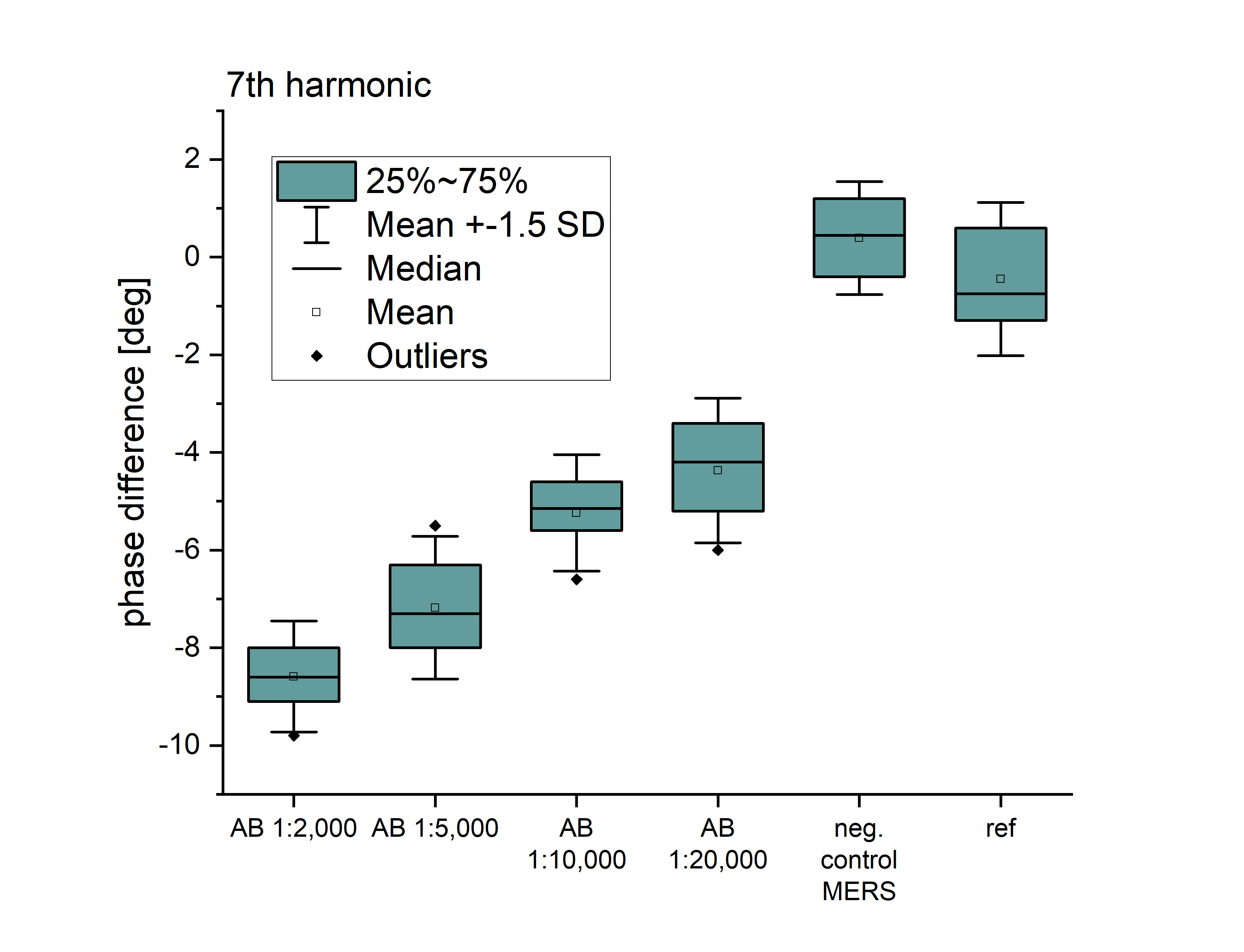


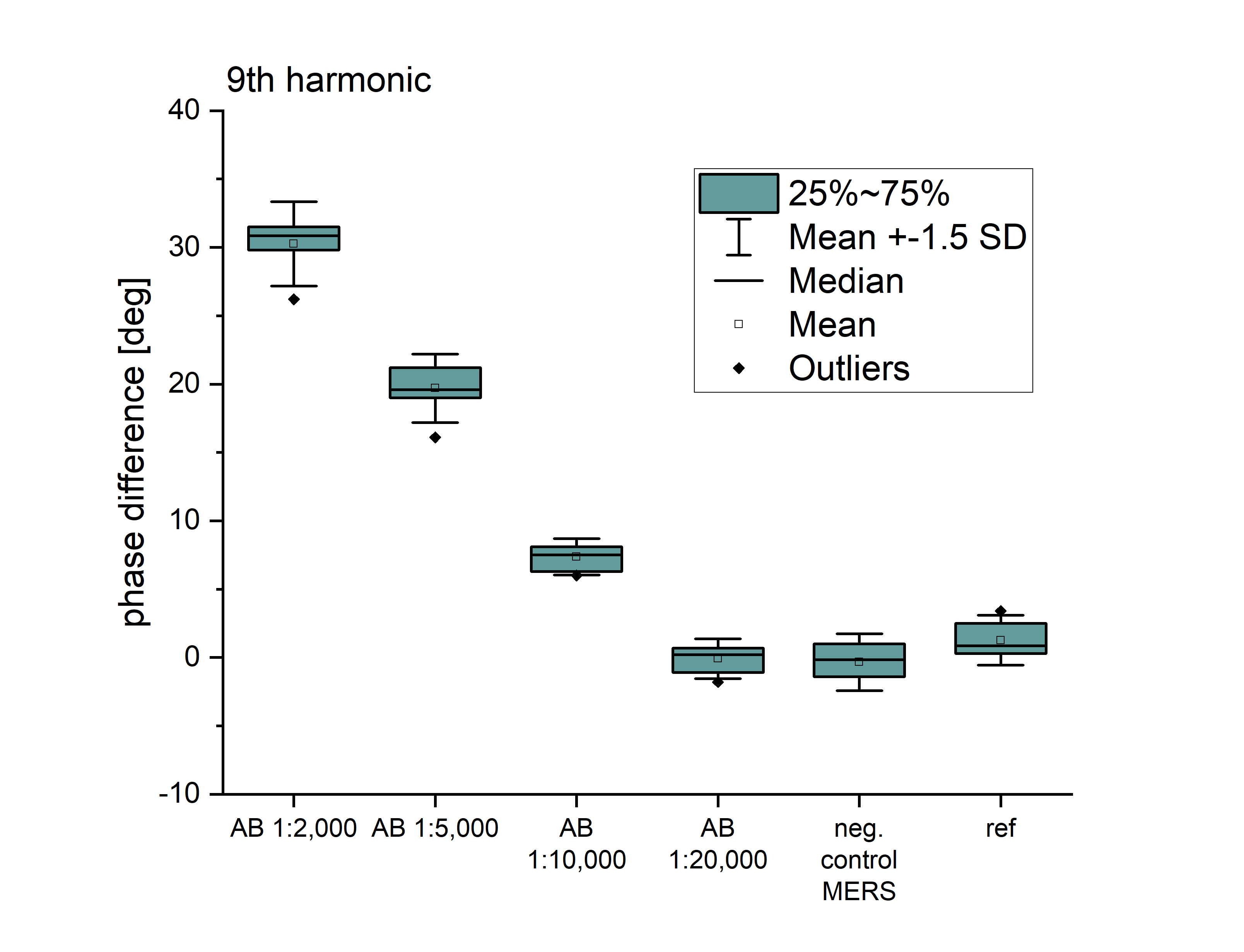


Supplementary figure 16. **Results of measuring the dilution series within the COMPASS device using MNP-APTES-S1**. **Top** The phase differences of the 7^th^ and 9^th^ harmonic are shown for the measuring sequence: reference (*ref*), dilutions 1:2,000, 1:5,000, 1:10,000, 1:20,000, and neg. control, 5 times each, repeated 2 times. **Center and bottom** A closer look at the data reveals the sensitivity of this method. The single measurements (*n*=5) for each measurement quintet have been statistically evaluated.

For comparison, a standard SARS-CoV ELISA test (RayBio® COVID19 S1 RBD protein Human IgG ELISA Kit Protocol, RayBiotech, Inc.) was performed according to the manufacturer’s instructions. Due to low signal, the incubation time was extended to 18 hours instead of 30 minutes.

In Fig. 17, the result of the ELISA test is shown. The averaged optical density (OD) at 450 nm of the wells (triplets) were determined and show a sensitivity limit at 25 ng to 50 ng antibodies per ml corresponding to 1-2 BAU (binding antibody unit).


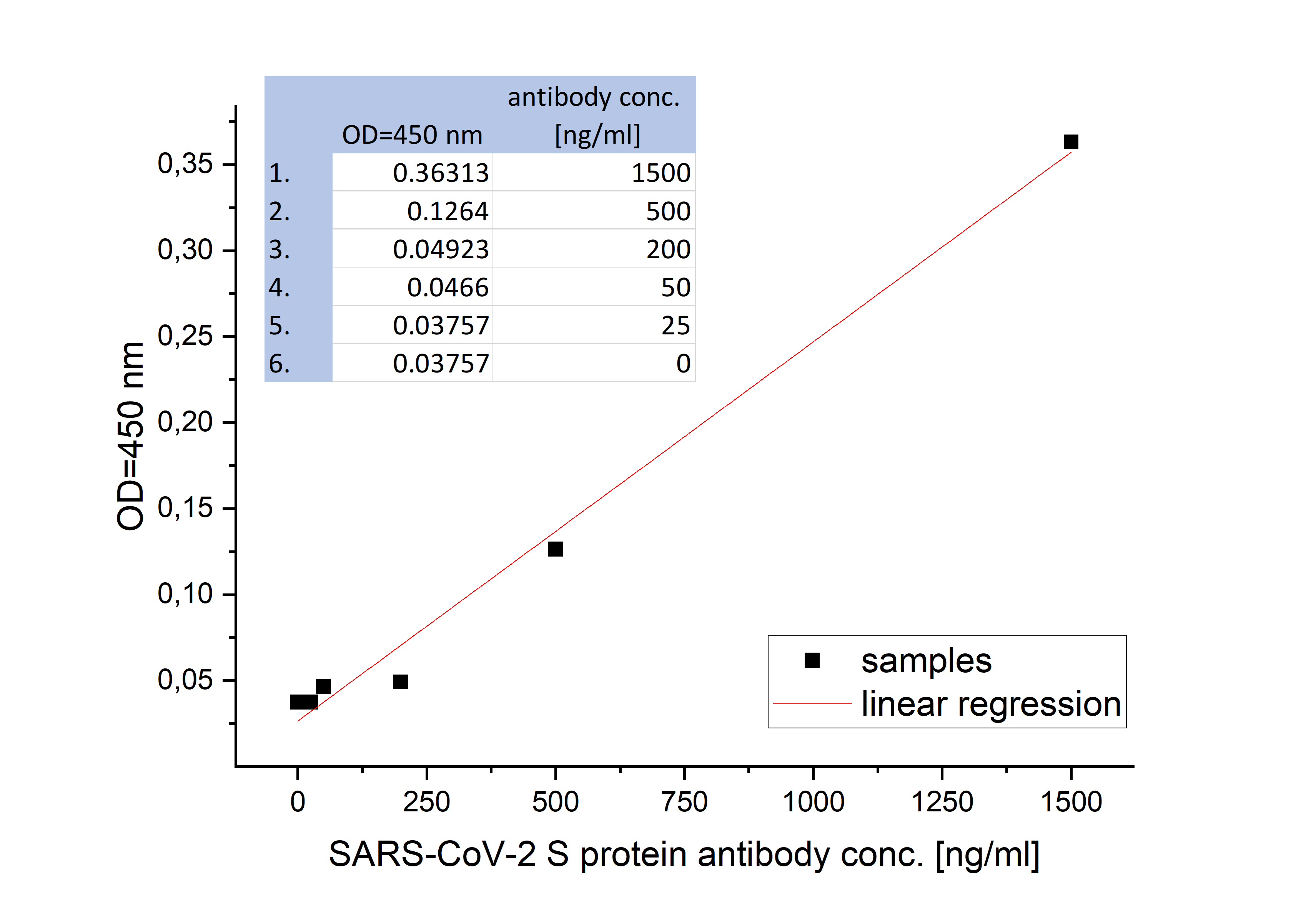


Supplementary figure 17. **Result of a standard SARS-CoV-2 S1 protein IgG test (ELISA)**. The averaged optical density (OD) at 450 nm of the wells (triplets – *n*=3) were measured and shown.

In conclusion, the sensitivity of the provided dilution series measured with the COMPASS device can be determined to about 50 ng/ml antibodies, which corresponds to a BAU of about 2. With a weight of 150 kDa per antibody and a sample size of 50 µl (25 µl MNP + 25 µl ABs), a sensitivity of about 8.3 fmole per 50 µl-sample (1.25 ng per 50 µl-sample or ~167 pM) can be calculated, which is also comparable to the sensitivity of ELISAs.

As indicated in Fig. 6 in the manuscript, using a COMPASS device optimized to a specific critical point (S5: COMPASS operating points), the sensitivity is improved at least by a factor of 10 and reaches 0.33 fmole/50 µl ≙ ~7 pM.

***Supplementary Note 9: Antibody-antigen binding kinetics***

Since the binding kinetics is an essential part for a rapid testing protocol, in Fig. 18 initial results measuring the real-time binding kinetics of APTES-MNP-SBA-S1 + MERS antibodies (neg. control) and SARS antibodies are presented. The graph shows the phase progression over time with measurements in time steps of 1 second for the 5^th^ harmonic. At time point 20 and 110, 25 µl MERS antibodies (dilution 1:50k – 20 ng/ml) as well as 25 µl SARS antibodies (dilution 1:50k – 20 ng/ml) has been added.

As expected, the addition of MERS ABs (neg. control) only slightly changes the phase, but the addition of SARS ABs almost instantaneously changes the phase dramatically. The phase change exceeds 10 standard deviations within a few seconds, which is a very conservative detection limit.**^22^** Saturation is reached within 75 seconds, which is comparable with the literature.**^19-21^**

Considering binding kinetics, the 3D accessibility of nanoparticles is inherently higher than for flat surface immobilized bait proteins (cf. 2D samples used in ELISA or flow-cytometry).


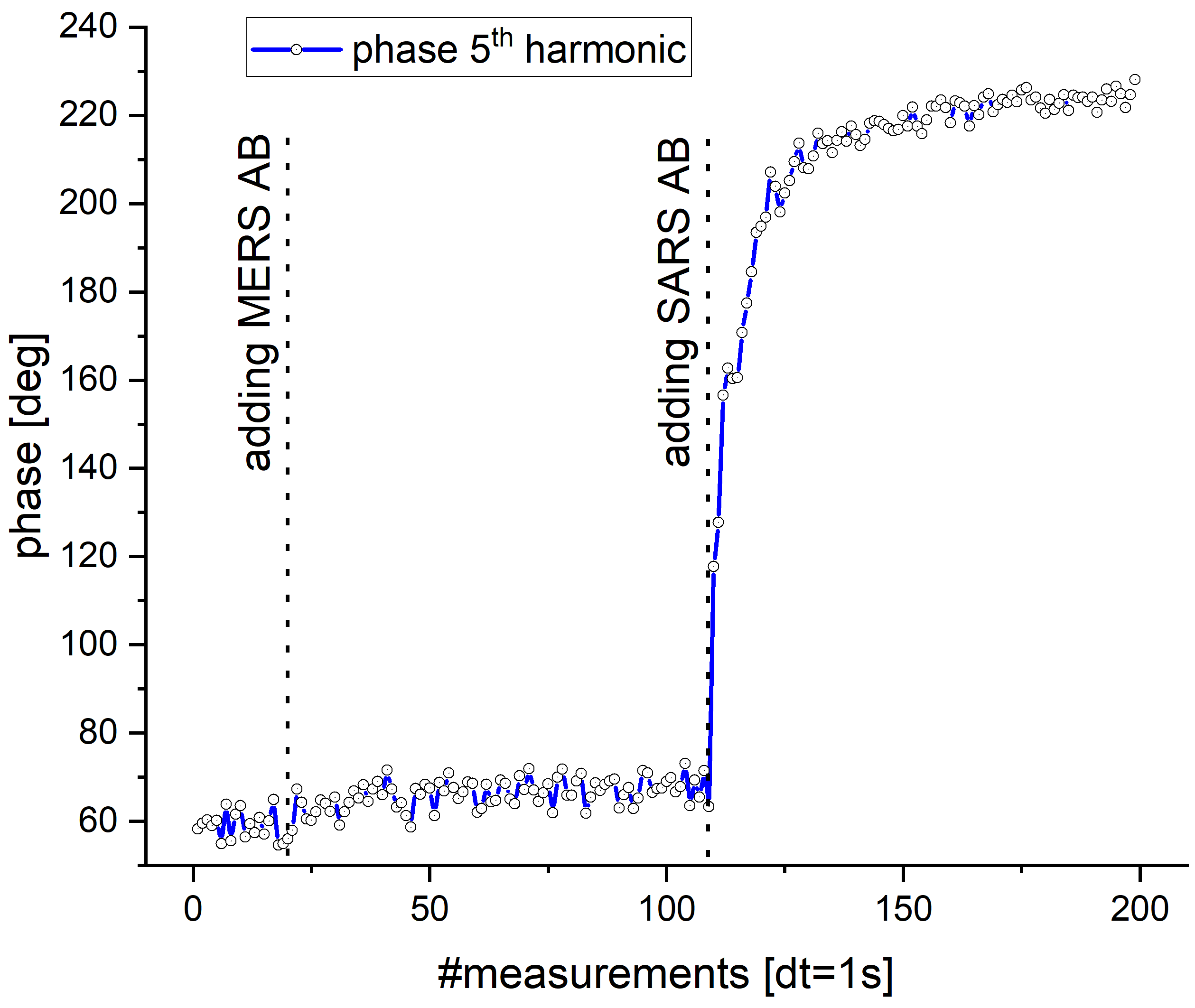


Supplementary figure 18. **Real-time antibody-antigen inter-action analysis using COMPASS**. A continuous phase measurement in time steps of 1 second show the binding dynamic of adding 25 µl MERS antibodies after 20 seconds and 25 µl SARS antibodies after 110 seconds to 25 µl APTES-MNP-SBA-S1 particle system.

**SUPPLEMENTARY REFERENCES**

1. Rahmer, J., Weizenecker, J., Gleich, B., Borgert, J. Signal encoding in magnetic particle imaging: properties of the system function. *BMC Med Imaging* **9(4)**, 19335923 (2009).
2. Coffey, W. T., Kalmykov, Y. P., Waldron, J. T. *The Langevin equation* 2nd ed. (World Scientific, Singapore, 2004)
3. Ota, S., Takemura, Y. Characterization of Néel and Brownian Relaxations Isolated from Complex Dynamics Influenced by Dipole Interactions in Magnetic Nanoparticles. *J Phys Chem* **123(47)**, 28859-28866 (2019).
4. Ilg, P., Kröger, M. Dynamics of interacting magnetic nanoparticles: effective behavior from competition between Brownian and Néel relaxation. *Chem Phys* **22**, 22244-22259 (2020).
5. Ludwig, F. & Remmer, H. Rotational dynamics of magnetic nanoparticles in different matrix systems. *Physical Sciences Reviews* **0190115** (2020).
6. Martens, M. A. et al. Modeling the Brownian relaxation of nanoparticle ferrofluids: Comparison with experiment. *Med Phys* **40(2)**, 022303 (2013).
7. Topping, C.V. & Blundell, S. J. A.C. susceptibility as a probe of low-frequency magnetic dynamics. *Condensed Matter* **31**, 013001 (2019).
8. McNaughton, B.H., Kehbein, K.A., Anker, J.N. & Kopelman, R. Sudden Breakdown in Linear Response of a Rotationally Driven Magnetic Microparticle and Application to Physical and Chemical Microsensing. *J Phys Chem* *B* **110(38)** 18958-18964 (2006).
9. Kahmann, T. & Ludwig, F. Magnetic field dependence of the effective magnetic moment of multi-core nanoparticles. *Journal of Appl Phys* **127(23)**, 233901 (2020).
10. Vogel P. Rückert, M. A., Kampf, T., Behr, V. C. Highly Flexible and Modular Simulation Framework for Magnetic Particle Imaging. Preprint at <https://doi.org/10.48550/arXiv.2208.13835> (2022).
11. Paulo, A.S. & Garcia, R. High-Resolution Imaging of Antibodies by Tapping-Mode Atomic Force Microscopy: Attractive and Repulsive Tip-Sample Interaction Regimes. *Biophysical Journal* **78**, 1599-1605 (2000).
12. Revets, H., De Baetselier, P., Muyldermans, S. Nanobodies as novel agents for cancer therapy. *Expert Opin Biol Ther* **5(1)**, 111-124 (2005).
13. Friedrich, B. et al. Scavenging of bacteria or bacterial products by magnetic particles functionalized with a broad-spectrum pathogen recognition receptor motif offers diagnostic and therapeutic applications. *Acta Biomater* **141**, 418-28 (2022).
14. Sjödahl, J. Structural Studies on the Four Repetitive Fc-Binding Regions in Protein A from Staphylococcus aureus. *Eur J Biochem* **78**, 471-490 (1977).
15. Biederer, S. et al. Magnetization response spectroscopy of superparamagnetic nanoparticles for magnetic particle imaging. *J Phys D* **42(20)** (2009).
16. Wu, K. et al. Magnetic Particle Spectroscopy: A short review of applications using magnetic nanoparticles. *ACS Appl Nano Mater* **3(6)**, 4972-4989 (2020).
17. Graeser, M. et al. Towards picogram detection of superparamagnetic iron-oxide particles using a gradiometric receive coil. *Sci Rep* **7**, 1-13 (2017).
18. Liebl, M. et al. Magnetic measurement methods to probe nanoparticle–matrix interactions, *Physical Sciences Reviews* **20190112** (2021).
19. Karlsson, R. Michaelsson, A., Mattsson, L. Kinetic analysis of monoclonal antibody-antigen interactions with a new biosensor based analytical system. *J Immunol Methods*, **145** 229-240 (1991).
20. Malmborg, A.C., Michaelsson, A., Ohlin, M., Jansson, B., Borrebaeck, C. A. Real Time Analysis of Antibody-Antigen Reaction Kinetics. *Scand J Immunol* **35**, 643-650 (1992).
21. Katsamba, P.S. et al. Kinetic analysis of a high-affinity antibody/antigen interaction performed by multiple Biacore users, *Anal Biochem* **352**, 208-221 (2006).
22. Armbruster, D.A., Pry, T. Limit of Blank, Limit of Detection and Limit of Quantification. *Clin Biochem Rev* **29(1)**, 49-52 (2008).
