# Supplementary material for: Critical Offset Magnetic PArticle SpectroScopy for rapid and highly sensitive medical point-of-care diagnostics
Source: Nat Commun. 2022 Nov 24;13:7230. doi: 10.1038/s41467-022-34941-y (PMC9700695; doi:10.1038/s41467-022-34941-y)
Supplement: Supplementary file 3 — Description of Additional Supplementary Files [file 41467_2022_34941_MOESM3_ESM.pdf]

Description of Additional Supplementary Files

File Name: Supplementary Movie 1
Description: **DC-field dependency of complex signal values for the 3rd harmonic.**
Top The movie shows the variation of the spectral signal distribution for an increasing offset field.
Bottom Complex signals and phase for increasing offset fields (DC). For the third harmonic one specific critical point (CP) is observable at the crossing point of the real and imaginary curves. At this point, the phase is highly sensitive to minimal changes in mobility of MNP ensembles.

File Name: Supplementary Movie 2
Description: **DC-field dependency of complex signal values for the 7th harmonic.**
Top The movie shows the variation of the spectral signal distribution for an increasing offset field.
Bottom Complex signals and phase for increasing offset fields (DC). For the seventh harmonic three specific critical points (CP) are observable at the crossing point of the real and imaginary curves.
