# Supplementary figures and images for: Critical Offset Magnetic PArticle SpectroScopy for rapid and highly sensitive medical point-of-care diagnostics (part 1 of 2)
Source: Nat Commun. 2022 Nov 24;13:7230. doi: 10.1038/s41467-022-34941-y (PMC9700695; doi:10.1038/s41467-022-34941-y)

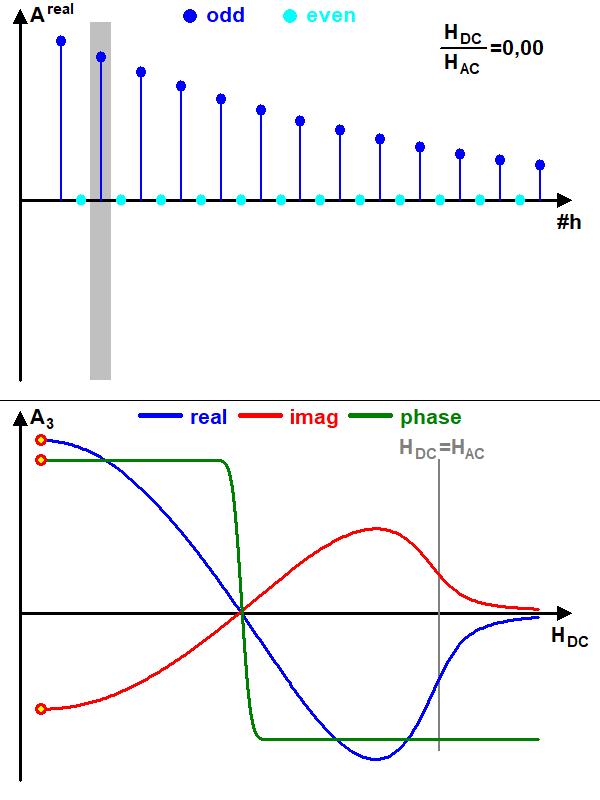

Supplement: Supplementary file 4 — Supplementary Movie 1 [file 41467_2022_34941_MOESM4_ESM.gif]

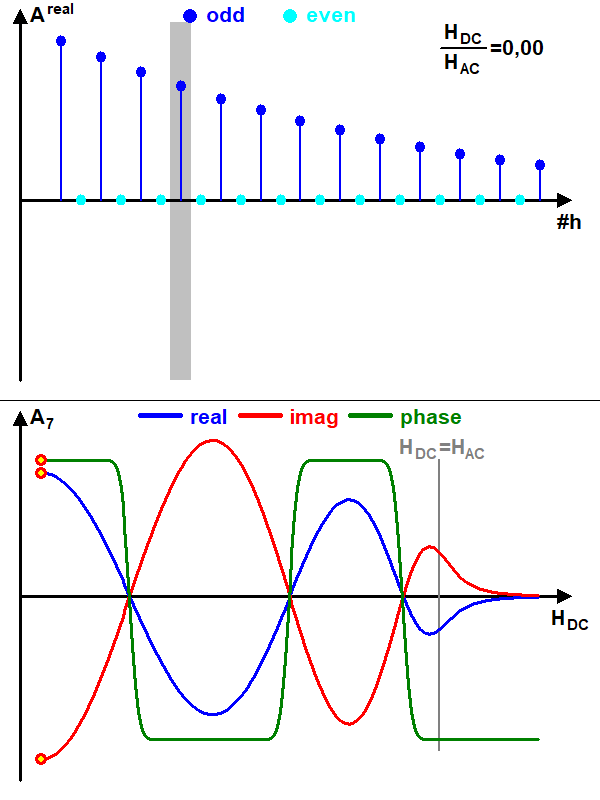

Supplement: Supplementary file 5 — Supplementary Movie 2 [file 41467_2022_34941_MOESM5_ESM.gif]

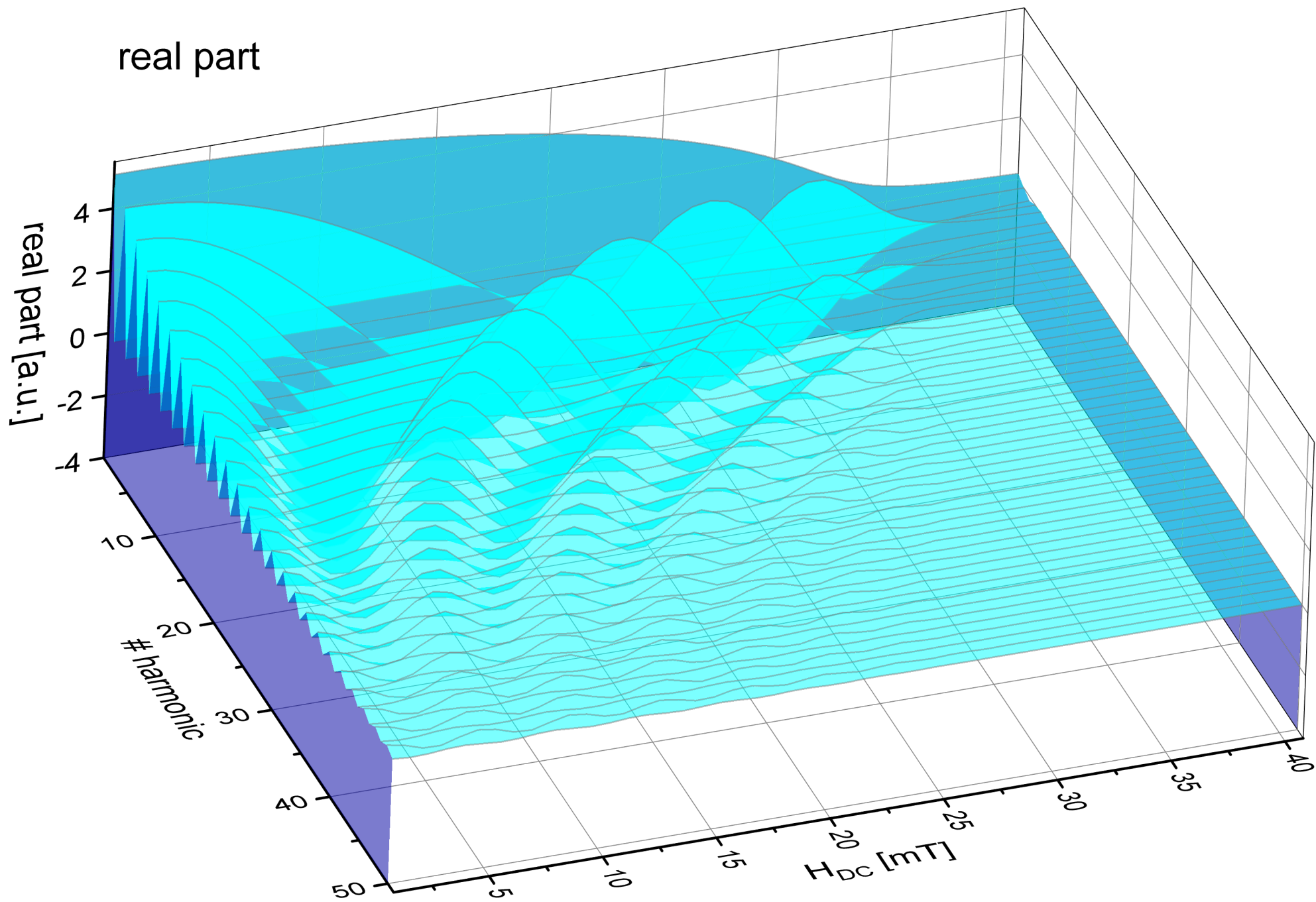

Supplement: Supplementary file 7 — Source Data [file 41467_2022_34941_MOESM7_ESM.zip › SI_fig01/SI_fig01_bottom.pdf]

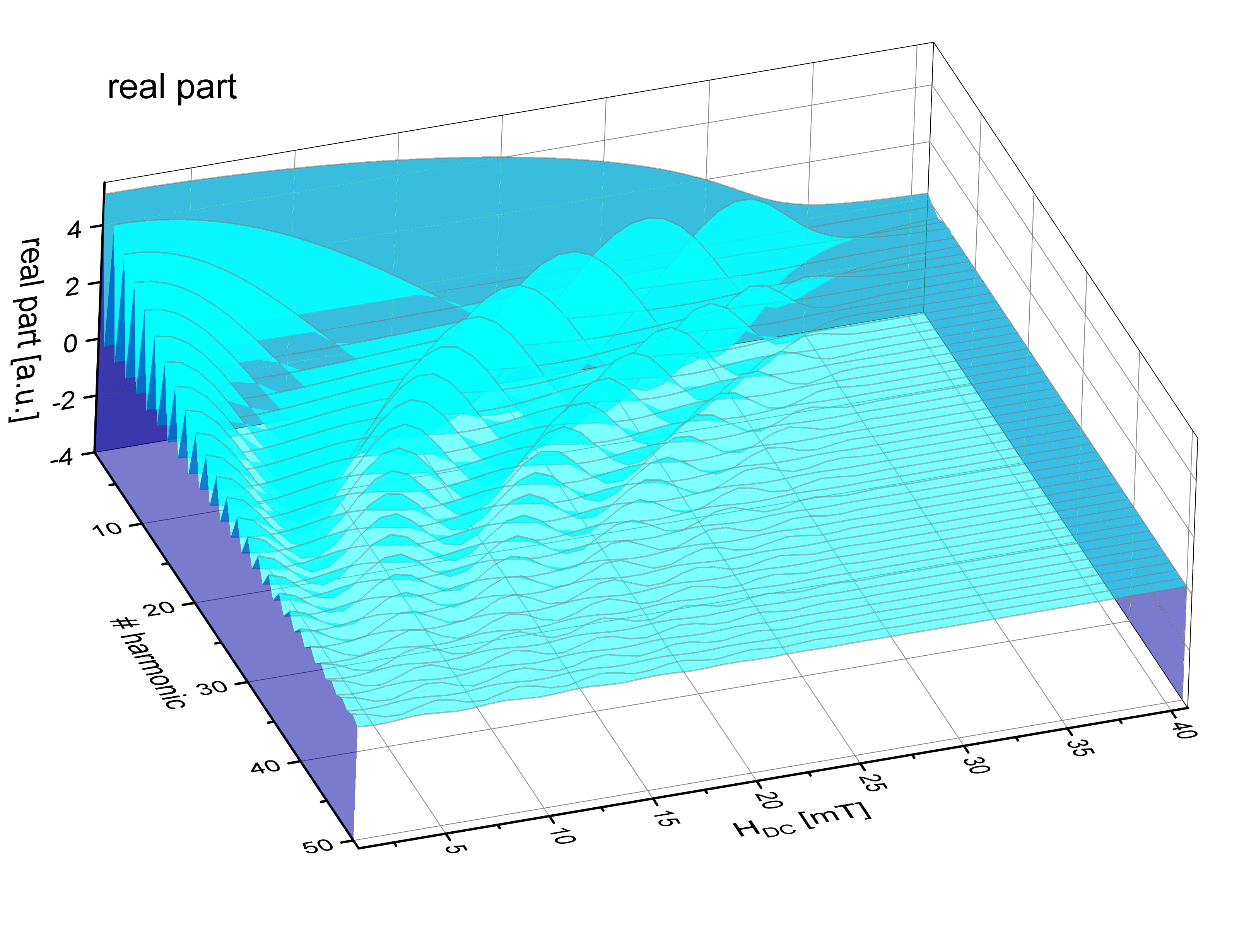

Supplement: Supplementary file 7 — Source Data [file 41467_2022_34941_MOESM7_ESM.zip › SI_fig01/SI_fig01_bottom.png]

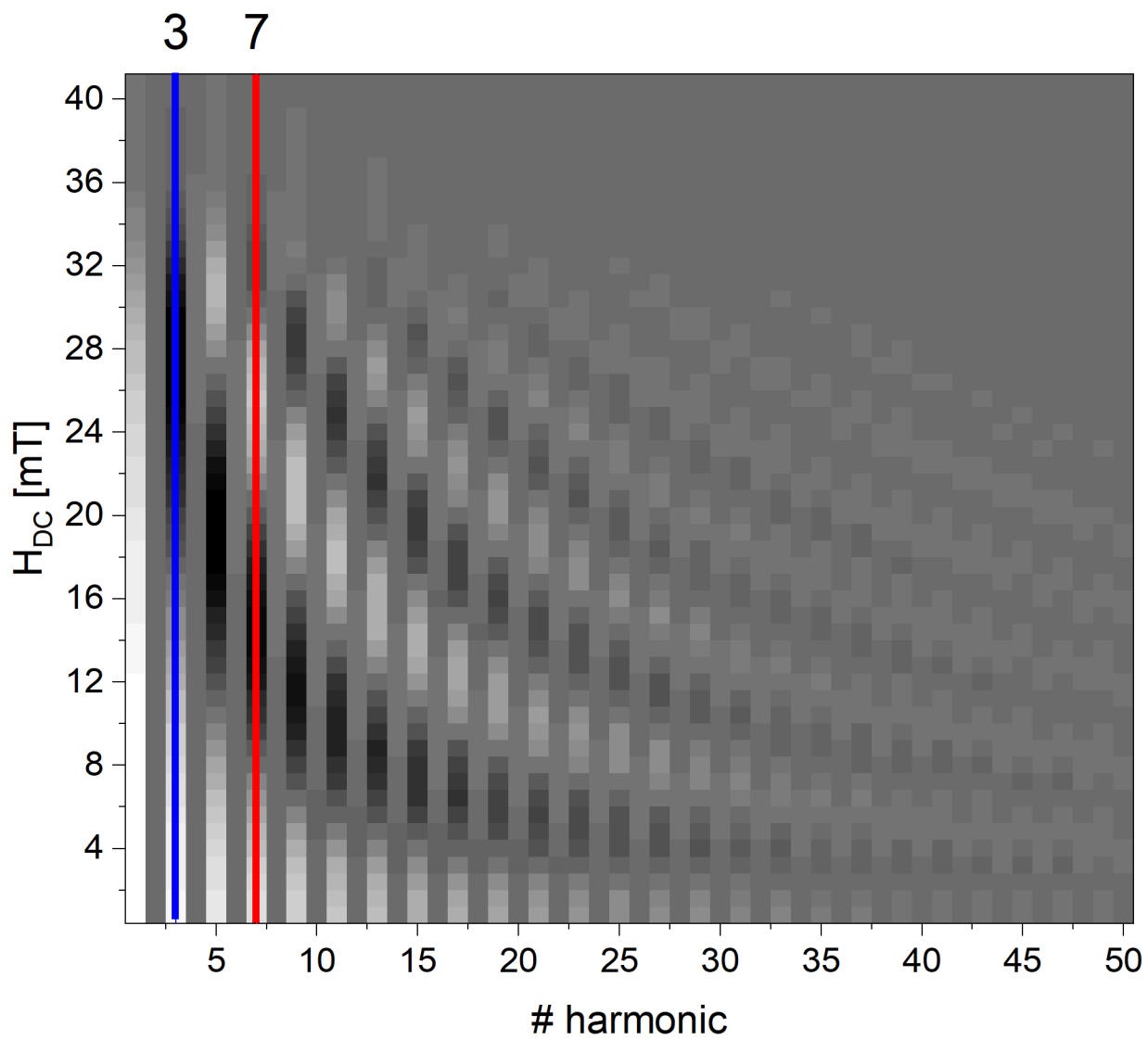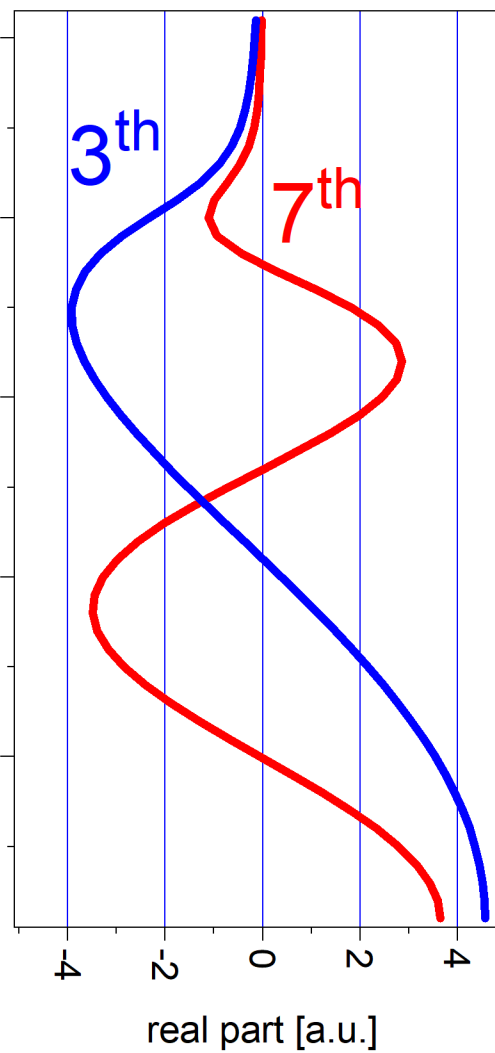

Supplement: Supplementary file 7 — Source Data [file 41467_2022_34941_MOESM7_ESM.zip › SI_fig01/SI_fig01_top.pdf]

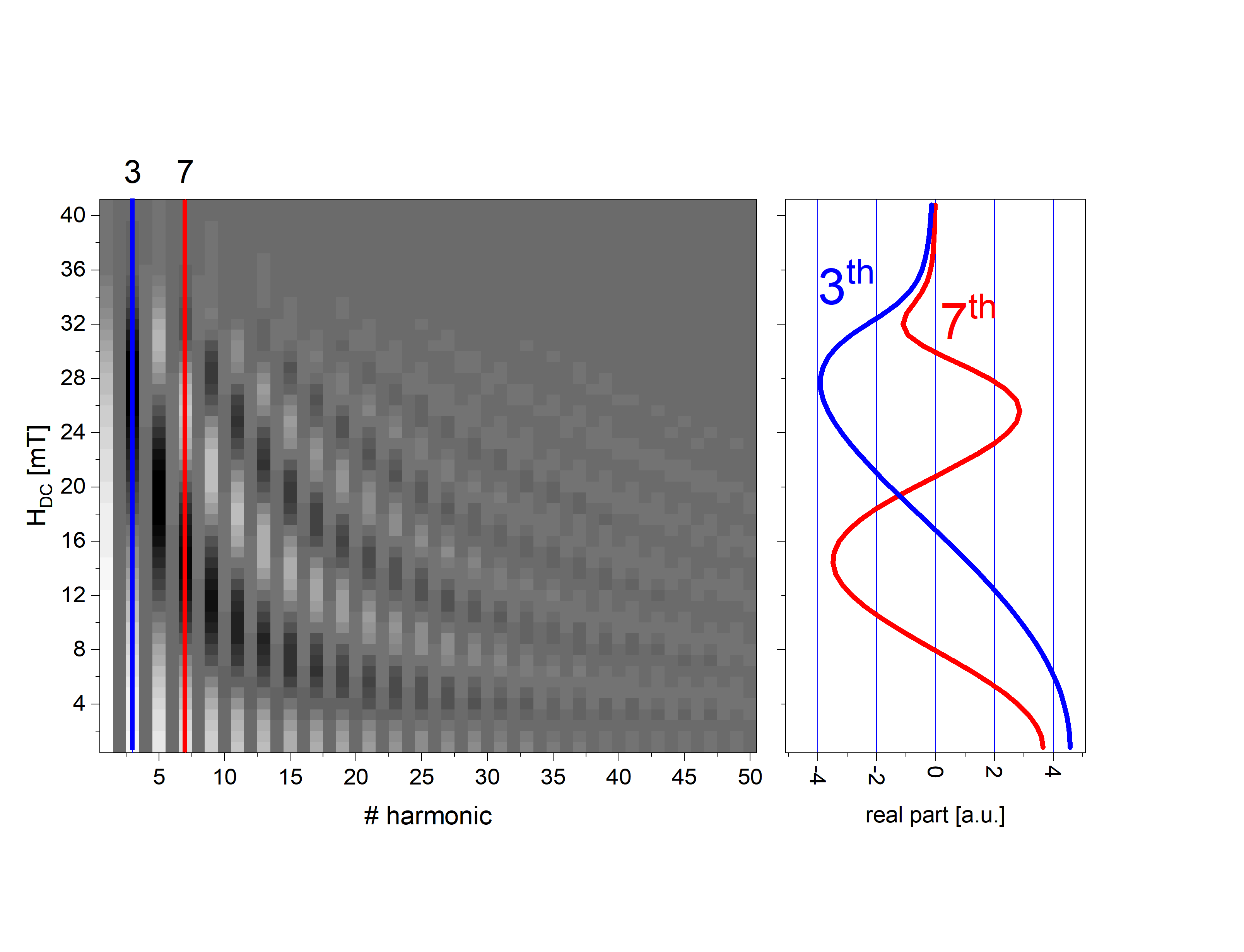

Supplement: Supplementary file 7 — Source Data [file 41467_2022_34941_MOESM7_ESM.zip › SI_fig01/SI_fig01_top.png]

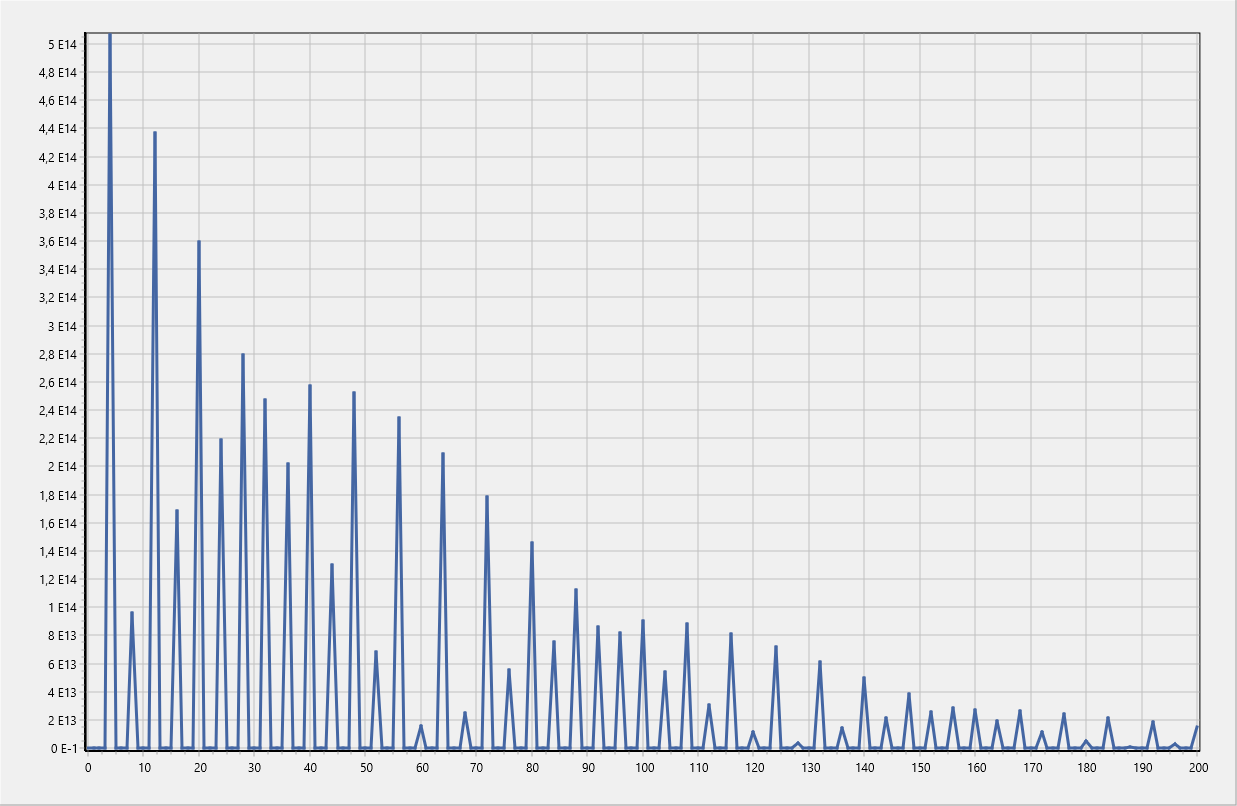

Supplement: Supplementary file 7 — Source Data [file 41467_2022_34941_MOESM7_ESM.zip › SI_fig02/interferenz01_all_abs.PNG]

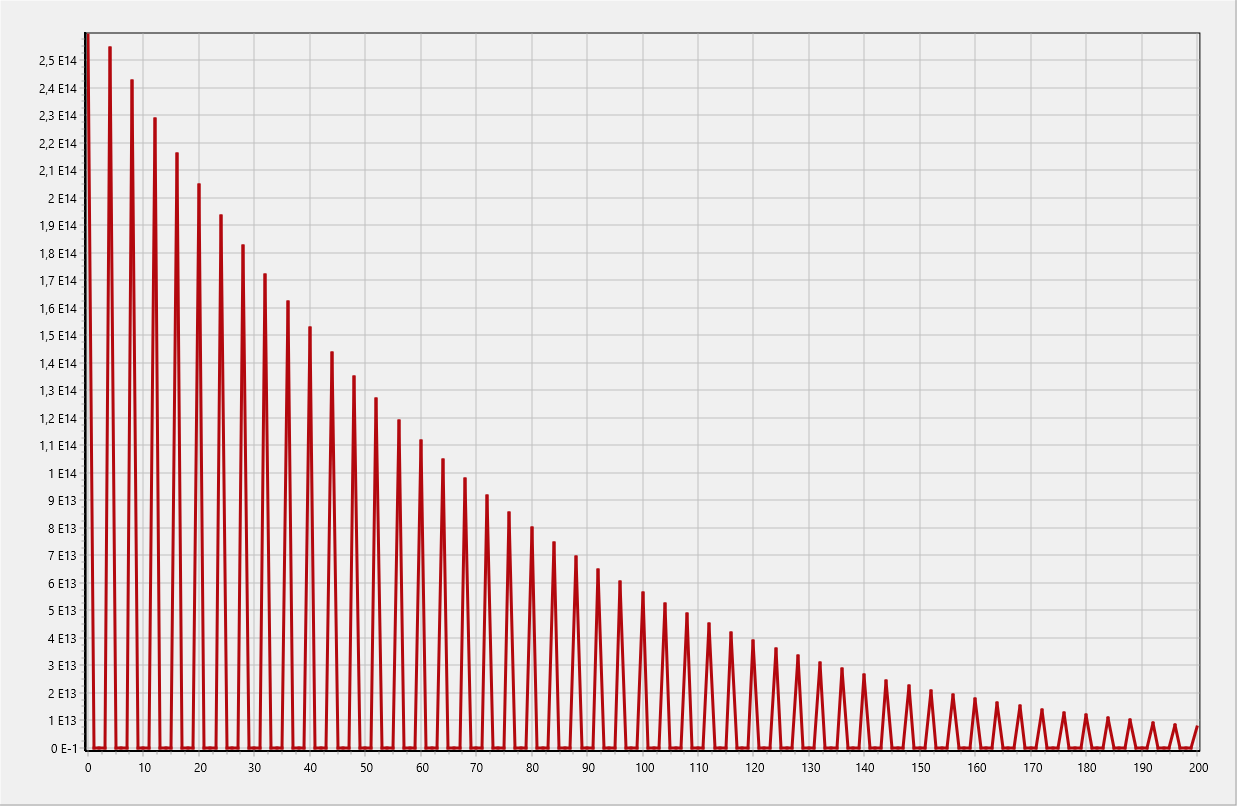

Supplement: Supplementary file 7 — Source Data [file 41467_2022_34941_MOESM7_ESM.zip › SI_fig02/interferenz01_neg_abs.PNG]

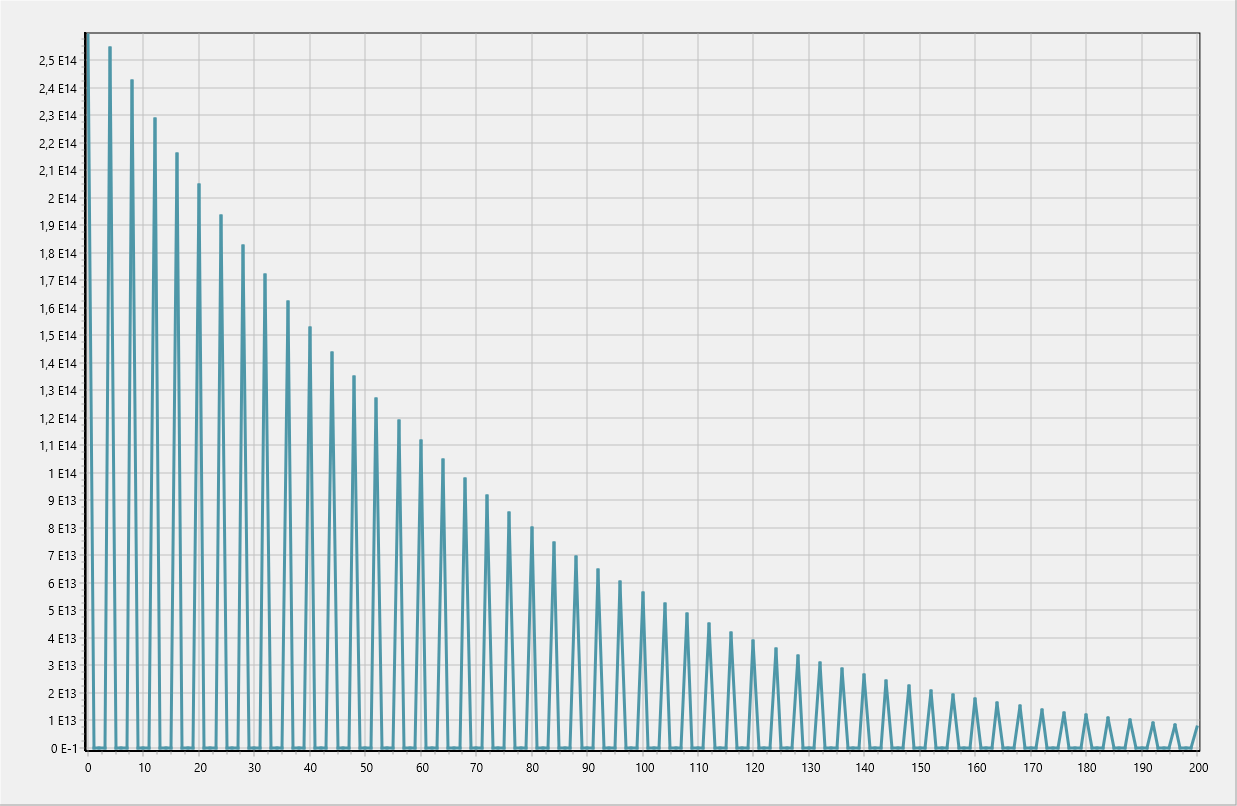

Supplement: Supplementary file 7 — Source Data [file 41467_2022_34941_MOESM7_ESM.zip › SI_fig02/interferenz01_pos_abs.PNG]

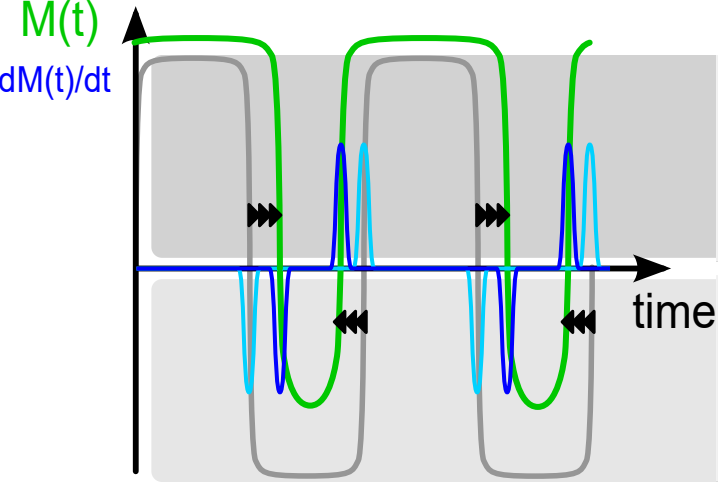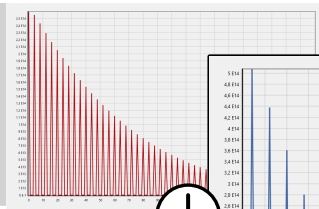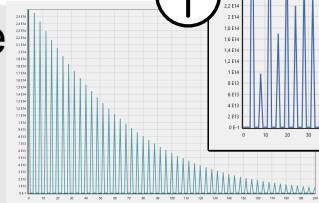

+

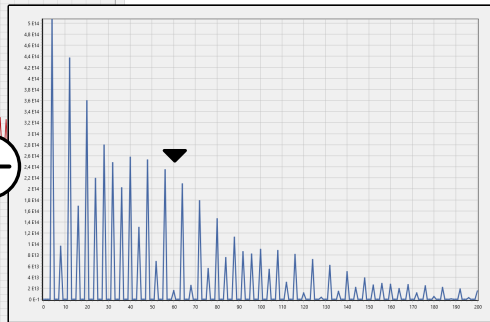

Supplement: Supplementary file 7 — Source Data [file 41467_2022_34941_MOESM7_ESM.zip › SI_fig02/SI_fig02.pdf]

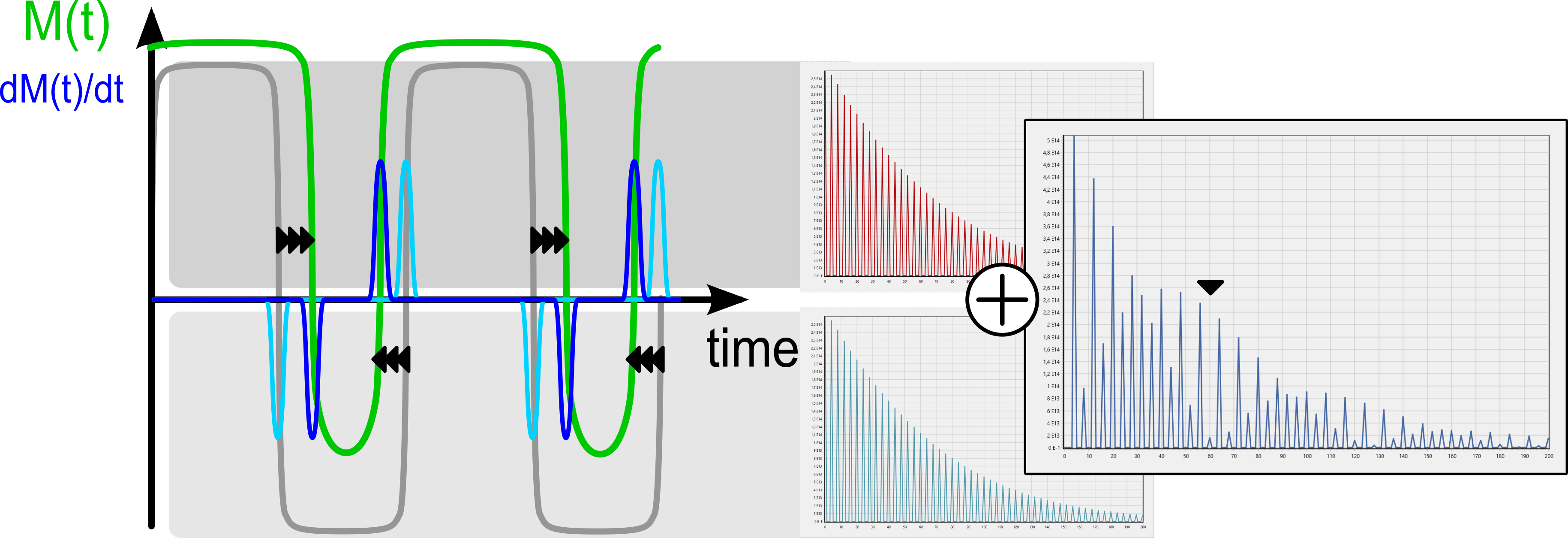

Supplement: Supplementary file 7 — Source Data [file 41467_2022_34941_MOESM7_ESM.zip › SI_fig02/SI_fig02.png]

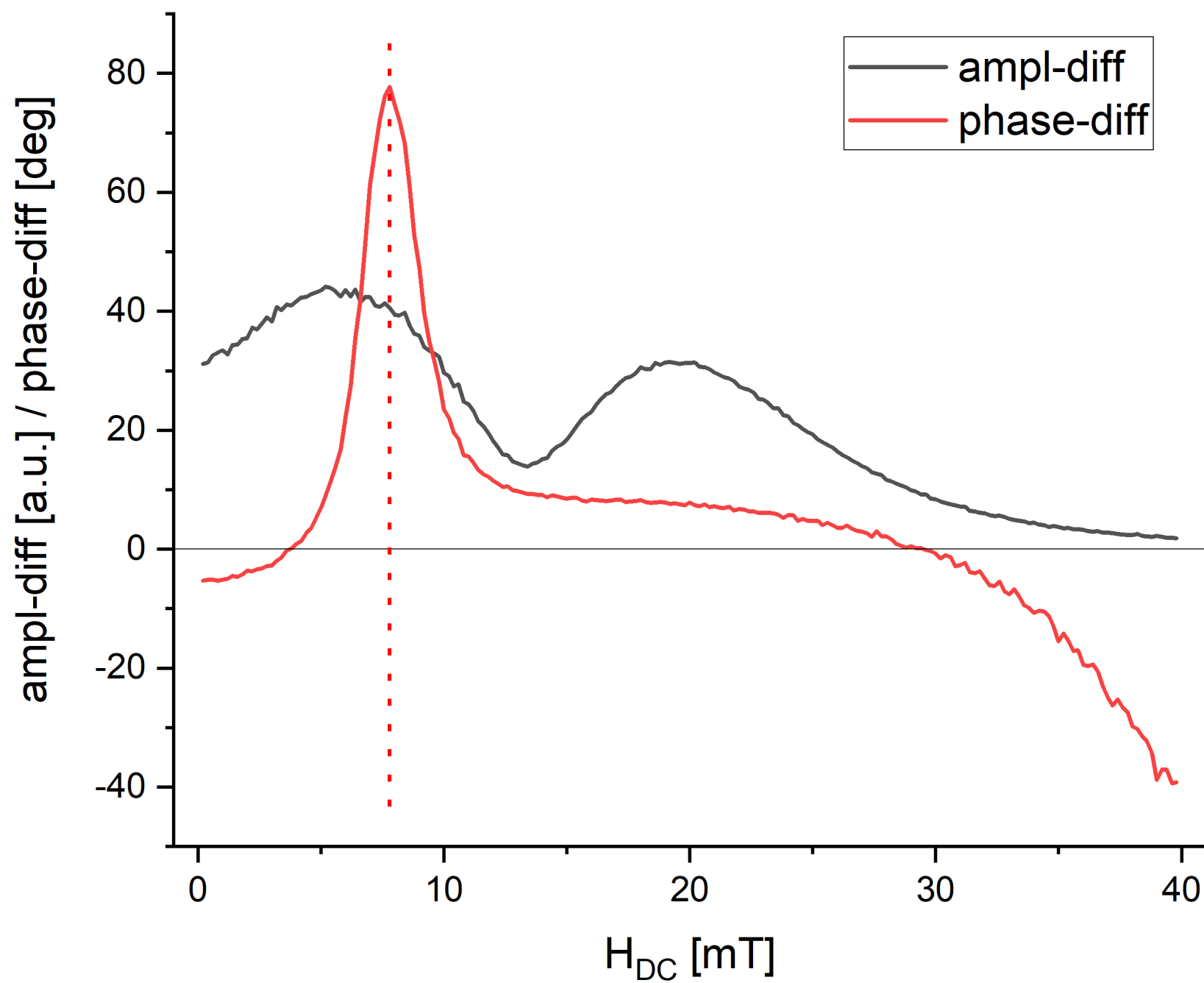

Supplement: Supplementary file 7 — Source Data [file 41467_2022_34941_MOESM7_ESM.zip › SI_fig03_fig04/SI_fig03_bottom.pdf]

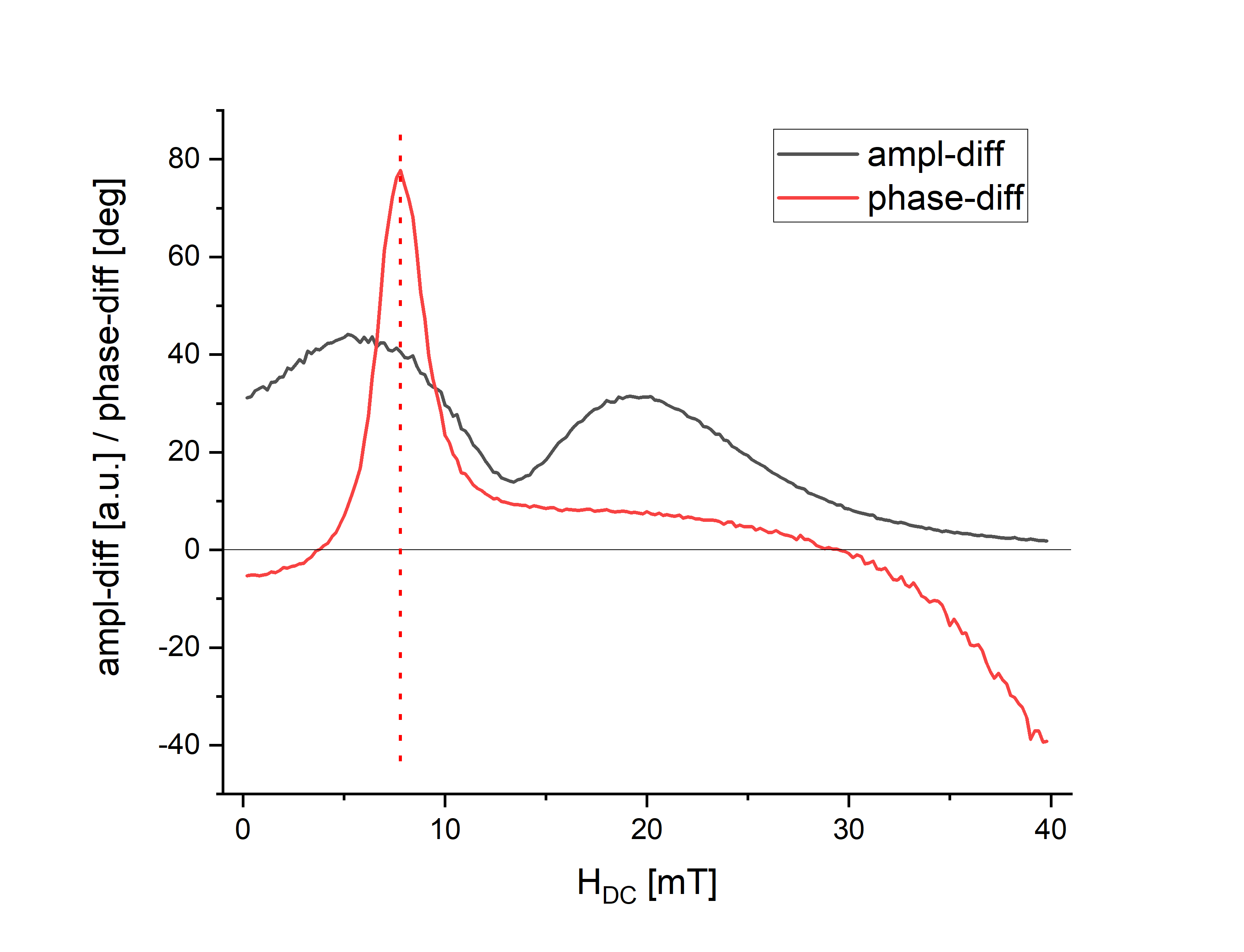

Supplement: Supplementary file 7 — Source Data [file 41467_2022_34941_MOESM7_ESM.zip › SI_fig03_fig04/SI_fig03_bottom.png]

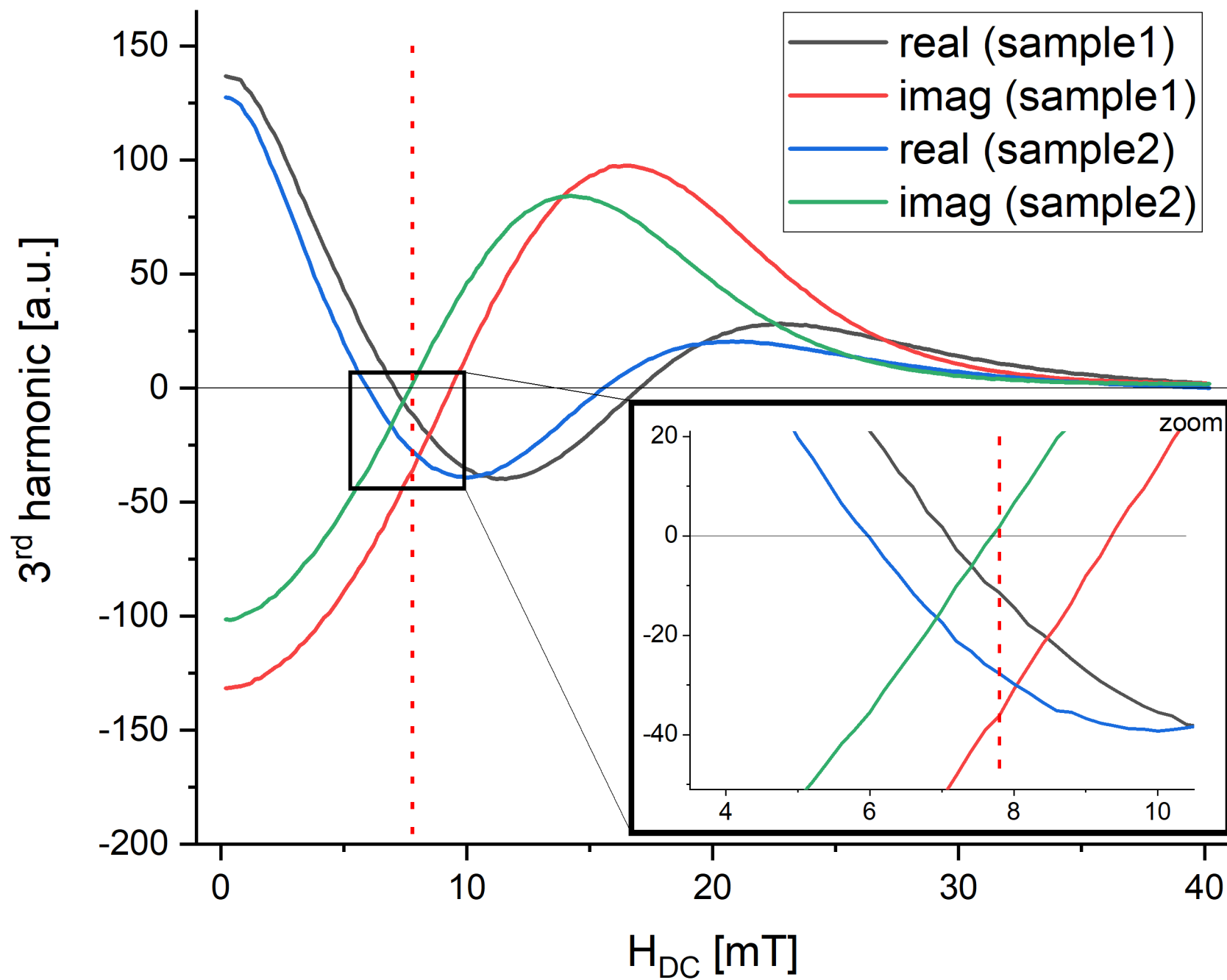

Supplement: Supplementary file 7 — Source Data [file 41467_2022_34941_MOESM7_ESM.zip › SI_fig03_fig04/SI_fig03_top.pdf]

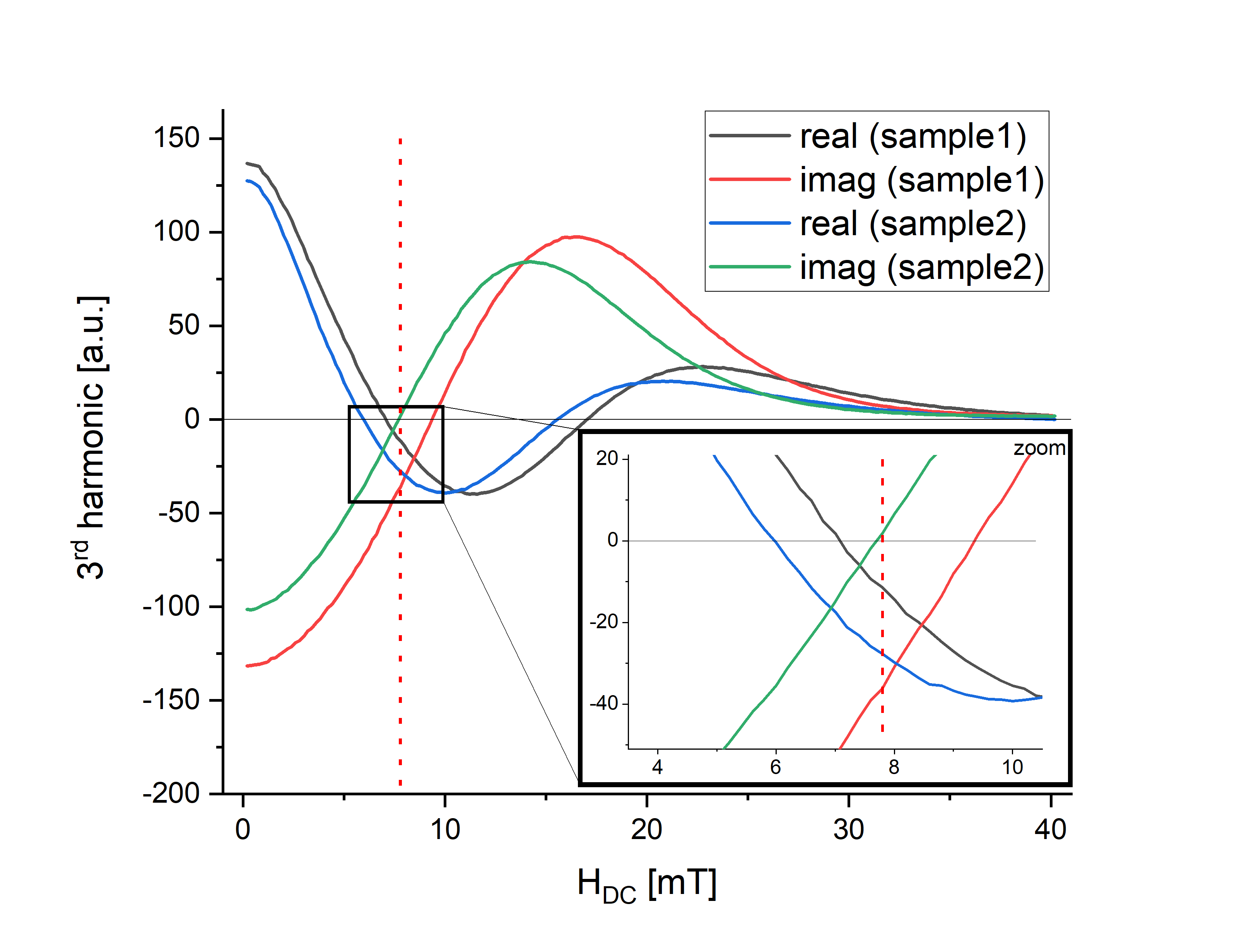

Supplement: Supplementary file 7 — Source Data [file 41467_2022_34941_MOESM7_ESM.zip › SI_fig03_fig04/SI_fig03_top.png]

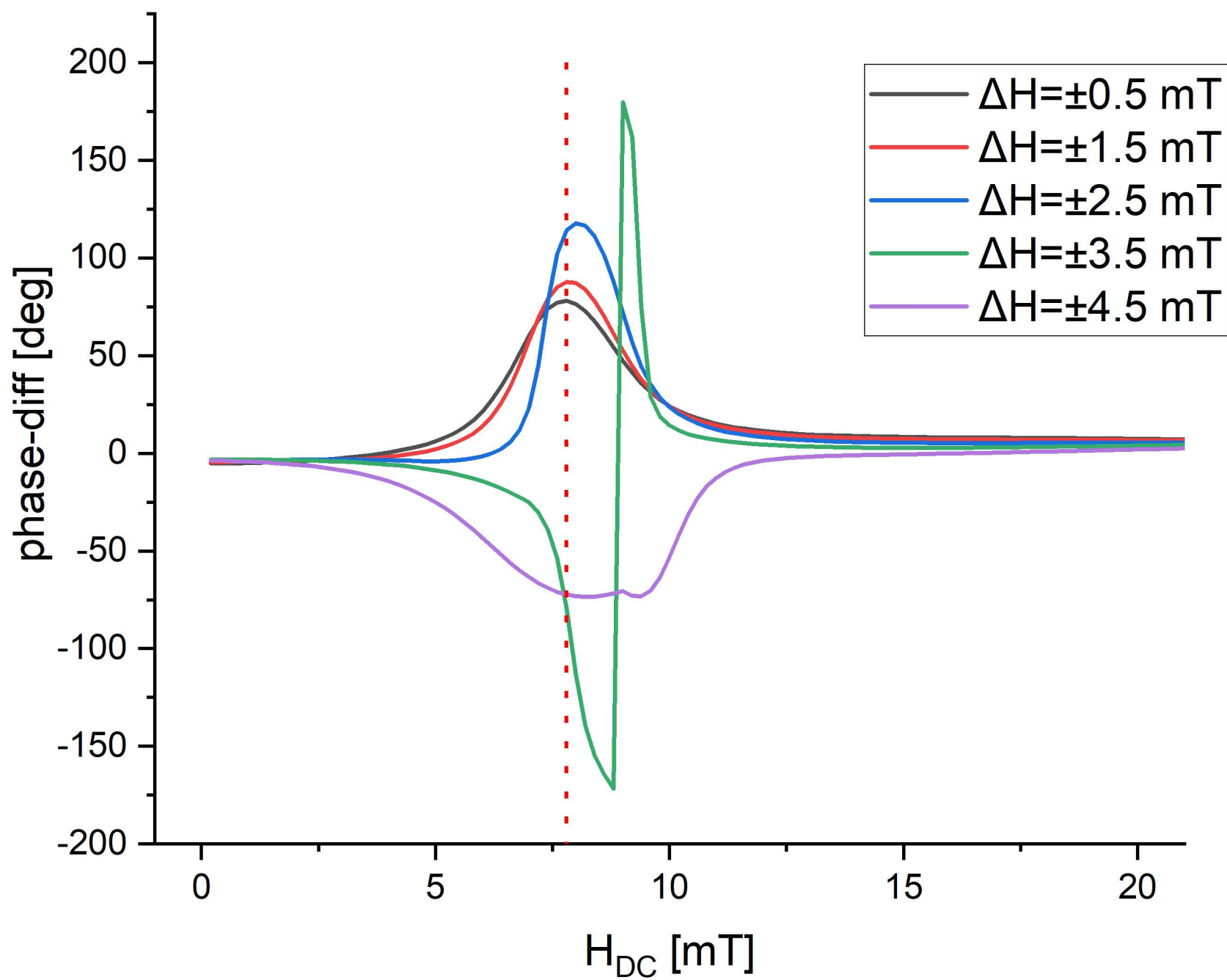

Supplement: Supplementary file 7 — Source Data [file 41467_2022_34941_MOESM7_ESM.zip › SI_fig03_fig04/SI_fig04.pdf]

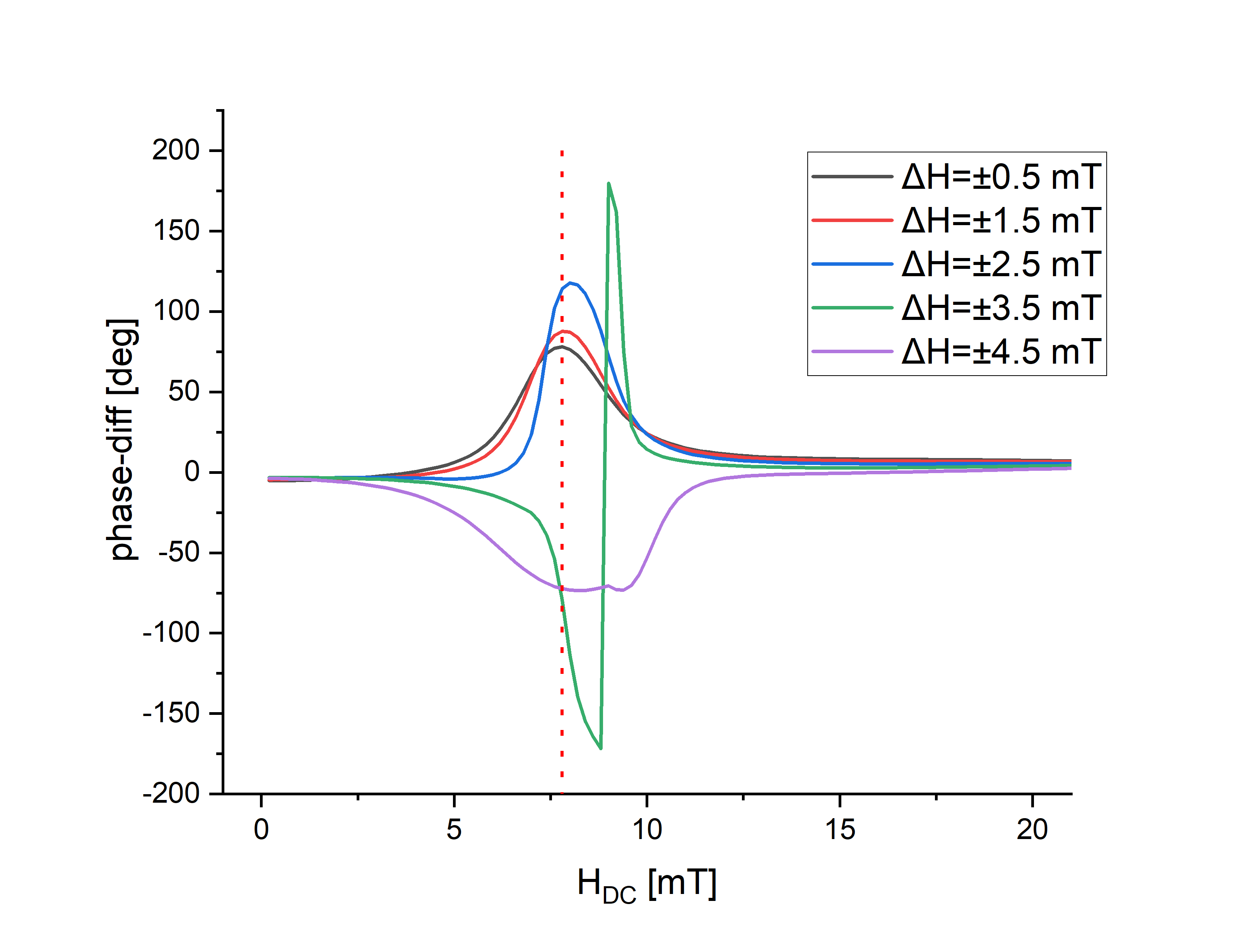

Supplement: Supplementary file 7 — Source Data [file 41467_2022_34941_MOESM7_ESM.zip › SI_fig03_fig04/SI_fig04.png]

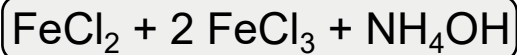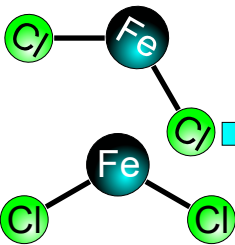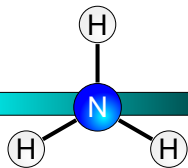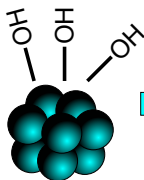

MNP

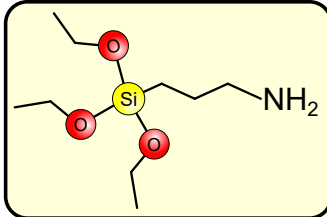

APTES

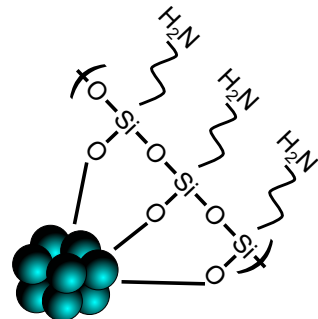

MNP-APTES

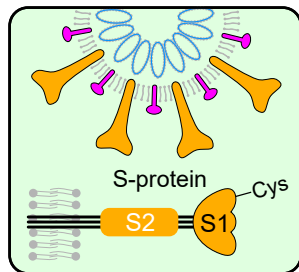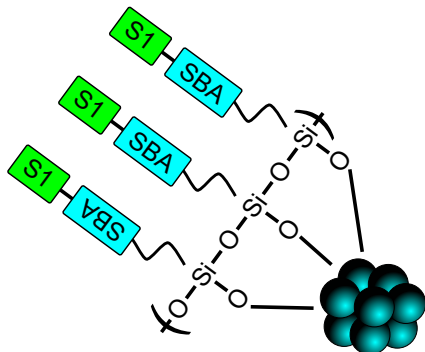

MNP-APTES-SBA-S1

SBA

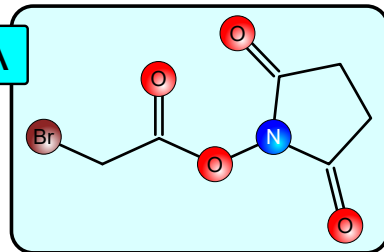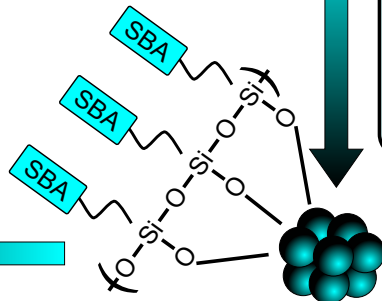

MNP-APTES-SBA

Supplement: Supplementary file 7 — Source Data [file 41467_2022_34941_MOESM7_ESM.zip › SI_fig05/SI_fig05.pdf]

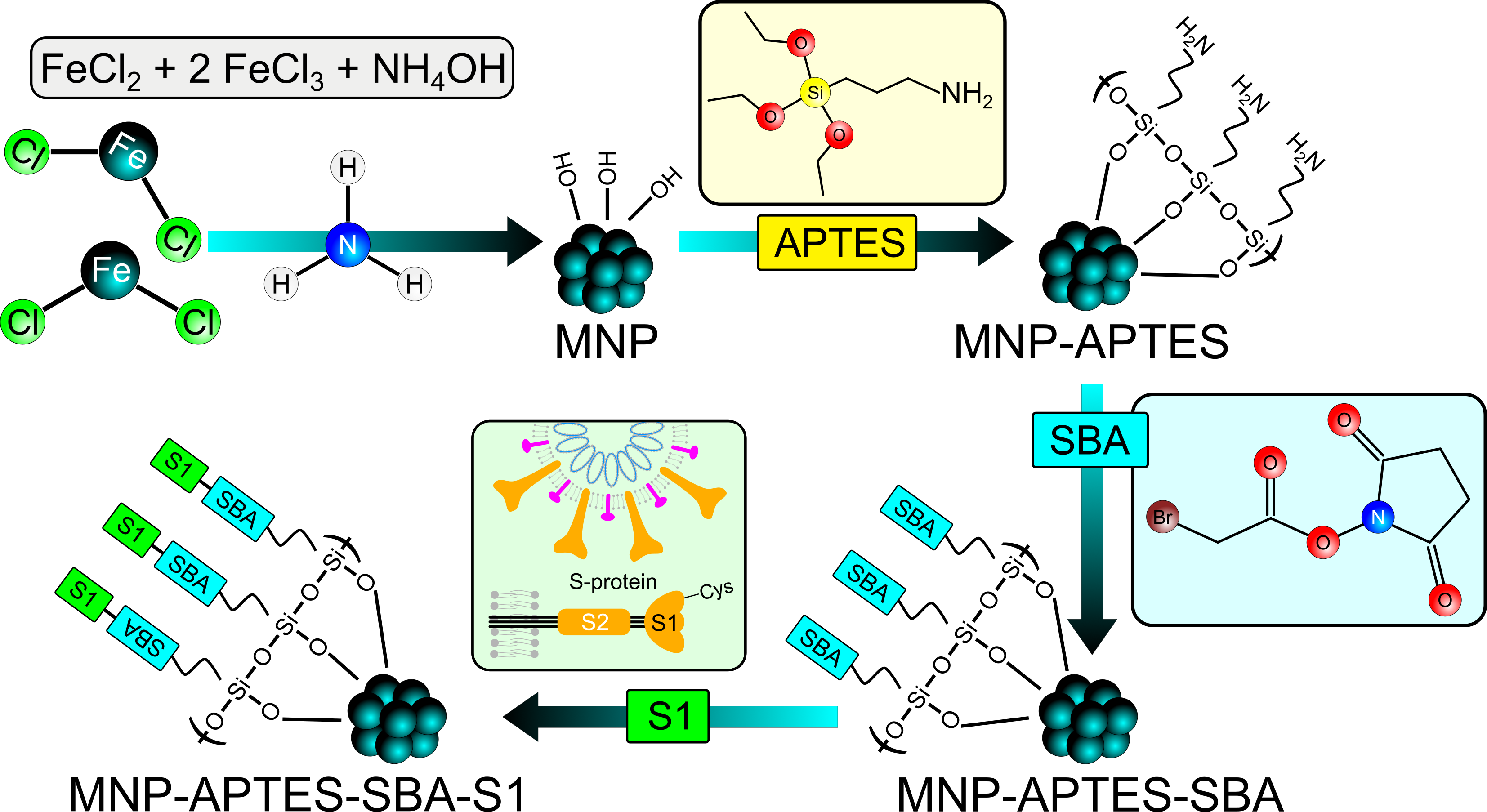

Supplement: Supplementary file 7 — Source Data [file 41467_2022_34941_MOESM7_ESM.zip › SI_fig05/SI_fig05.png]

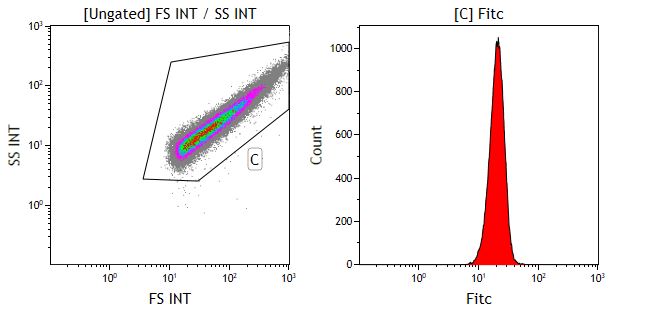

Supplement: Supplementary file 7 — Source Data [file 41467_2022_34941_MOESM7_ESM.zip › SI_fig06/SI_fig06.JPG]

[Ungated] FS INT / SS INT

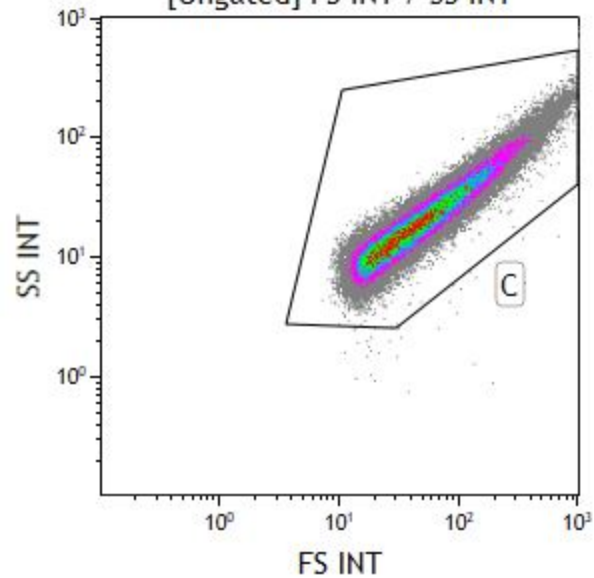

[C] Fitc

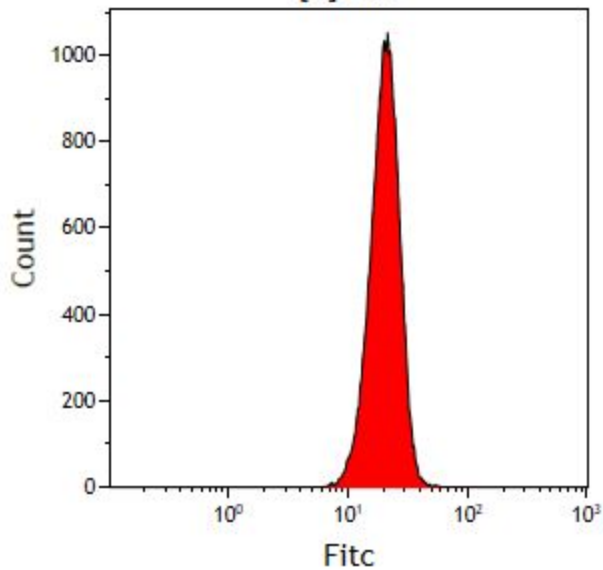

Supplement: Supplementary file 7 — Source Data [file 41467_2022_34941_MOESM7_ESM.zip › SI_fig06/SI_fig06.pdf]

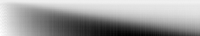

Supplement: Supplementary file 7 — Source Data [file 41467_2022_34941_MOESM7_ESM.zip › SI_fig07/02_imag.bmp]

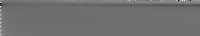

Supplement: Supplementary file 7 — Source Data [file 41467_2022_34941_MOESM7_ESM.zip › SI_fig07/02_phase.bmp]

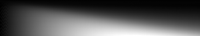

Supplement: Supplementary file 7 — Source Data [file 41467_2022_34941_MOESM7_ESM.zip › SI_fig07/02_real.bmp]

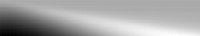

Supplement: Supplementary file 7 — Source Data [file 41467_2022_34941_MOESM7_ESM.zip › SI_fig07/03_imag.bmp]

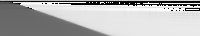

Supplement: Supplementary file 7 — Source Data [file 41467_2022_34941_MOESM7_ESM.zip › SI_fig07/03_phase.bmp]

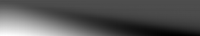

Supplement: Supplementary file 7 — Source Data [file 41467_2022_34941_MOESM7_ESM.zip › SI_fig07/03_real.bmp]

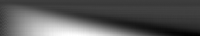

Supplement: Supplementary file 7 — Source Data [file 41467_2022_34941_MOESM7_ESM.zip › SI_fig07/04_imag.bmp]

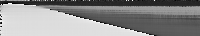

Supplement: Supplementary file 7 — Source Data [file 41467_2022_34941_MOESM7_ESM.zip › SI_fig07/04_phase.bmp]

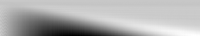

Supplement: Supplementary file 7 — Source Data [file 41467_2022_34941_MOESM7_ESM.zip › SI_fig07/04_real.bmp]

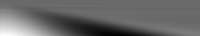

Supplement: Supplementary file 7 — Source Data [file 41467_2022_34941_MOESM7_ESM.zip › SI_fig07/05_imag.bmp]

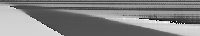

Supplement: Supplementary file 7 — Source Data [file 41467_2022_34941_MOESM7_ESM.zip › SI_fig07/05_phase.bmp]

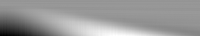

Supplement: Supplementary file 7 — Source Data [file 41467_2022_34941_MOESM7_ESM.zip › SI_fig07/05_real.bmp]

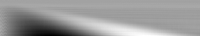

Supplement: Supplementary file 7 — Source Data [file 41467_2022_34941_MOESM7_ESM.zip › SI_fig07/06_imag.bmp]

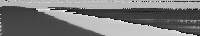

Supplement: Supplementary file 7 — Source Data [file 41467_2022_34941_MOESM7_ESM.zip › SI_fig07/06_phase.bmp]

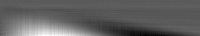

Supplement: Supplementary file 7 — Source Data [file 41467_2022_34941_MOESM7_ESM.zip › SI_fig07/06_real.bmp]

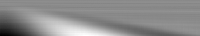

Supplement: Supplementary file 7 — Source Data [file 41467_2022_34941_MOESM7_ESM.zip › SI_fig07/07_imag.bmp]

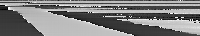

Supplement: Supplementary file 7 — Source Data [file 41467_2022_34941_MOESM7_ESM.zip › SI_fig07/07_phase.bmp]

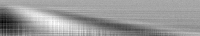

Supplement: Supplementary file 7 — Source Data [file 41467_2022_34941_MOESM7_ESM.zip › SI_fig07/07_real.bmp]

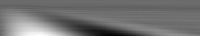

Supplement: Supplementary file 7 — Source Data [file 41467_2022_34941_MOESM7_ESM.zip › SI_fig07/08_imag.bmp]

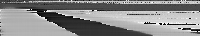

Supplement: Supplementary file 7 — Source Data [file 41467_2022_34941_MOESM7_ESM.zip › SI_fig07/08_phase.bmp]

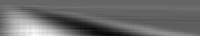

Supplement: Supplementary file 7 — Source Data [file 41467_2022_34941_MOESM7_ESM.zip › SI_fig07/08_real.bmp]

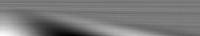

Supplement: Supplementary file 7 — Source Data [file 41467_2022_34941_MOESM7_ESM.zip › SI_fig07/09_imag.bmp]

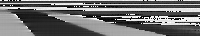

Supplement: Supplementary file 7 — Source Data [file 41467_2022_34941_MOESM7_ESM.zip › SI_fig07/09_phase.bmp]

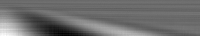

Supplement: Supplementary file 7 — Source Data [file 41467_2022_34941_MOESM7_ESM.zip › SI_fig07/09_real.bmp]

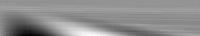

Supplement: Supplementary file 7 — Source Data [file 41467_2022_34941_MOESM7_ESM.zip › SI_fig07/10_imag.bmp]

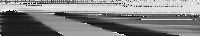

Supplement: Supplementary file 7 — Source Data [file 41467_2022_34941_MOESM7_ESM.zip › SI_fig07/10_phase.bmp]

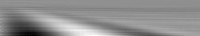

Supplement: Supplementary file 7 — Source Data [file 41467_2022_34941_MOESM7_ESM.zip › SI_fig07/10_real.bmp]

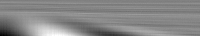

Supplement: Supplementary file 7 — Source Data [file 41467_2022_34941_MOESM7_ESM.zip › SI_fig07/11_imag.bmp]

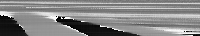

Supplement: Supplementary file 7 — Source Data [file 41467_2022_34941_MOESM7_ESM.zip › SI_fig07/11_phase.bmp]

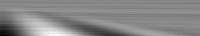

Supplement: Supplementary file 7 — Source Data [file 41467_2022_34941_MOESM7_ESM.zip › SI_fig07/11_real.bmp]

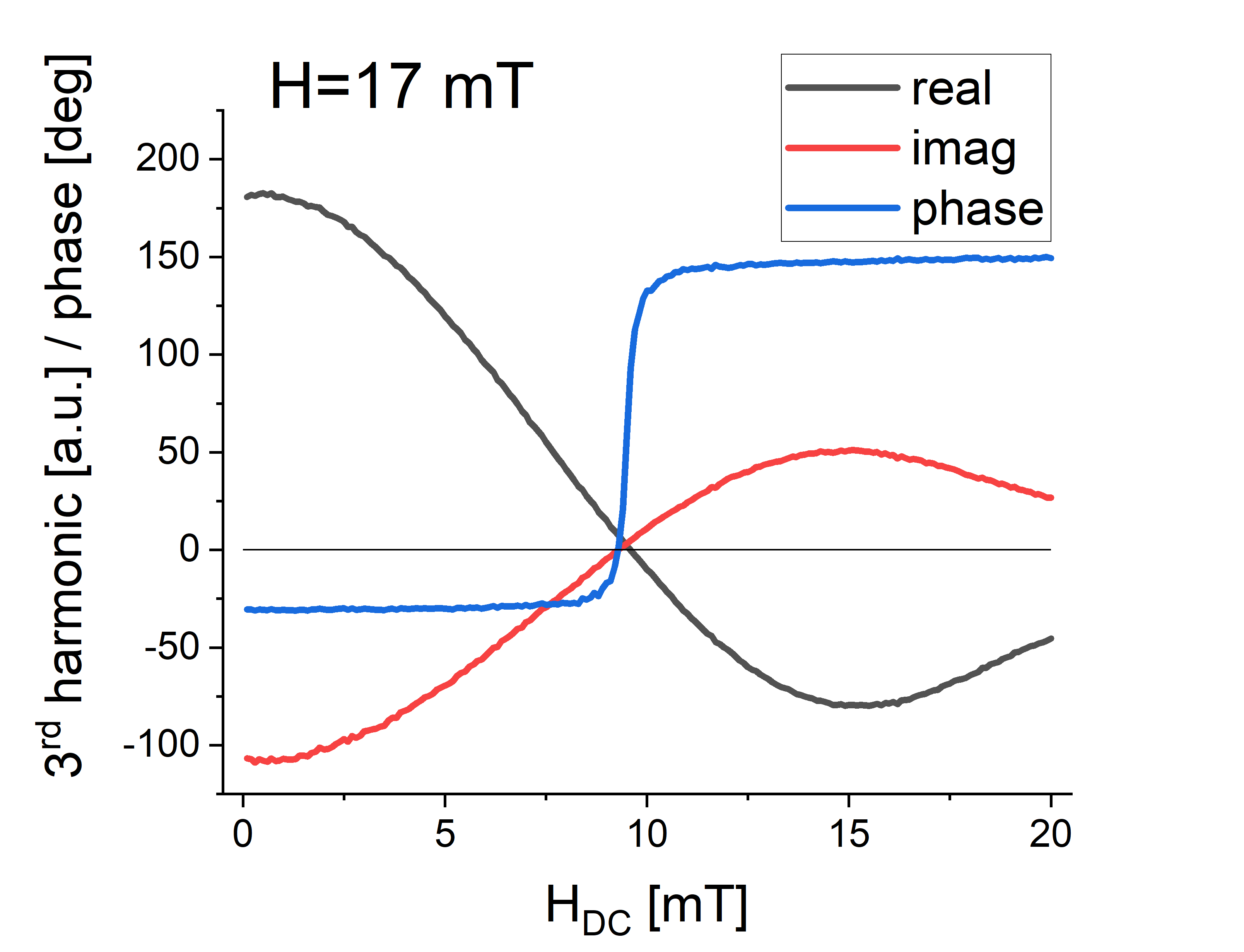

Supplement: Supplementary file 7 — Source Data [file 41467_2022_34941_MOESM7_ESM.zip › SI_fig07/Graph2.png]

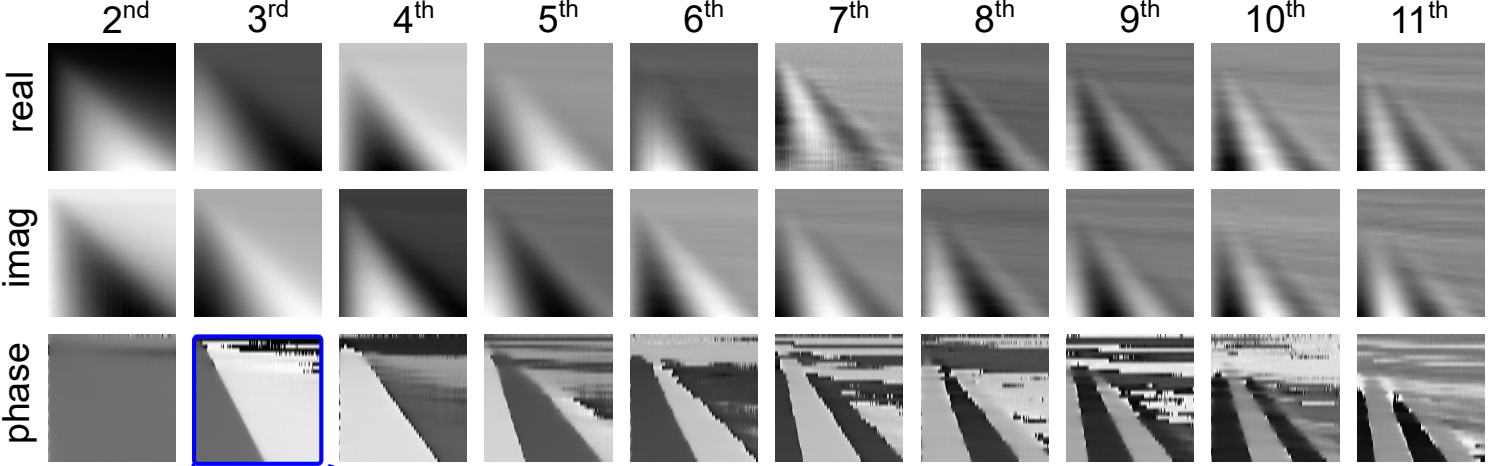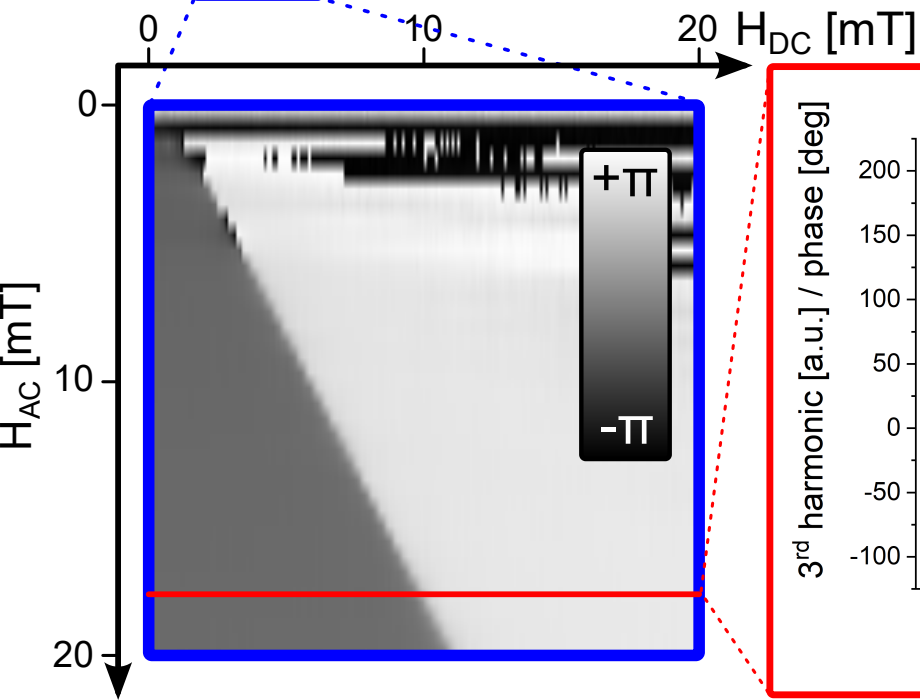

3<sup>rd</sup> harmonic [a.u.] / phase [deg]

$H_{AC}=17\text{mT}$

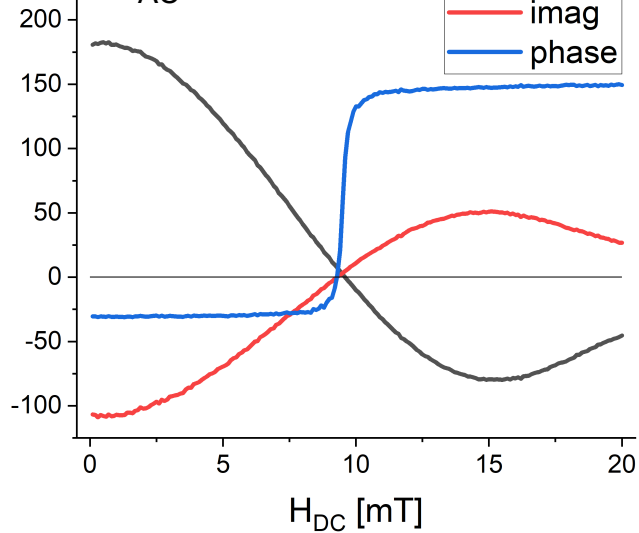

Supplement: Supplementary file 7 — Source Data [file 41467_2022_34941_MOESM7_ESM.zip › SI_fig07/SI_fig07.pdf]

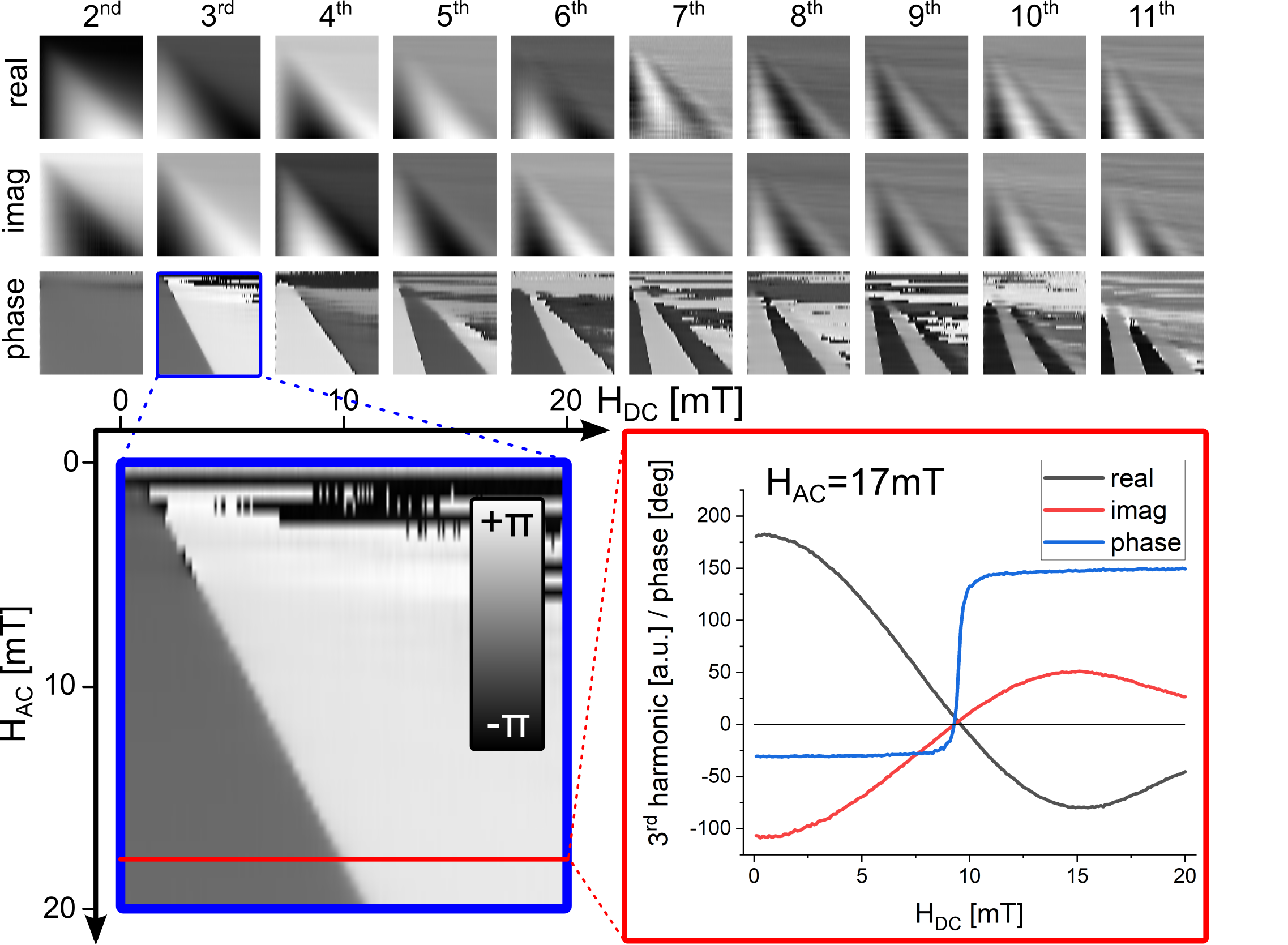

Supplement: Supplementary file 7 — Source Data [file 41467_2022_34941_MOESM7_ESM.zip › SI_fig07/SI_fig07.png]

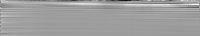

Supplement: Supplementary file 7 — Source Data [file 41467_2022_34941_MOESM7_ESM.zip › SI_fig08_fig09/51_1_sub_51_2__02_sumphase10.bmp]

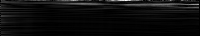

Supplement: Supplementary file 7 — Source Data [file 41467_2022_34941_MOESM7_ESM.zip › SI_fig08_fig09/51_1_sub_51_2__02_sumphase10_abs.bmp]

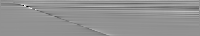

Supplement: Supplementary file 7 — Source Data [file 41467_2022_34941_MOESM7_ESM.zip › SI_fig08_fig09/51_1_sub_51_2__03_sumphase10.bmp]

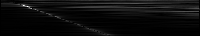

Supplement: Supplementary file 7 — Source Data [file 41467_2022_34941_MOESM7_ESM.zip › SI_fig08_fig09/51_1_sub_51_2__03_sumphase10_abs.bmp]

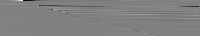

Supplement: Supplementary file 7 — Source Data [file 41467_2022_34941_MOESM7_ESM.zip › SI_fig08_fig09/51_1_sub_51_2__04_sumphase10.bmp]

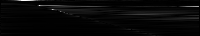

Supplement: Supplementary file 7 — Source Data [file 41467_2022_34941_MOESM7_ESM.zip › SI_fig08_fig09/51_1_sub_51_2__04_sumphase10_abs.bmp]

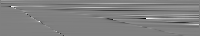

Supplement: Supplementary file 7 — Source Data [file 41467_2022_34941_MOESM7_ESM.zip › SI_fig08_fig09/51_1_sub_51_2__05_sumphase10.bmp]

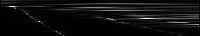

Supplement: Supplementary file 7 — Source Data [file 41467_2022_34941_MOESM7_ESM.zip › SI_fig08_fig09/51_1_sub_51_2__05_sumphase10_abs.bmp]

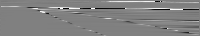

Supplement: Supplementary file 7 — Source Data [file 41467_2022_34941_MOESM7_ESM.zip › SI_fig08_fig09/51_1_sub_51_2__06_sumphase10.bmp]

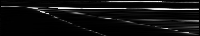

Supplement: Supplementary file 7 — Source Data [file 41467_2022_34941_MOESM7_ESM.zip › SI_fig08_fig09/51_1_sub_51_2__06_sumphase10_abs.bmp]

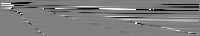

Supplement: Supplementary file 7 — Source Data [file 41467_2022_34941_MOESM7_ESM.zip › SI_fig08_fig09/51_1_sub_51_2__07_sumphase10.bmp]

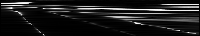

Supplement: Supplementary file 7 — Source Data [file 41467_2022_34941_MOESM7_ESM.zip › SI_fig08_fig09/51_1_sub_51_2__07_sumphase10_abs.bmp]

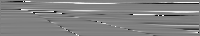

Supplement: Supplementary file 7 — Source Data [file 41467_2022_34941_MOESM7_ESM.zip › SI_fig08_fig09/51_1_sub_51_2__08_sumphase10.bmp]

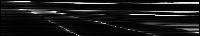

Supplement: Supplementary file 7 — Source Data [file 41467_2022_34941_MOESM7_ESM.zip › SI_fig08_fig09/51_1_sub_51_2__08_sumphase10_abs.bmp]

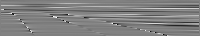

Supplement: Supplementary file 7 — Source Data [file 41467_2022_34941_MOESM7_ESM.zip › SI_fig08_fig09/51_1_sub_51_2__09_sumphase10.bmp]

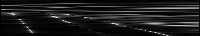

Supplement: Supplementary file 7 — Source Data [file 41467_2022_34941_MOESM7_ESM.zip › SI_fig08_fig09/51_1_sub_51_2__09_sumphase10_abs.bmp]

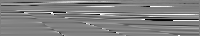

Supplement: Supplementary file 7 — Source Data [file 41467_2022_34941_MOESM7_ESM.zip › SI_fig08_fig09/51_1_sub_51_2__10_sumphase10.bmp]

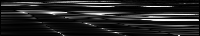

Supplement: Supplementary file 7 — Source Data [file 41467_2022_34941_MOESM7_ESM.zip › SI_fig08_fig09/51_1_sub_51_2__10_sumphase10_abs.bmp]

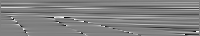

Supplement: Supplementary file 7 — Source Data [file 41467_2022_34941_MOESM7_ESM.zip › SI_fig08_fig09/51_1_sub_51_2__11_sumphase10.bmp]

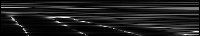

Supplement: Supplementary file 7 — Source Data [file 41467_2022_34941_MOESM7_ESM.zip › SI_fig08_fig09/51_1_sub_51_2__11_sumphase10_abs.bmp]

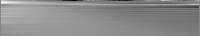

Supplement: Supplementary file 7 — Source Data [file 41467_2022_34941_MOESM7_ESM.zip › SI_fig08_fig09/51_1_sub_52_1__02_sumphase10.bmp]

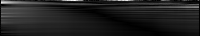

Supplement: Supplementary file 7 — Source Data [file 41467_2022_34941_MOESM7_ESM.zip › SI_fig08_fig09/51_1_sub_52_1__02_sumphase10_abs.bmp]

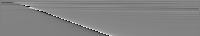

Supplement: Supplementary file 7 — Source Data [file 41467_2022_34941_MOESM7_ESM.zip › SI_fig08_fig09/51_1_sub_52_1__03_sumphase10.bmp]

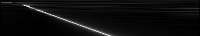

Supplement: Supplementary file 7 — Source Data [file 41467_2022_34941_MOESM7_ESM.zip › SI_fig08_fig09/51_1_sub_52_1__03_sumphase10_abs.bmp]

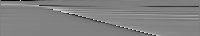

Supplement: Supplementary file 7 — Source Data [file 41467_2022_34941_MOESM7_ESM.zip › SI_fig08_fig09/51_1_sub_52_1__04_sumphase10.bmp]

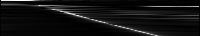

Supplement: Supplementary file 7 — Source Data [file 41467_2022_34941_MOESM7_ESM.zip › SI_fig08_fig09/51_1_sub_52_1__04_sumphase10_abs.bmp]

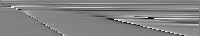

Supplement: Supplementary file 7 — Source Data [file 41467_2022_34941_MOESM7_ESM.zip › SI_fig08_fig09/51_1_sub_52_1__05_sumphase10.bmp]

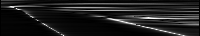

Supplement: Supplementary file 7 — Source Data [file 41467_2022_34941_MOESM7_ESM.zip › SI_fig08_fig09/51_1_sub_52_1__05_sumphase10_abs.bmp]

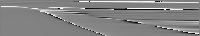

Supplement: Supplementary file 7 — Source Data [file 41467_2022_34941_MOESM7_ESM.zip › SI_fig08_fig09/51_1_sub_52_1__06_sumphase10.bmp]

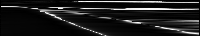

Supplement: Supplementary file 7 — Source Data [file 41467_2022_34941_MOESM7_ESM.zip › SI_fig08_fig09/51_1_sub_52_1__06_sumphase10_abs.bmp]

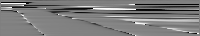

Supplement: Supplementary file 7 — Source Data [file 41467_2022_34941_MOESM7_ESM.zip › SI_fig08_fig09/51_1_sub_52_1__07_sumphase10.bmp]

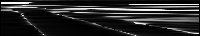

Supplement: Supplementary file 7 — Source Data [file 41467_2022_34941_MOESM7_ESM.zip › SI_fig08_fig09/51_1_sub_52_1__07_sumphase10_abs.bmp]

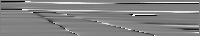

Supplement: Supplementary file 7 — Source Data [file 41467_2022_34941_MOESM7_ESM.zip › SI_fig08_fig09/51_1_sub_52_1__08_sumphase10.bmp]

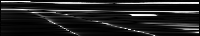

Supplement: Supplementary file 7 — Source Data [file 41467_2022_34941_MOESM7_ESM.zip › SI_fig08_fig09/51_1_sub_52_1__08_sumphase10_abs.bmp]

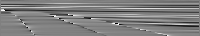

Supplement: Supplementary file 7 — Source Data [file 41467_2022_34941_MOESM7_ESM.zip › SI_fig08_fig09/51_1_sub_52_1__09_sumphase10.bmp]

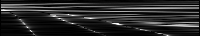

Supplement: Supplementary file 7 — Source Data [file 41467_2022_34941_MOESM7_ESM.zip › SI_fig08_fig09/51_1_sub_52_1__09_sumphase10_abs.bmp]

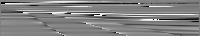

Supplement: Supplementary file 7 — Source Data [file 41467_2022_34941_MOESM7_ESM.zip › SI_fig08_fig09/51_1_sub_52_1__10_sumphase10.bmp]

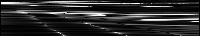

Supplement: Supplementary file 7 — Source Data [file 41467_2022_34941_MOESM7_ESM.zip › SI_fig08_fig09/51_1_sub_52_1__10_sumphase10_abs.bmp]

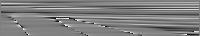

Supplement: Supplementary file 7 — Source Data [file 41467_2022_34941_MOESM7_ESM.zip › SI_fig08_fig09/51_1_sub_52_1__11_sumphase10.bmp]

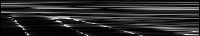

Supplement: Supplementary file 7 — Source Data [file 41467_2022_34941_MOESM7_ESM.zip › SI_fig08_fig09/51_1_sub_52_1__11_sumphase10_abs.bmp]

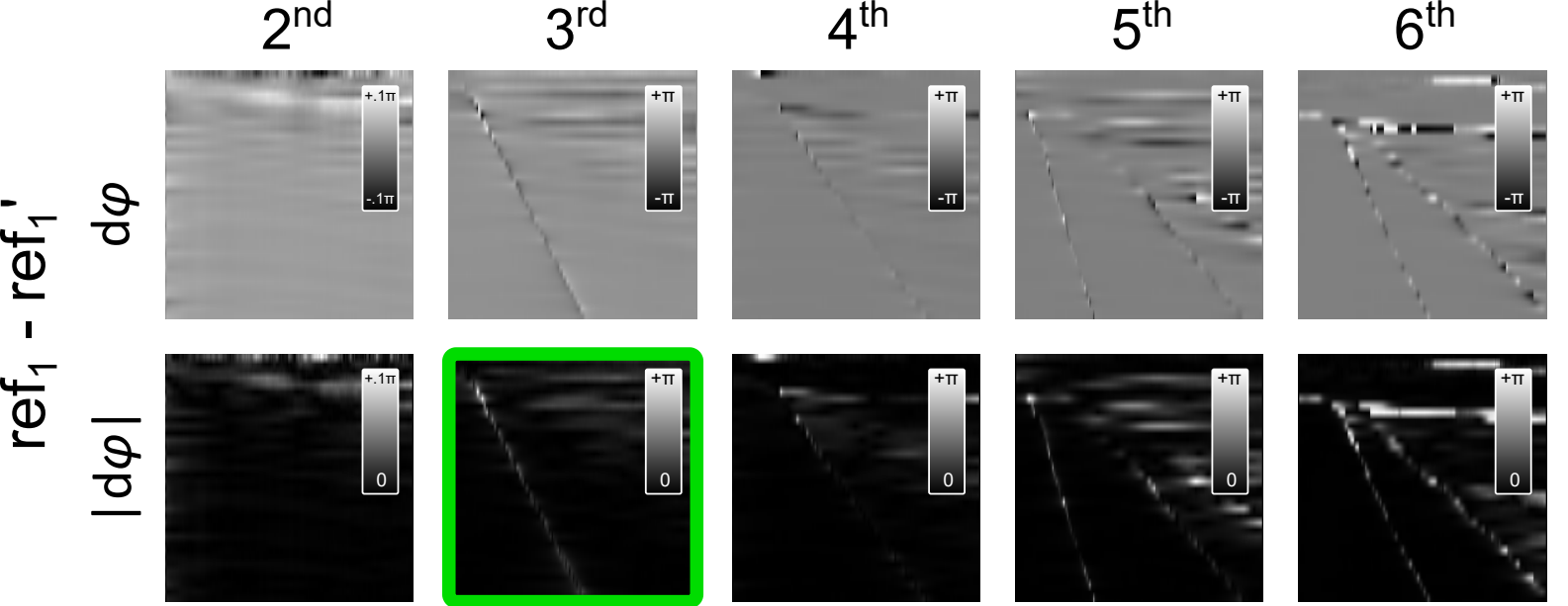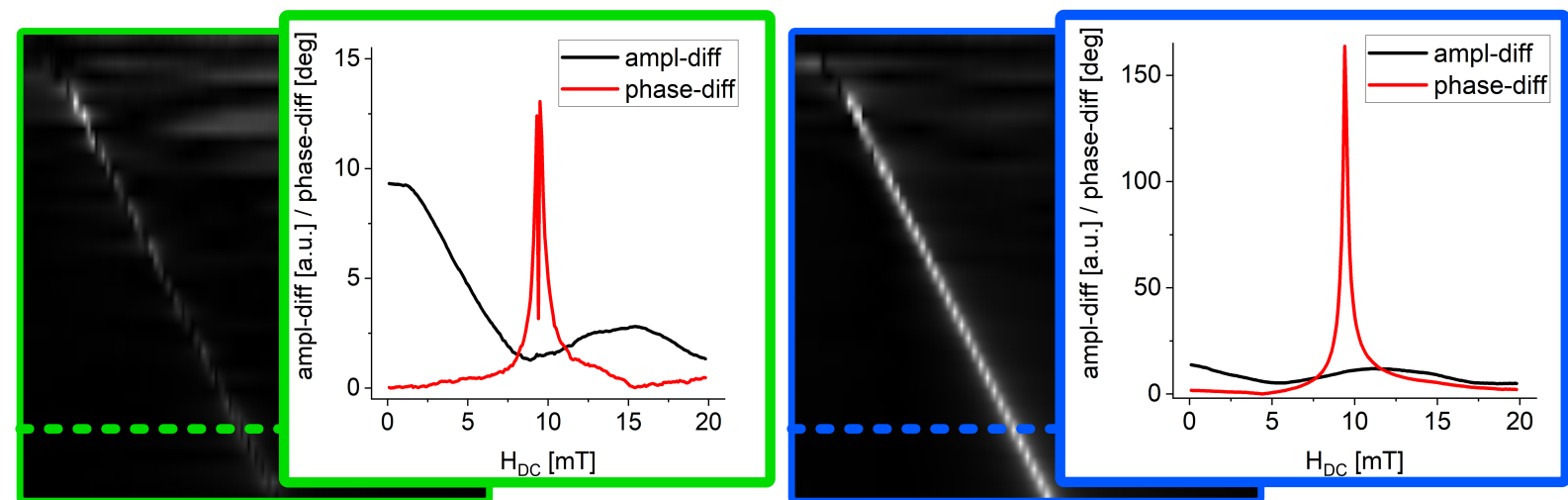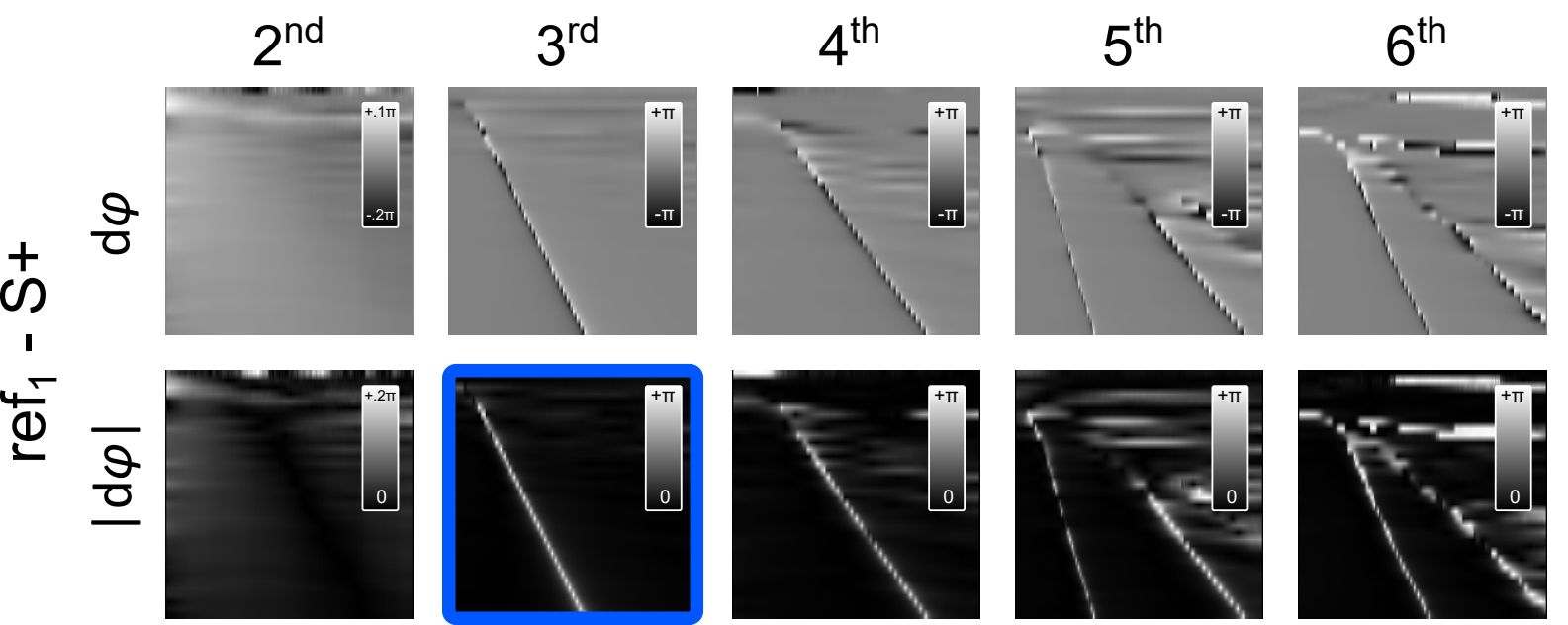

Supplement: Supplementary file 7 — Source Data [file 41467_2022_34941_MOESM7_ESM.zip › SI_fig08_fig09/SI_fig08.pdf]

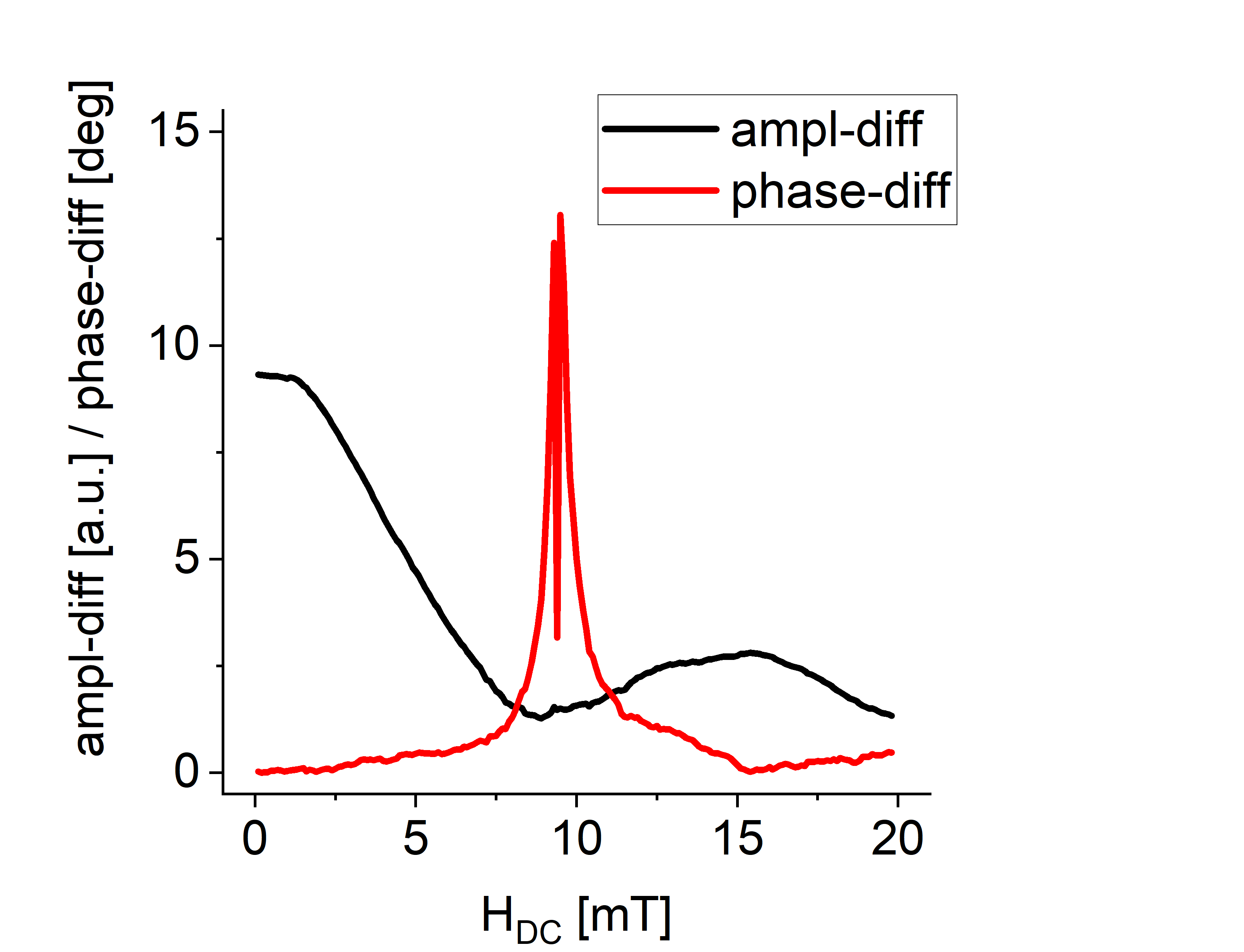

Supplement: Supplementary file 7 — Source Data [file 41467_2022_34941_MOESM7_ESM.zip › SI_fig08_fig09/SI_fig08_subplot1.png]

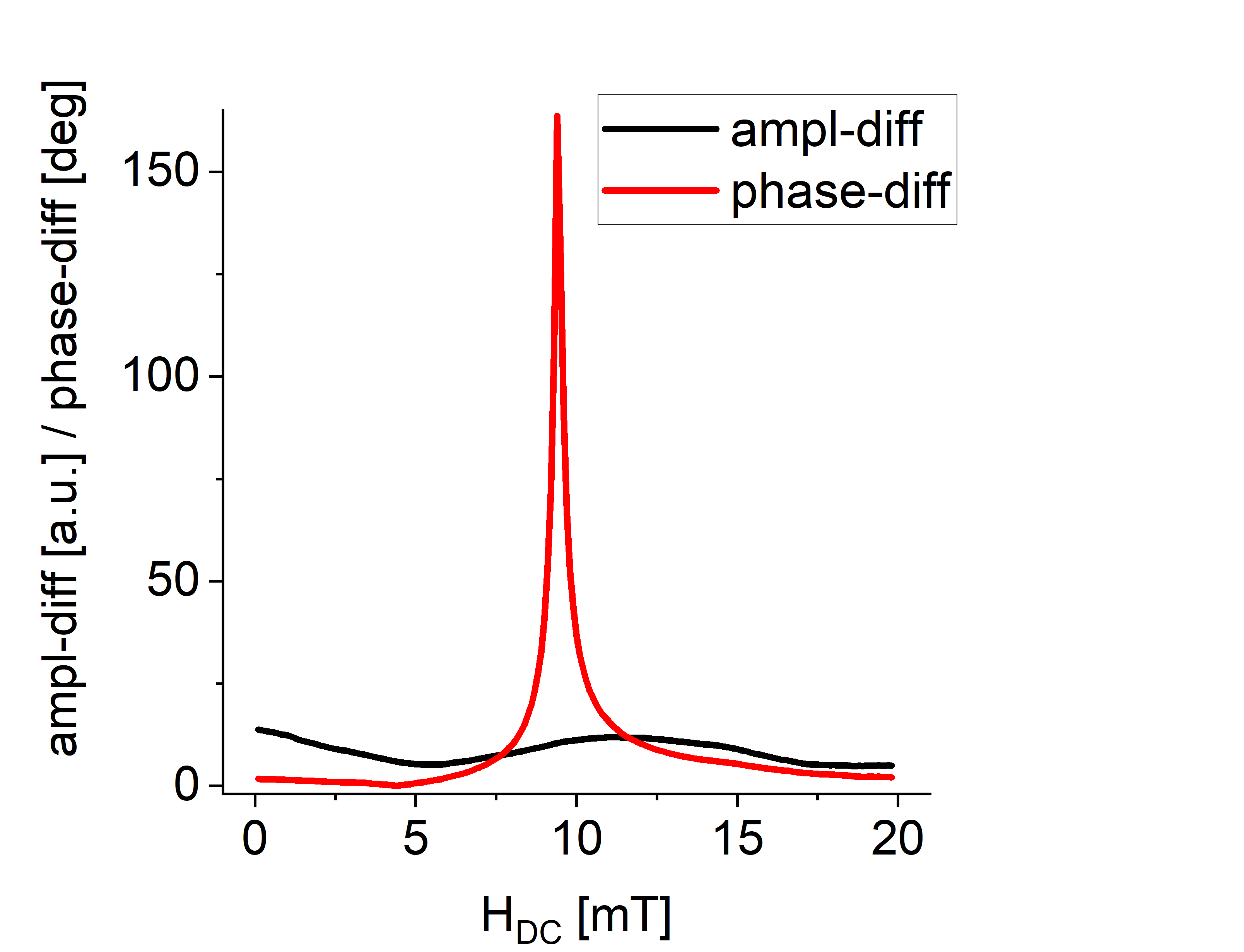

Supplement: Supplementary file 7 — Source Data [file 41467_2022_34941_MOESM7_ESM.zip › SI_fig08_fig09/SI_fig08_subplot2.png]

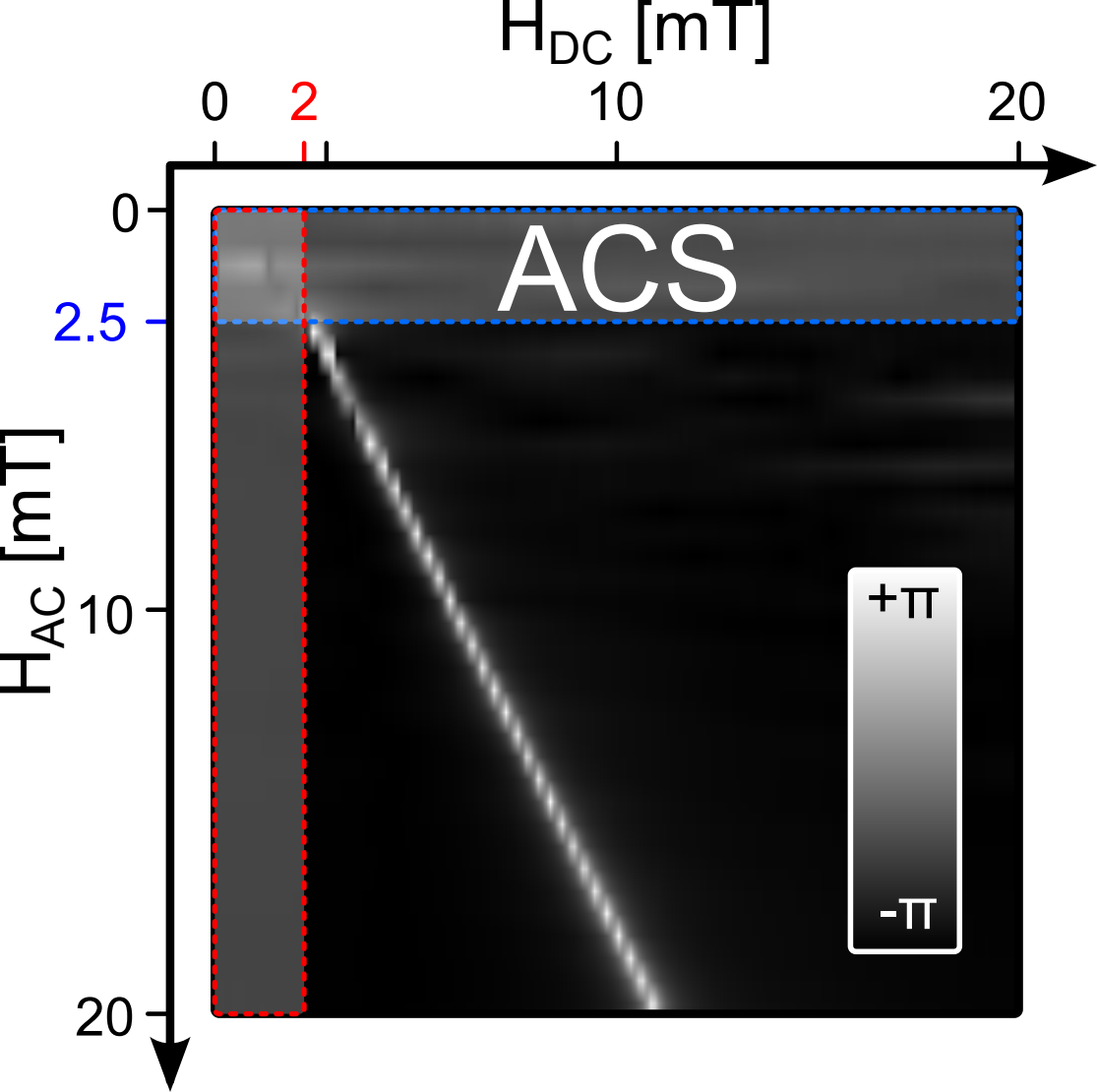

Supplement: Supplementary file 7 — Source Data [file 41467_2022_34941_MOESM7_ESM.zip › SI_fig08_fig09/SI_fig09.pdf]

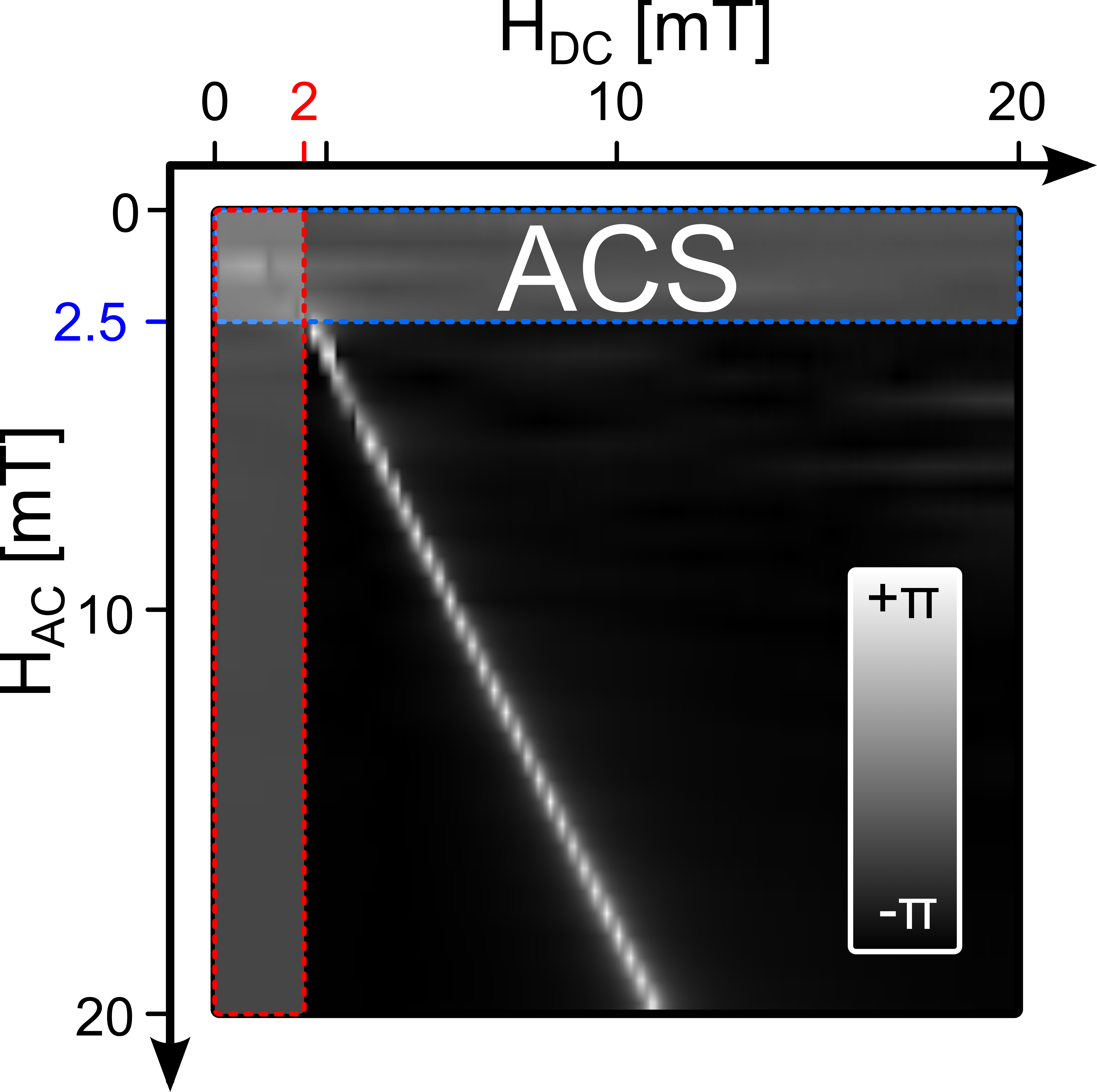

Supplement: Supplementary file 7 — Source Data [file 41467_2022_34941_MOESM7_ESM.zip › SI_fig08_fig09/SI_fig09.png]

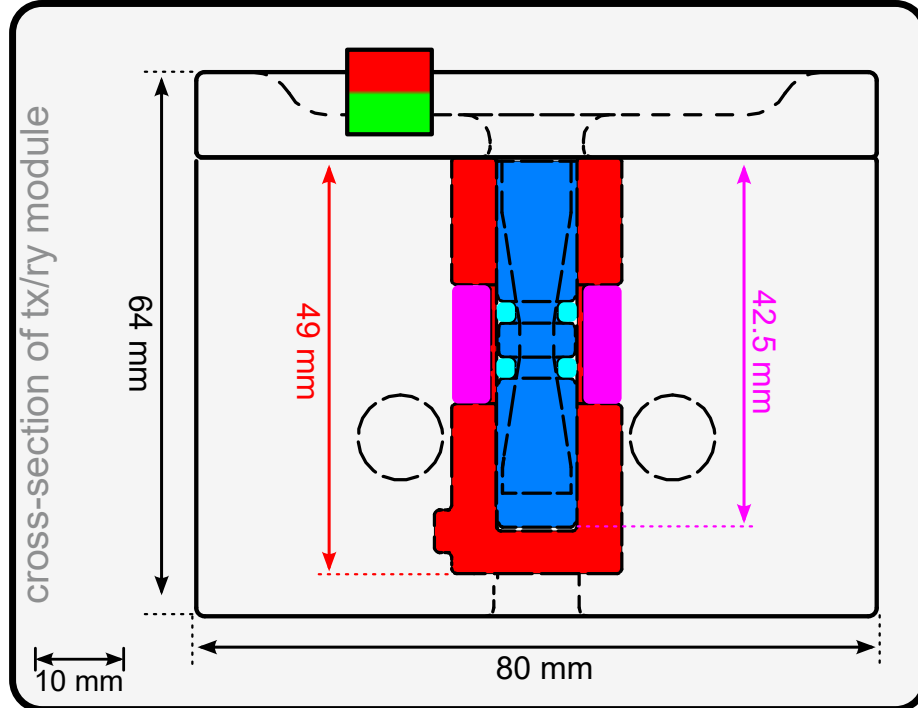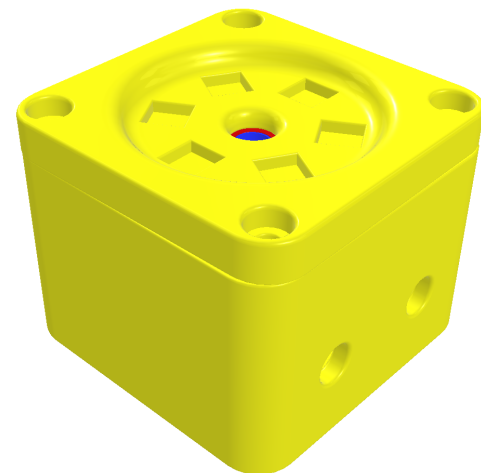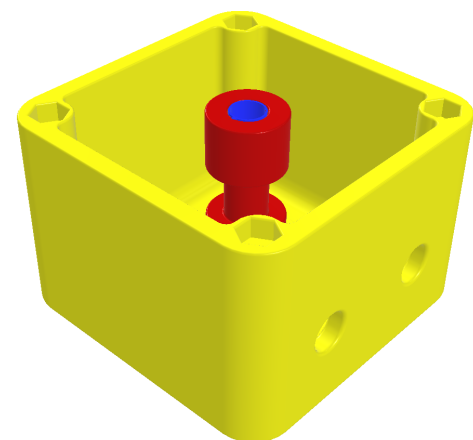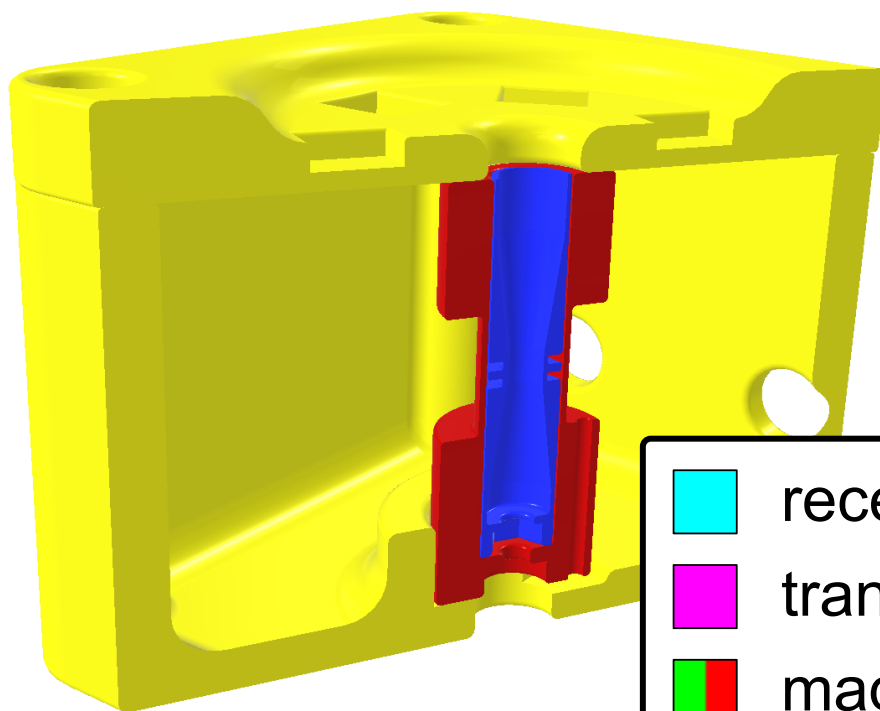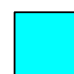

receive coil

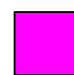

transmit coil

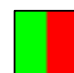

magnet

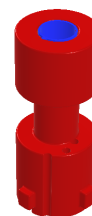

Supplement: Supplementary file 7 — Source Data [file 41467_2022_34941_MOESM7_ESM.zip › SI_fig10/SI_fig10.pdf]
