# Supplementary material for: Critical Offset Magnetic PArticle SpectroScopy for rapid and highly sensitive medical point-of-care diagnostics
Source: Nat Commun. 2022 Nov 24;13:7230. doi: 10.1038/s41467-022-34941-y (PMC9700695; doi:10.1038/s41467-022-34941-y)
Supplement: Supplementary file 6 — Reporting Summary [file 41467_2022_34941_MOESM6_ESM.pdf]

nature research

Corresponding author(s): Dr. rer nat Patrick Vogel

Last updated by author(s): 2022.11.08

Reporting Summary

Nature Research wishes to improve the reproducibility of the work that we publish. This form provides structure for consistency and transparency in reporting. For further information on Nature Research policies, see our Editorial Policies and the Editorial Policy Checklist.

Statistics

For all statistical analyses, confirm that the following items are present in the figure legend, table legend, main text, or Methods section.

n/a	Confirmed	
☐	☒	The exact sample size (n) for each experimental group/condition, given as a discrete number and unit of measurement
☐	☒	A statement on whether measurements were taken from distinct samples or whether the same sample was measured repeatedly
☒	☐	The statistical test(s) used AND whether they are one- or two-sided<br>*Only common tests should be described solely by name; describe more complex techniques in the Methods section.*
☐	☒	A description of all covariates tested
☐	☒	A description of any assumptions or corrections, such as tests of normality and adjustment for multiple comparisons
☐	☒	A full description of the statistical parameters including central tendency (e.g. means) or other basic estimates (e.g. regression coefficient) AND variation (e.g. standard deviation) or associated estimates of uncertainty (e.g. confidence intervals)
☒	☐	For null hypothesis testing, the test statistic (e.g. F, t, r) with confidence intervals, effect sizes, degrees of freedom and P value noted<br>*Give P values as exact values whenever suitable.*
☒	☐	For Bayesian analysis, information on the choice of priors and Markov chain Monte Carlo settings
☒	☐	For hierarchical and complex designs, identification of the appropriate level for tests and full reporting of outcomes
☒	☐	Estimates of effect sizes (e.g. Cohen's d, Pearson's r), indicating how they were calculated

Our web collection on statistics for biologists contains articles on many of the points above.

Software and code

Policy information about availability of computer code

Data collection	Kaluza 2.0; Zetasizer Software v7.13; SoftMax® Pro 7;
Data analysis	Origin 2021b; Embarcadero Rad Studio 11; Inkscape V1.2; PSoC Creator 4.4

For manuscripts utilizing custom algorithms or software that are central to the research but not yet described in published literature, software must be made available to editors and reviewers. We strongly encourage code deposition in a community repository (e.g. GitHub). See the Nature Research guidelines for submitting code & software for further information.

Data

Policy information about availability of data

All manuscripts must include a data availability statement. This statement should provide the following information, where applicable:

- Accession codes, unique identifiers, or web links for publicly available datasets
- A list of figures that have associated raw data
- A description of any restrictions on data availability

Source data are provided with this paper. Raw data, preprocessed measurement data, as well as specific source files (Inkscape V1.2, Origin 2021b) for generating relevant graphs are available on zenodo.org (https://doi.org/10.5281/zenodo.7304376).
Source codes are provided with this paper. Source code of user-defined data analysis software (Embarcadero RAD Studio 11) for data visualization and processing is provided on zenodo.org (https://doi.org/10.5281/zenodo.7304376).

Field-specific reporting

Please select the one below that is the best fit for your research. If you are not sure, read the appropriate sections before making your selection.

- [x] Life sciences
- [ ] Behavioural & social sciences
- [ ] Ecological, evolutionary & environmental sciences

For a reference copy of the document with all sections, see nature.com/documents/nr-reporting-summary-flat.pdf

Life sciences study design

All studies must disclose on these points even when the disclosure is negative.

Sample size	The sample size of 50µl in a 0.5ml Eppicap is a useful size used for bioassays. The COMPASS device has been specifically adjusted to that size.
Data exclusions	No data were exculuded.
Replication	Measurements with different samples (>30) have been performed multiple times to ensure robust results (>20k single measurements).
Randomization	We strongly make sure, that the provided data are correct. For that, we build 3 different mobile COMPASS prototypes and 3 benchtop COMPASS systems.
Blinding	For evaluation, we also performed blinded measurements to ensure non-biased data interpretation. However, since the signal is easy to interpret, a blinding process is not relevant here.

Reporting for specific materials, systems and methods

We require information from authors about some types of materials, experimental systems and methods used in many studies. Here, indicate whether each material, system or method listed is relevant to your study. If you are not sure if a list item applies to your research, read the appropriate section before selecting a response.

Materials & experimental systems

n/a	Involved in the study	
[ ]	[x]	Antibodies
[x]	[ ]	Eukaryotic cell lines
[x]	[ ]	Palaeontology and archaeology
[x]	[ ]	Animals and other organisms
[ ]	[x]	Human research participants
[x]	[ ]	Clinical data
[x]	[ ]	Dual use research of concern

Methods

n/a	Involved in the study	
[x]	[ ]	ChIP-seq
[ ]	[x]	Flow cytometry
[x]	[ ]	MRI-based neuroimaging

Antibodies

Antibodies used	SARS-CoV/SARS-CoV-2 Spike antibody,Chimeric MAb; Unconjugated; (Sino Biological, Cat: 40150-D001)<br><br>MERS-CoV Spike Antibody, Rabbit PAb, Unconjugated; (Sino Biological, Cat: 40069-T62)
Validation	Monoclonal mouse (variable region) / human (kappa / IgG1 constant) chimeric antibody Clone #D001; Unconjugated;Validated applications: ELISA, FCM,ICC/IF, Neutralization.<br>(https://cdn1.sinobiological.com/reagent/antibody-application/elisa-protocol-en.pdf and https://cdn1.sinobiological.com/reagent/antibody-application/fcm-protocol-en.pdf and https://cdn1.sinobiological.com/reagent/antibody-application/if-protocol-en.pdf)<br>Shen S, et al. (2007) Expression, glycosylation, and modification of the spike (S) glycoprotein of SARS CoV. Methods Mol Biol. 379: 127-35.<br>Du L, et al. (2009) The spike protein of SARS-CoV--a target for vaccine and therapeutic development. Nat Rev Microbiol. 7 (3): 226-36.<br>Xiao X, et al. (2004) The SARS-CoV S glycoprotein. Cell Mol Life Sci. 61 (19-20): 2428-30.<br><br>MERS-CoV Spike Antibody, Rabbit PAb, Antigen Affinity Purified, Cat: 40069-T62<br>((https://cdn1.sinobiological.com/reagent/antibody-application/wb-protocol-en.pdf and https://cdn1.sinobiological.com/reagent/antibody-application/elisa-protocol-en.pdf)<br><br>Shen S, et al. (2007) Expression, glycosylation, and modification of the spike (S) glycoprotein of SARS CoV. Methods Mol Biol. 379: 127-35.<br>Du L, et al. (2009) The spike protein of SARS-CoV--a target for vaccine and therapeutic development. Nat Rev Microbiol. 7 (3): 226-36.<br>Xiao X, et al. (2004) The SARS-CoV S glycoprotein. Cell Mol Life Sci. 61 (19-20): 2428-30.

Human research participants

Policy information about studies involving human research participants

Population characteristics	Lab members
Recruitment	Serum samples were obtained from lab members with informed consent.
Ethics oversight	The local ethics committee of University Hospital Würzburg approved the study (AZ 35/07)

Note that full information on the approval of the study protocol must also be provided in the manuscript.

Flow Cytometry

Plots

Confirm that:

- [x] The axis labels state the marker and fluorochrome used (e.g. CD4-FITC).
- [x] The axis scales are clearly visible. Include numbers along axes only for bottom left plot of group (a 'group' is an analysis of identical markers).
- [ ] All plots are contour plots with outliers or pseudocolor plots.
- [x] A numerical value for number of cells or percentage (with statistics) is provided.

Methodology

Sample preparation	The preperation of the samples can be found in the methods.
Instrument	Gallios Beckman Coulter
Software	Kaluza Analysis 2.0
Cell population abundance	Cellsorting was not performed.
Gating strategy	Particles were first gated for FCS/SSC. Further gating was done according to the fluorecent dye.

- [x] Tick this box to confirm that a figure exemplifying the gating strategy is provided in the Supplementary Information.
