# Supplementary material for: Critical Offset Magnetic PArticle SpectroScopy for rapid and highly sensitive medical point-of-care diagnostics
Source: Nat Commun. 2022 Nov 24;13:7230. doi: 10.1038/s41467-022-34941-y (PMC9700695; doi:10.1038/s41467-022-34941-y)

rapid SARS-CoV 2 antibody test with COMPASS

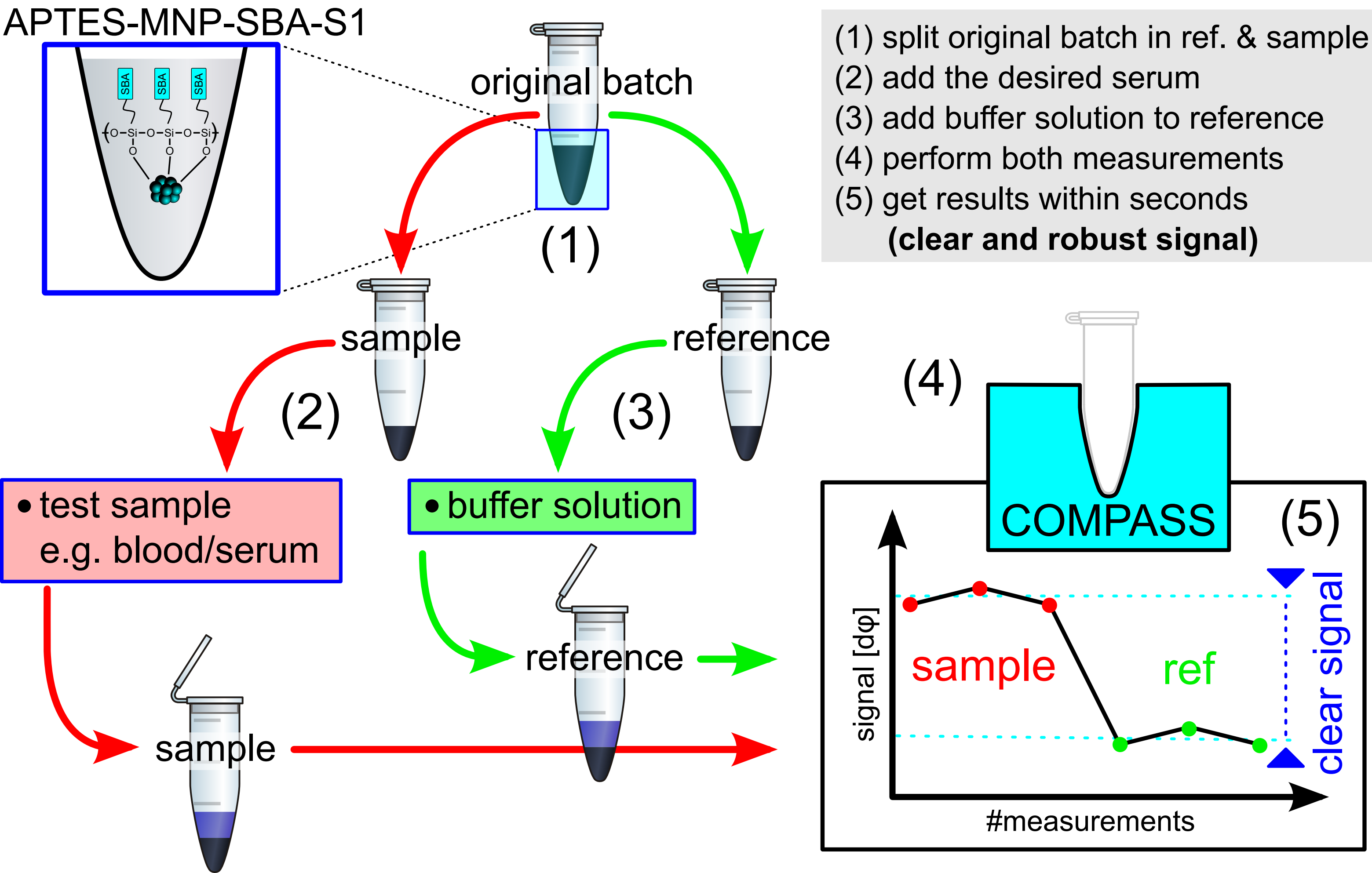

Supplement: Supplementary file 7 — Source Data [file 41467_2022_34941_MOESM7_ESM.zip › SI_fig14/SI_fig14.pdf]
