# Supplementary figures and images for: Critical Offset Magnetic PArticle SpectroScopy for rapid and highly sensitive medical point-of-care diagnostics (part 2 of 2)
Source: Nat Commun. 2022 Nov 24;13:7230. doi: 10.1038/s41467-022-34941-y (PMC9700695; doi:10.1038/s41467-022-34941-y)

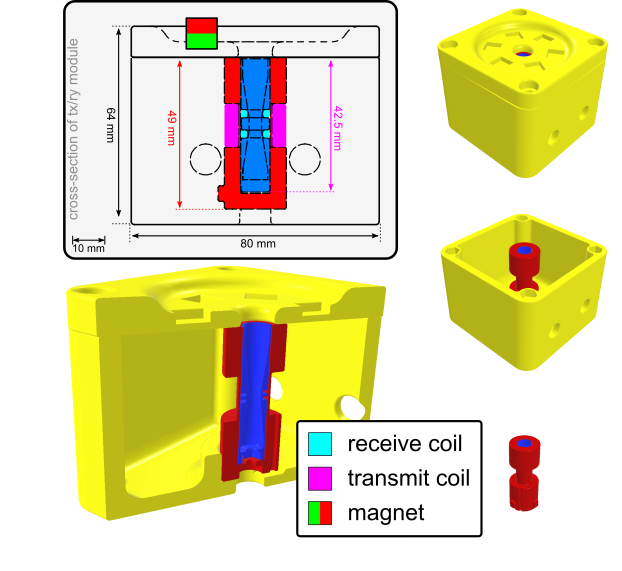

Supplement: Supplementary file 7 — Source Data [file 41467_2022_34941_MOESM7_ESM.zip › SI_fig10/SI_fig10.png]

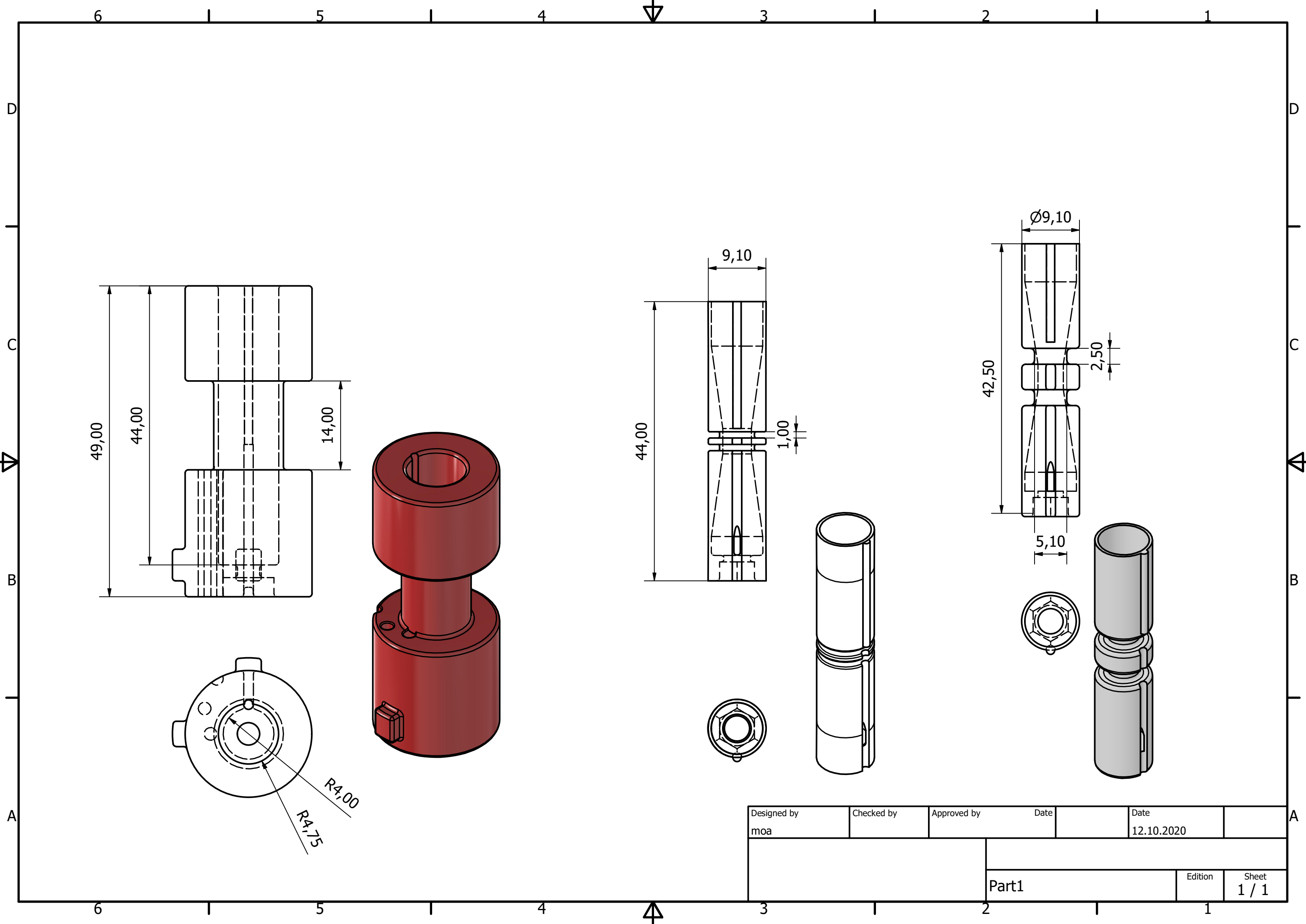

|                    |            |             |         |                    |  |
|--------------------|------------|-------------|---------|--------------------|--|
| Designed by<br>moa | Checked by | Approved by | Date    | Date<br>12.10.2020 |  |
|                    |            |             | Part1   |                    |  |
|                    |            |             | Edition | Sheet<br>1 / 1     |  |

Supplement: Supplementary file 7 — Source Data [file 41467_2022_34941_MOESM7_ESM.zip › SI_fig11/raw.pdf]

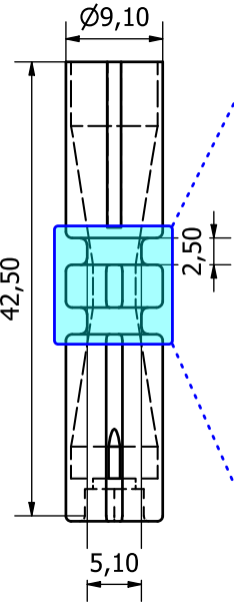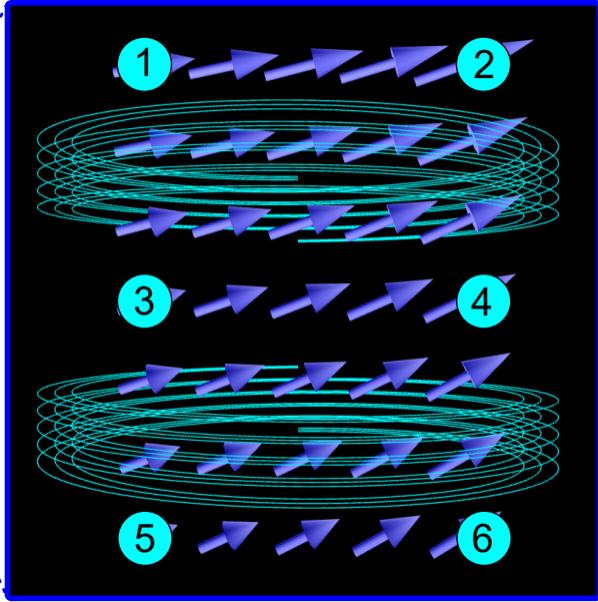

| marker | B-field [mT] |
|--------|--------------|
| 1      | 6.8          |
| 2      | 10.0         |
| 3      | 5.8          |
| 4      | 7.3          |
| 5      | 5.0          |
| 6      | 6.6          |

Supplement: Supplementary file 7 — Source Data [file 41467_2022_34941_MOESM7_ESM.zip › SI_fig11/SI_fig11.pdf]

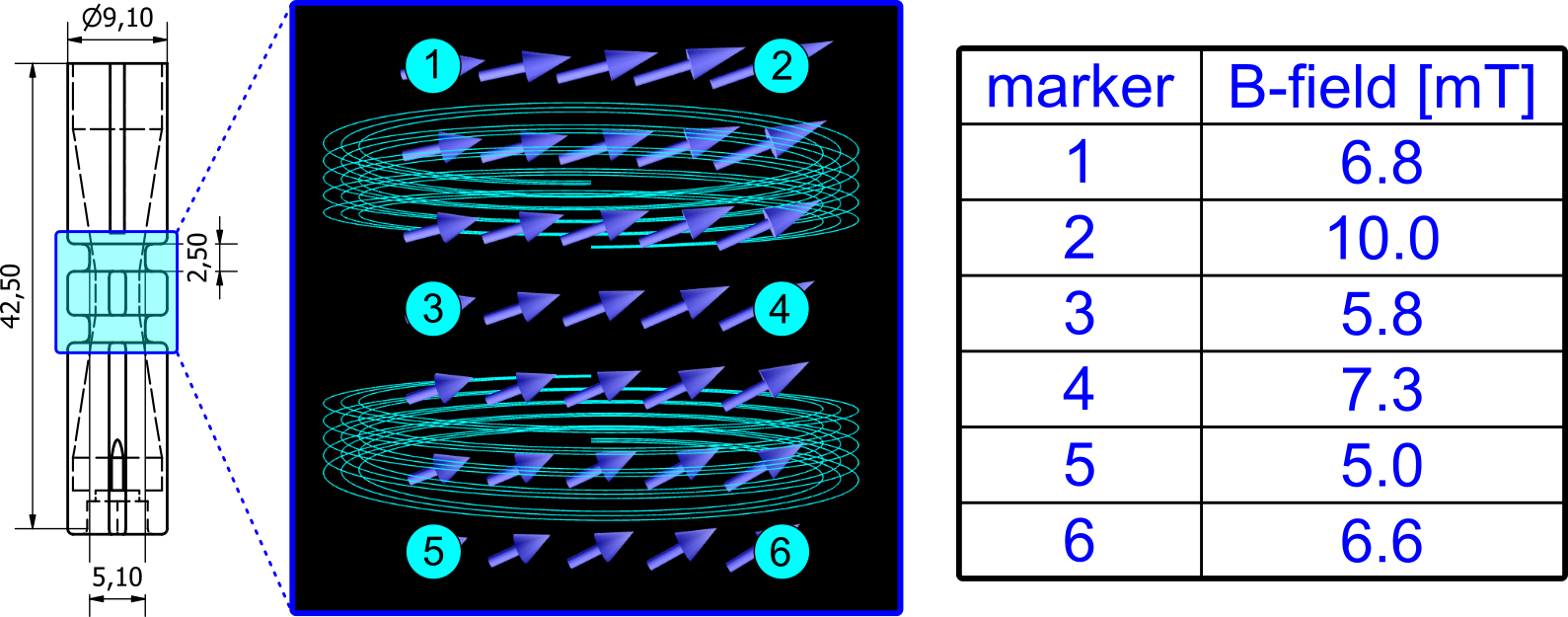

Supplement: Supplementary file 7 — Source Data [file 41467_2022_34941_MOESM7_ESM.zip › SI_fig11/SI_fig11.png]

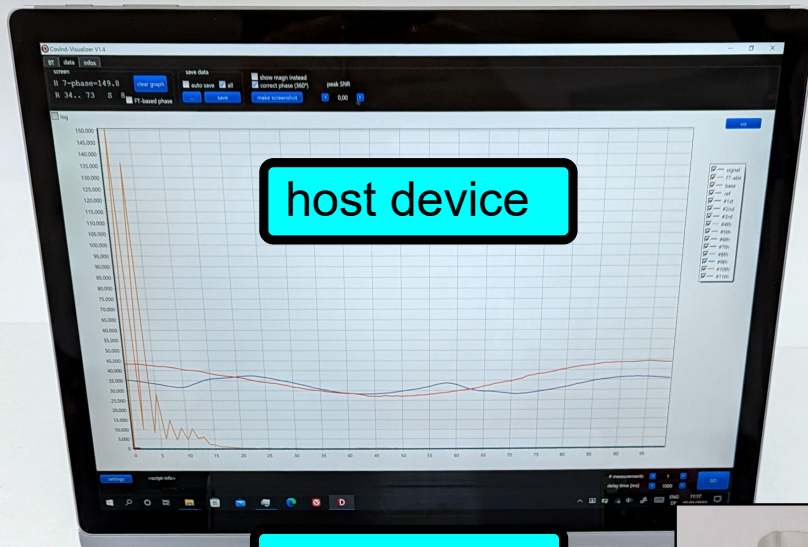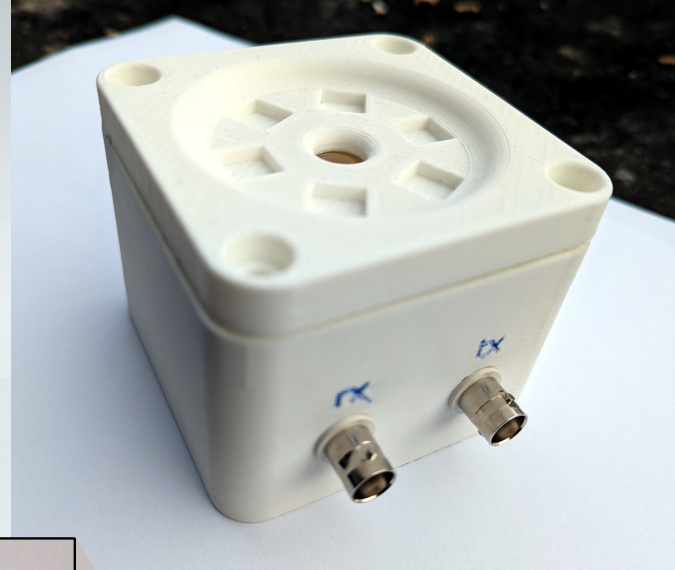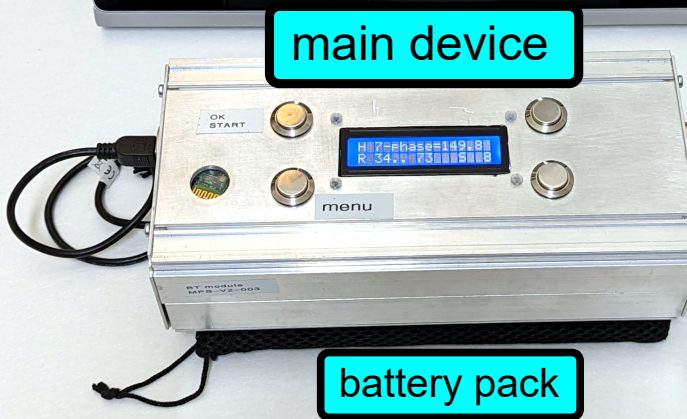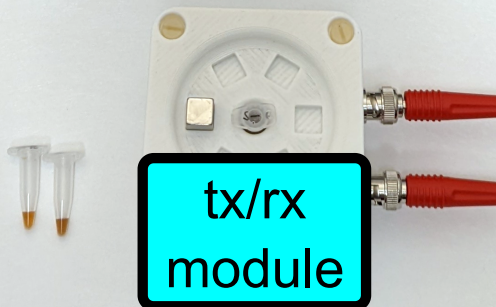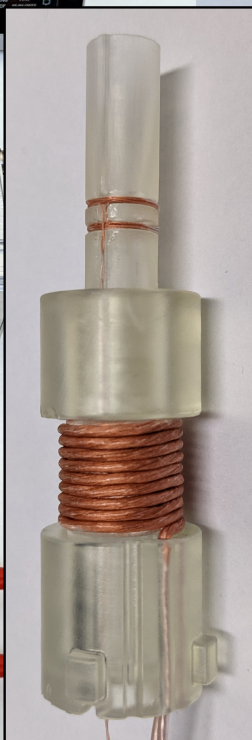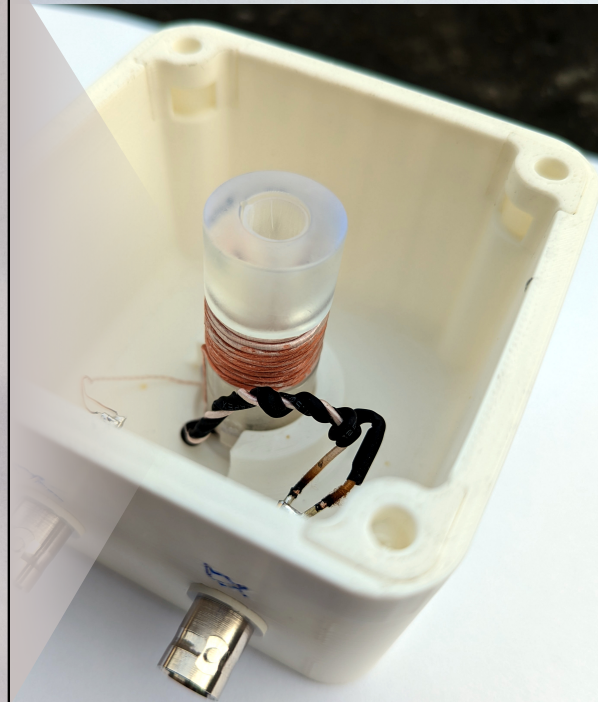

Supplement: Supplementary file 7 — Source Data [file 41467_2022_34941_MOESM7_ESM.zip › SI_fig12/SI_fig12.pdf]

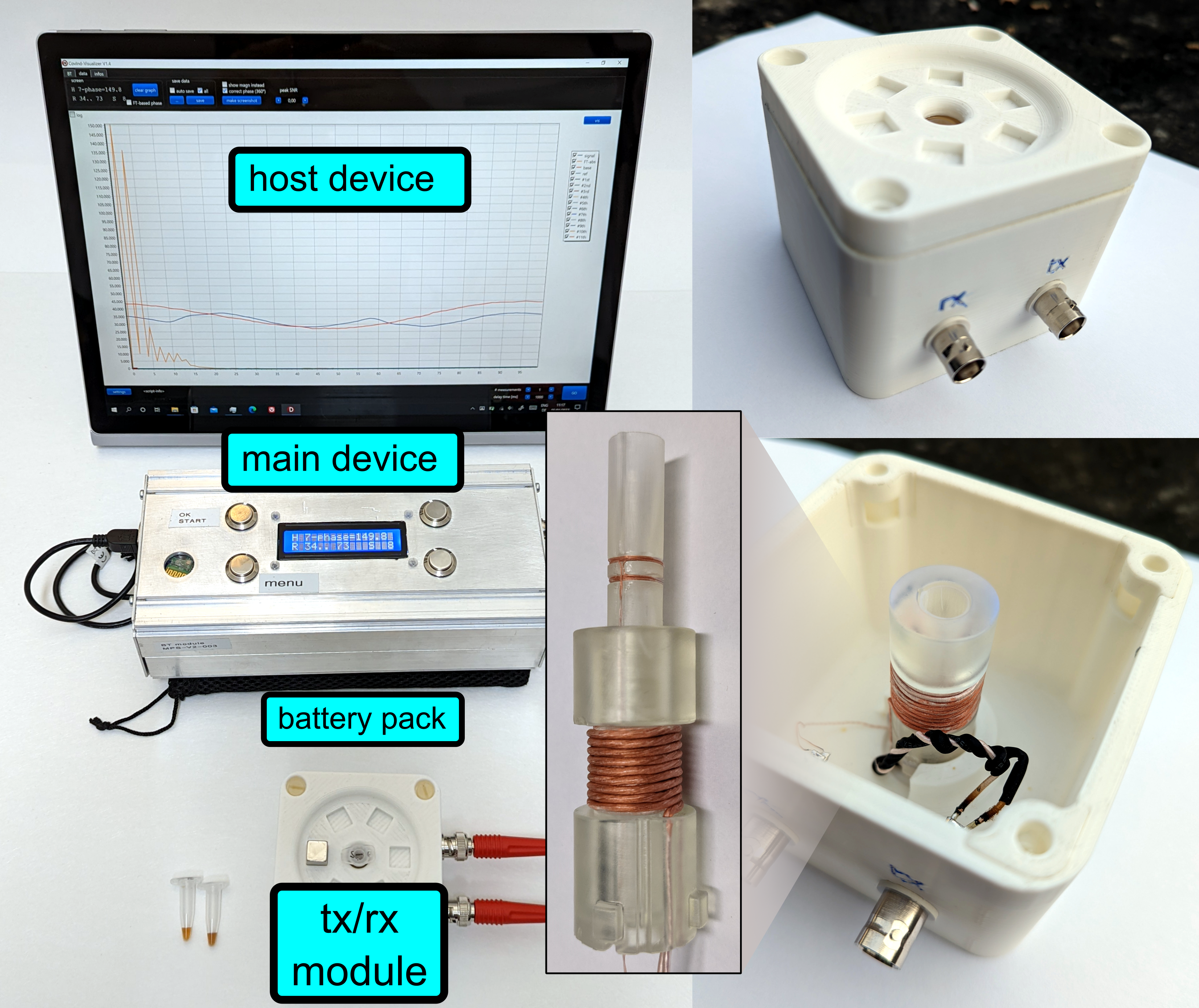

Supplement: Supplementary file 7 — Source Data [file 41467_2022_34941_MOESM7_ESM.zip › SI_fig12/SI_fig12.png]

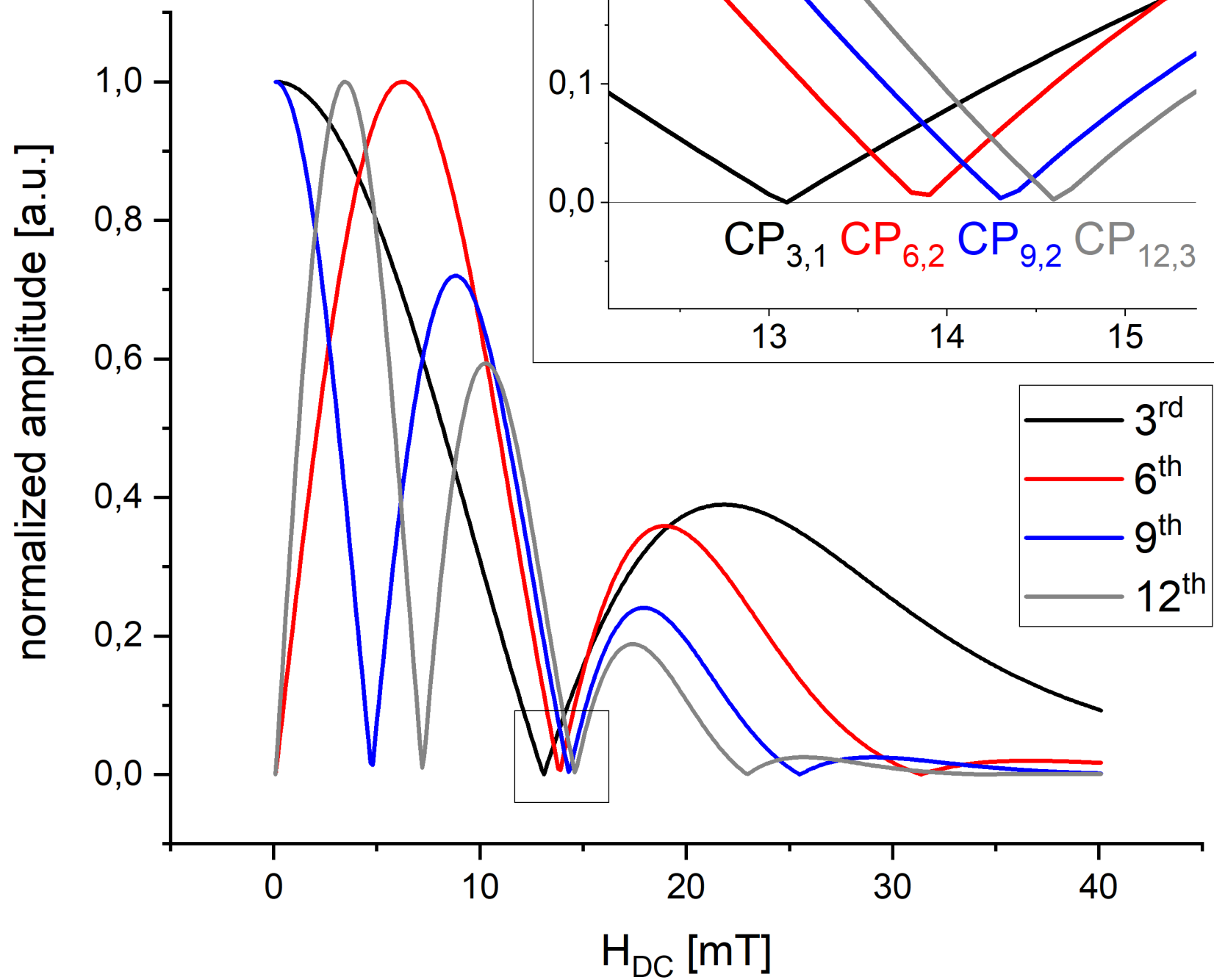

Supplement: Supplementary file 7 — Source Data [file 41467_2022_34941_MOESM7_ESM.zip › SI_fig13/SI_fig13.pdf]

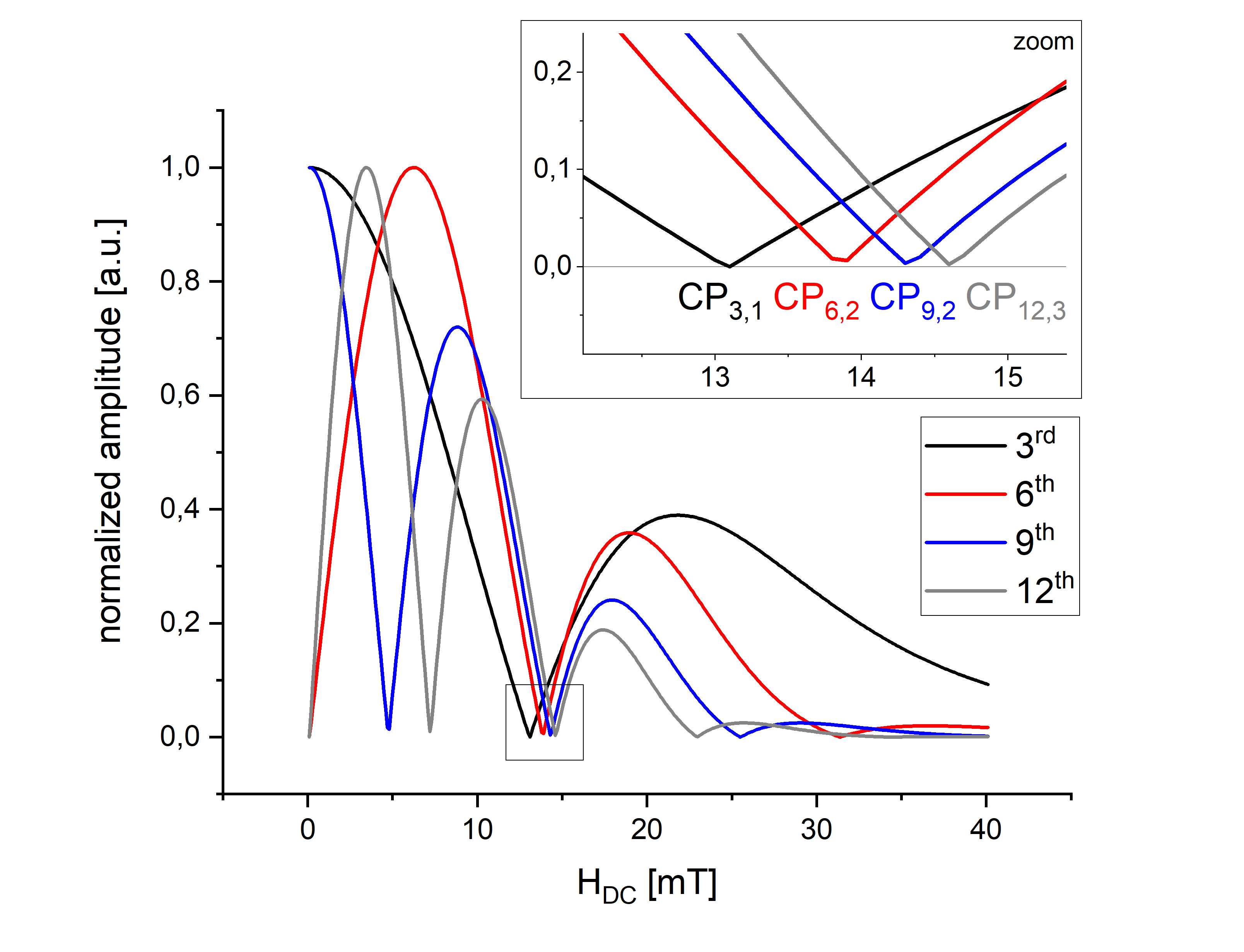

Supplement: Supplementary file 7 — Source Data [file 41467_2022_34941_MOESM7_ESM.zip › SI_fig13/SI_fig13.png]

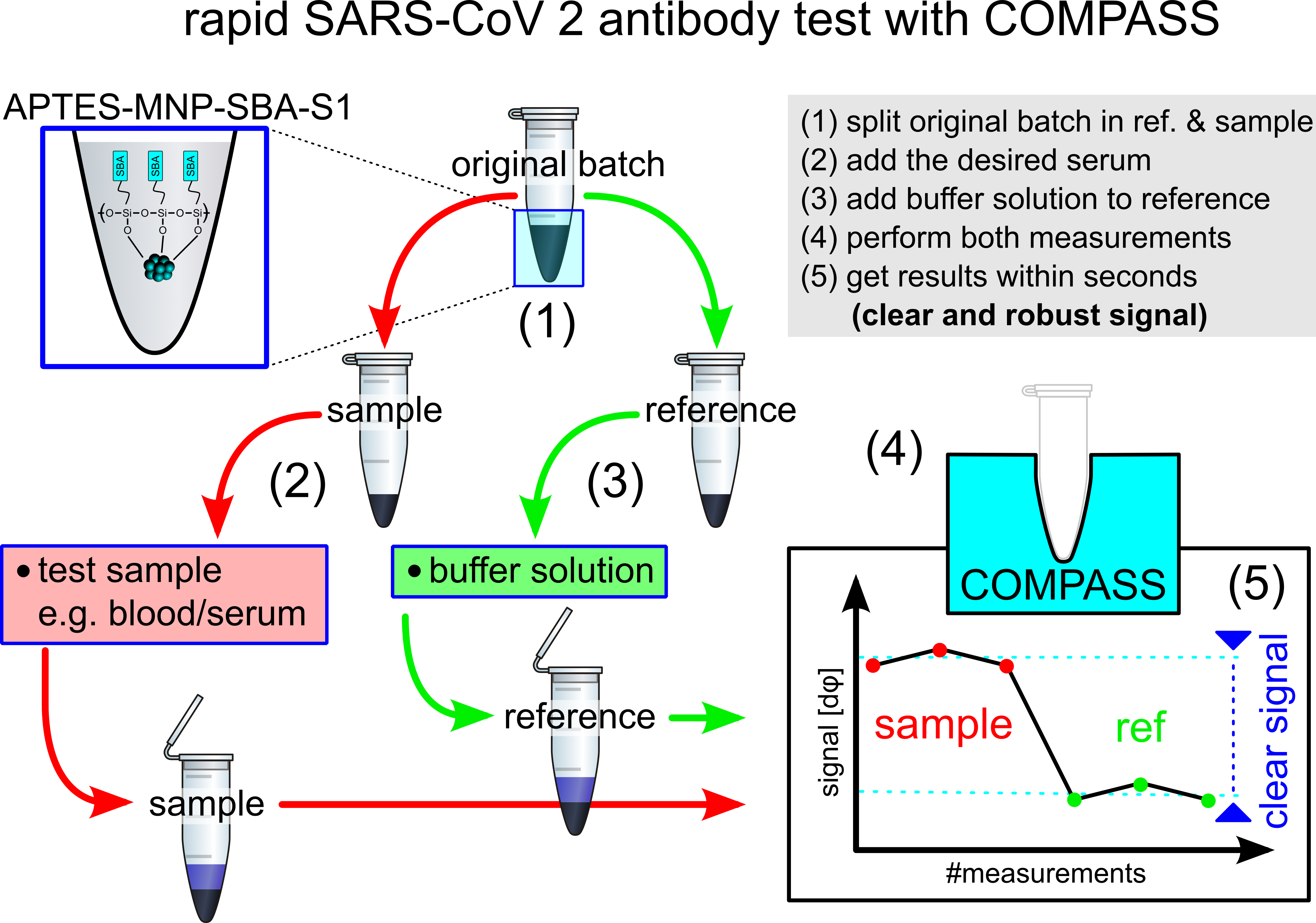

Supplement: Supplementary file 7 — Source Data [file 41467_2022_34941_MOESM7_ESM.zip › SI_fig14/SI_fig14.png]

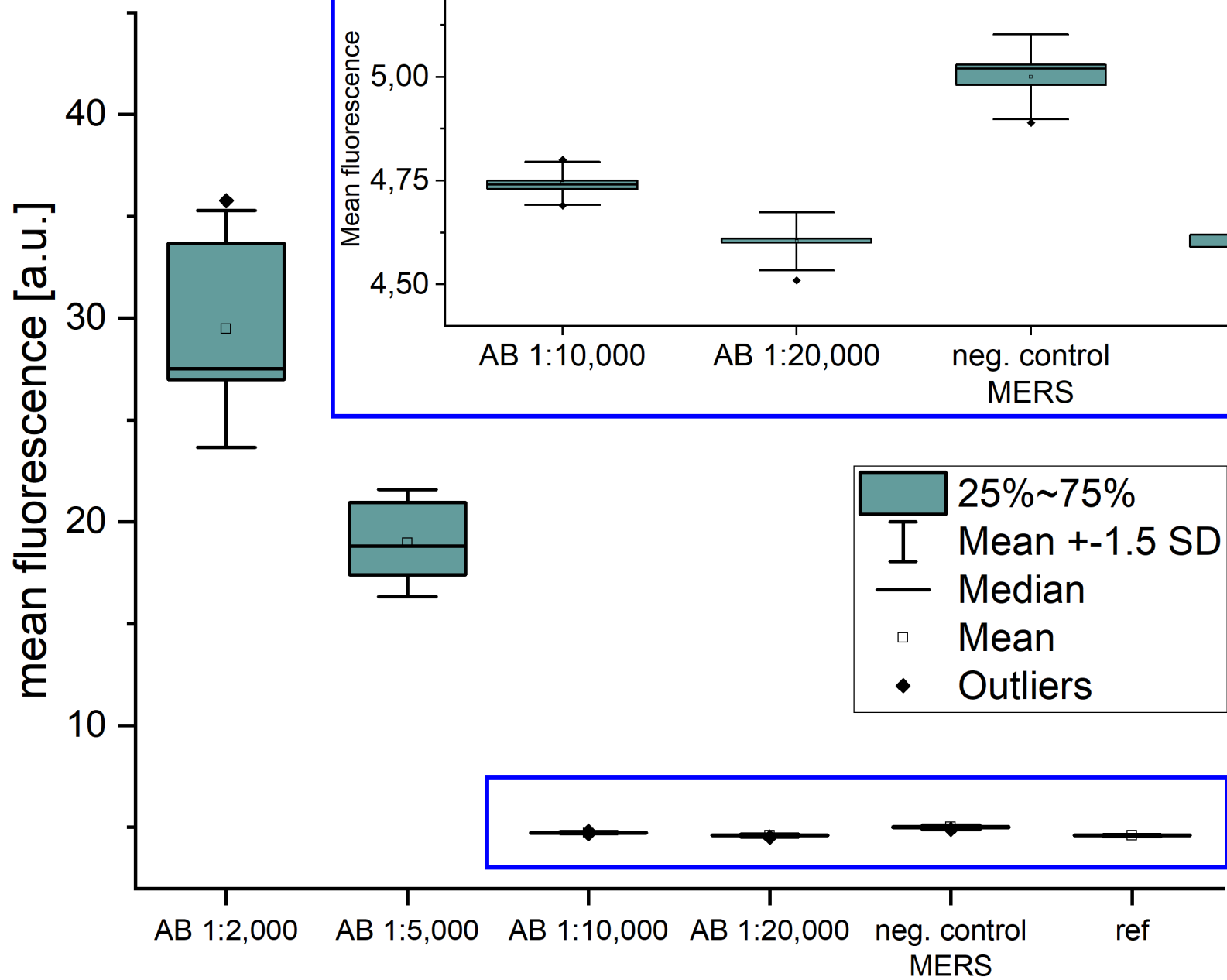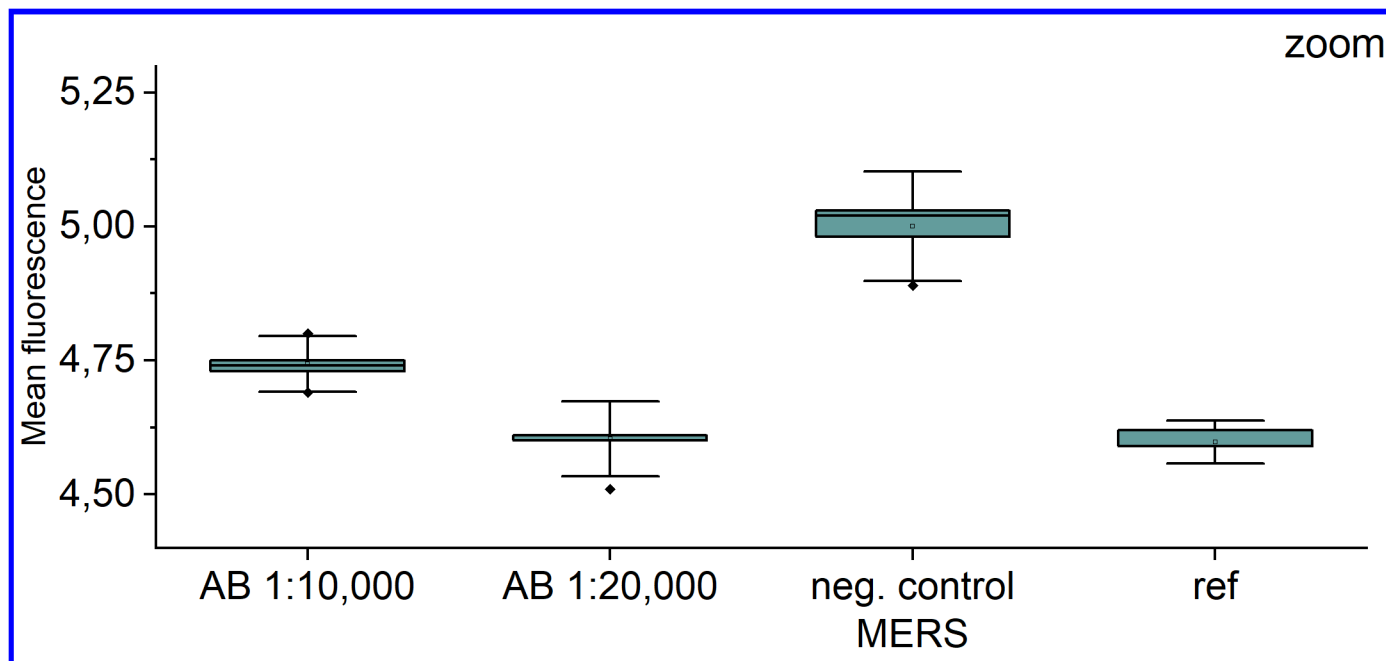

Supplement: Supplementary file 7 — Source Data [file 41467_2022_34941_MOESM7_ESM.zip › SI_fig15/SI_fig15.pdf]

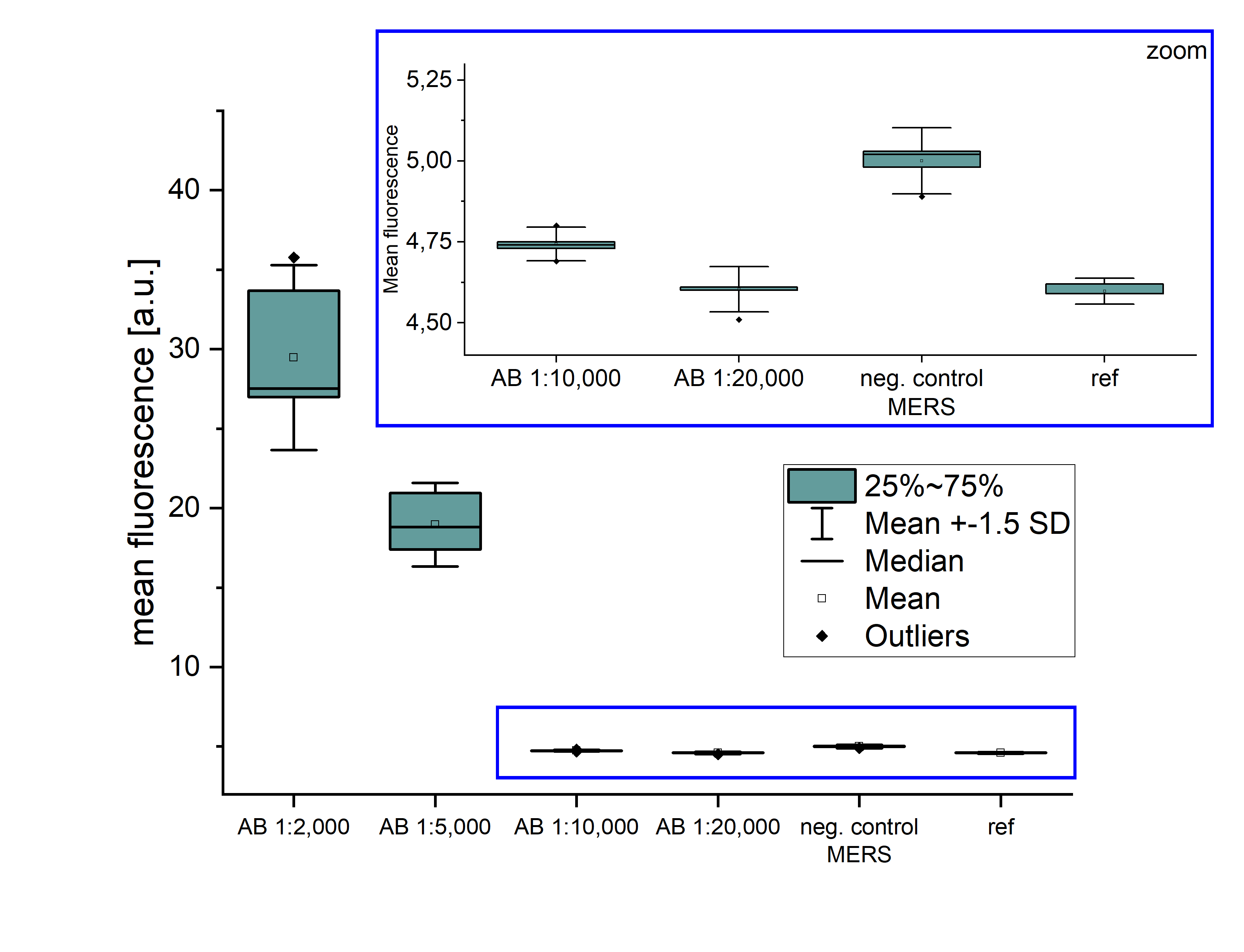

Supplement: Supplementary file 7 — Source Data [file 41467_2022_34941_MOESM7_ESM.zip › SI_fig15/SI_fig15.png]

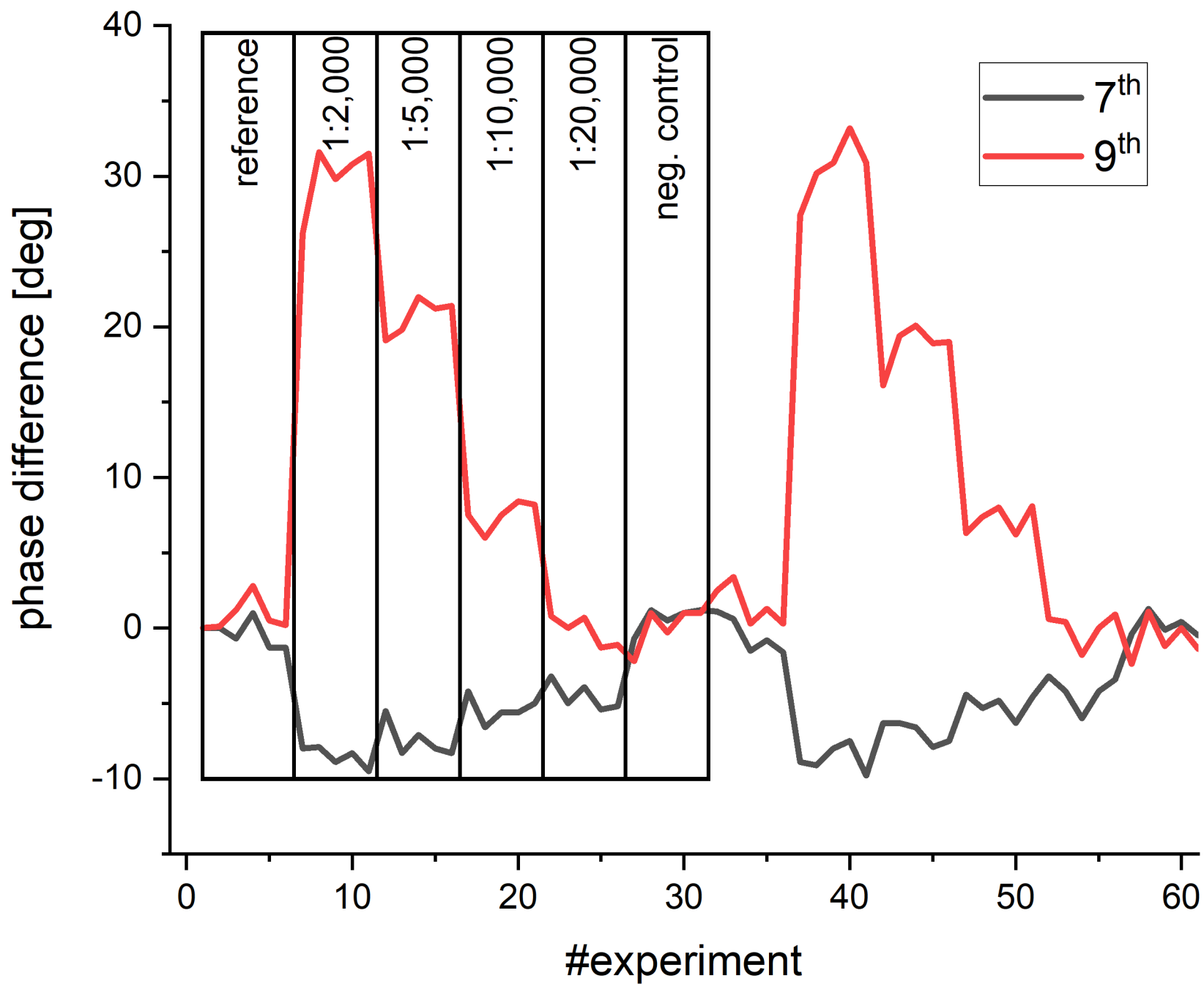

Supplement: Supplementary file 7 — Source Data [file 41467_2022_34941_MOESM7_ESM.zip › SI_fig16/SI_fig16_1.pdf]

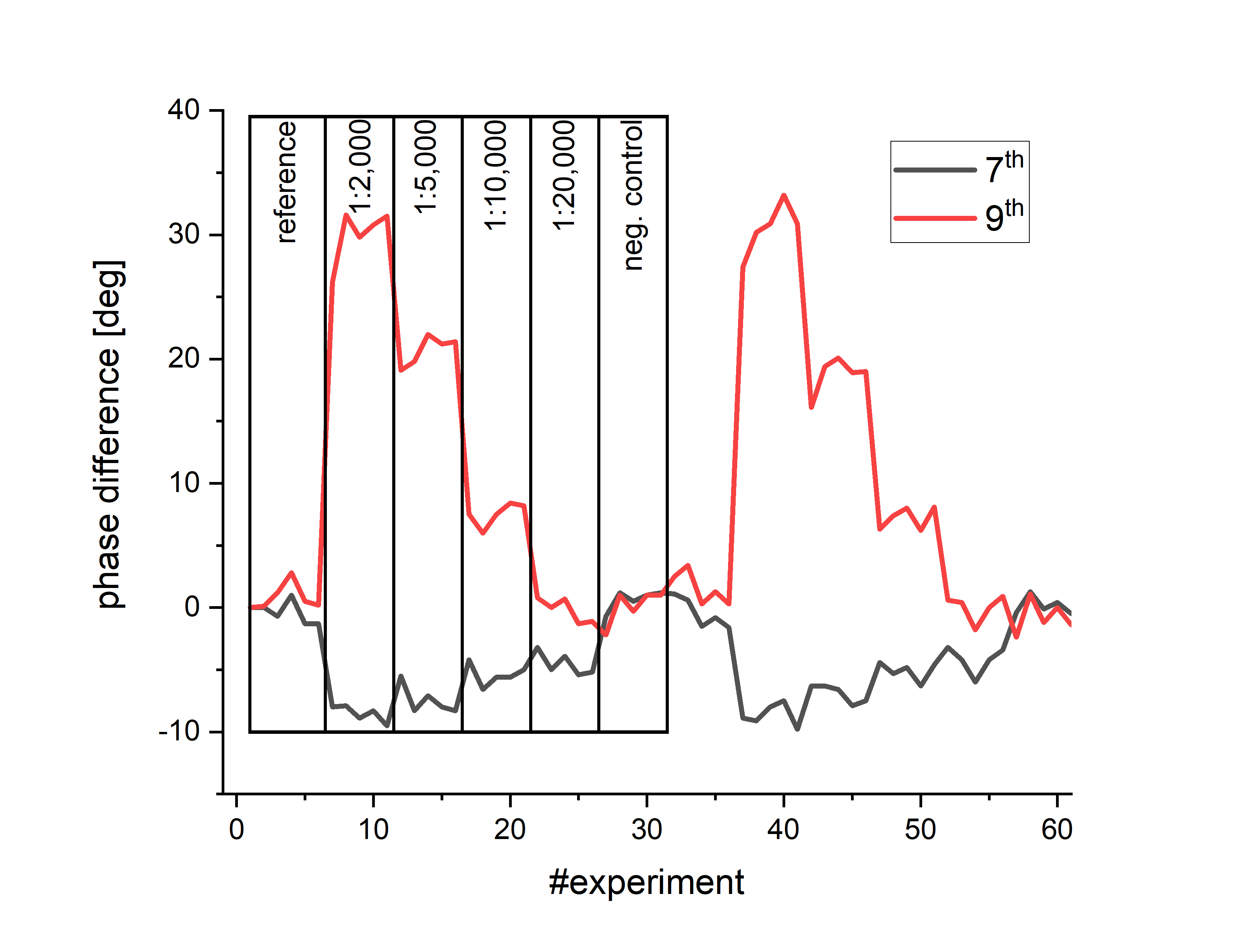

Supplement: Supplementary file 7 — Source Data [file 41467_2022_34941_MOESM7_ESM.zip › SI_fig16/SI_fig16_1.png]

## 7th harmonic

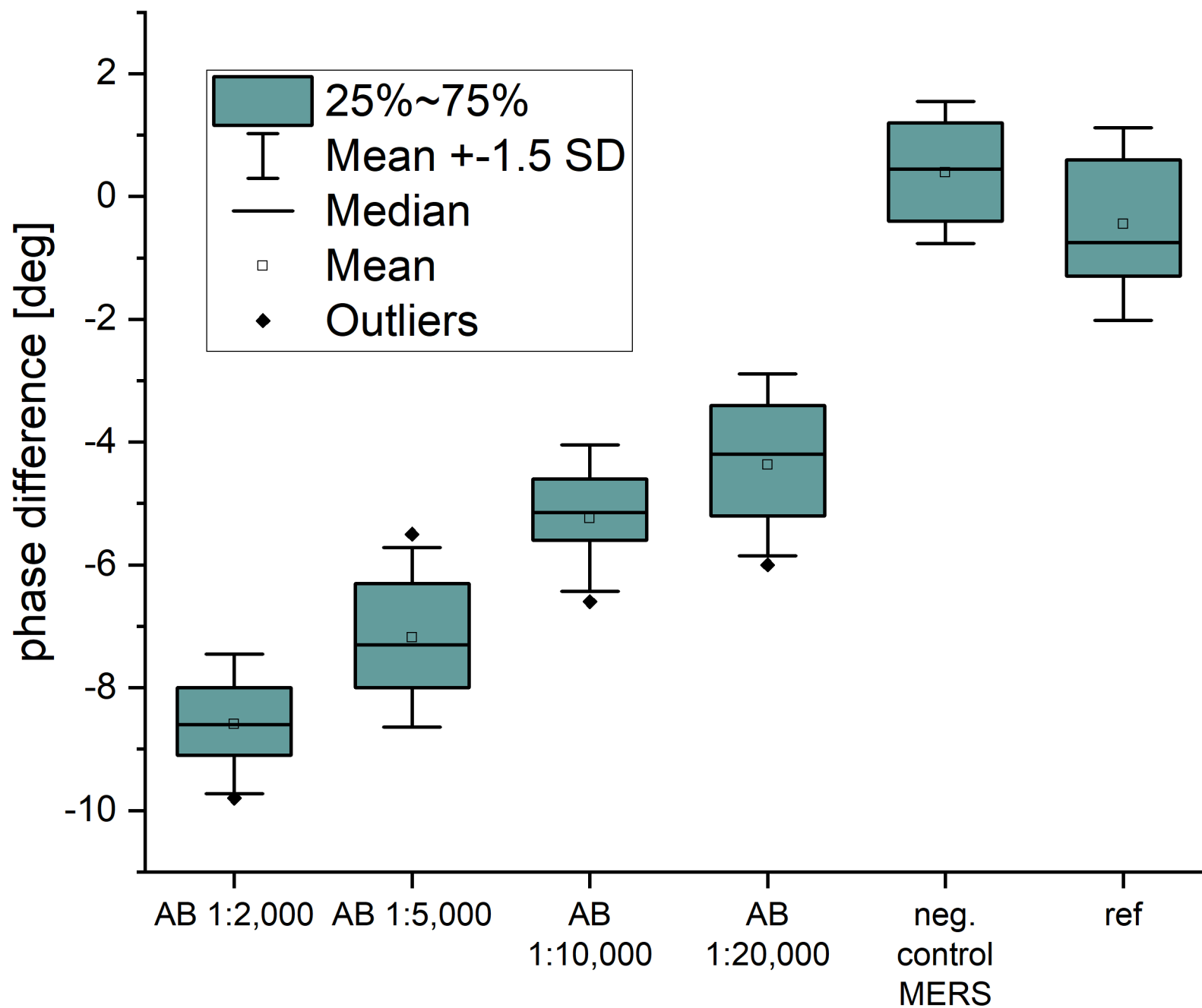

Supplement: Supplementary file 7 — Source Data [file 41467_2022_34941_MOESM7_ESM.zip › SI_fig16/SI_fig16_2.pdf]

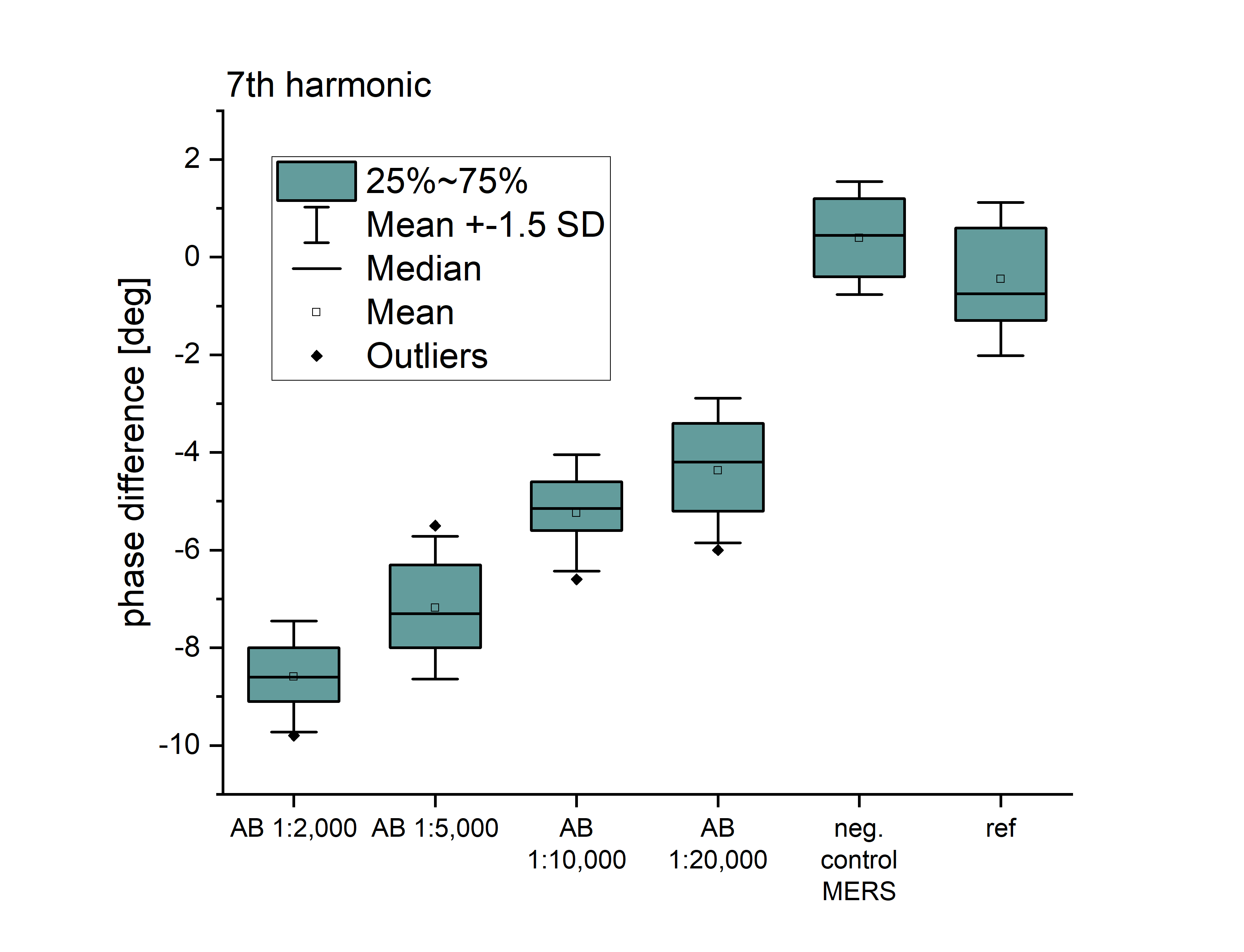

Supplement: Supplementary file 7 — Source Data [file 41467_2022_34941_MOESM7_ESM.zip › SI_fig16/SI_fig16_2.png]

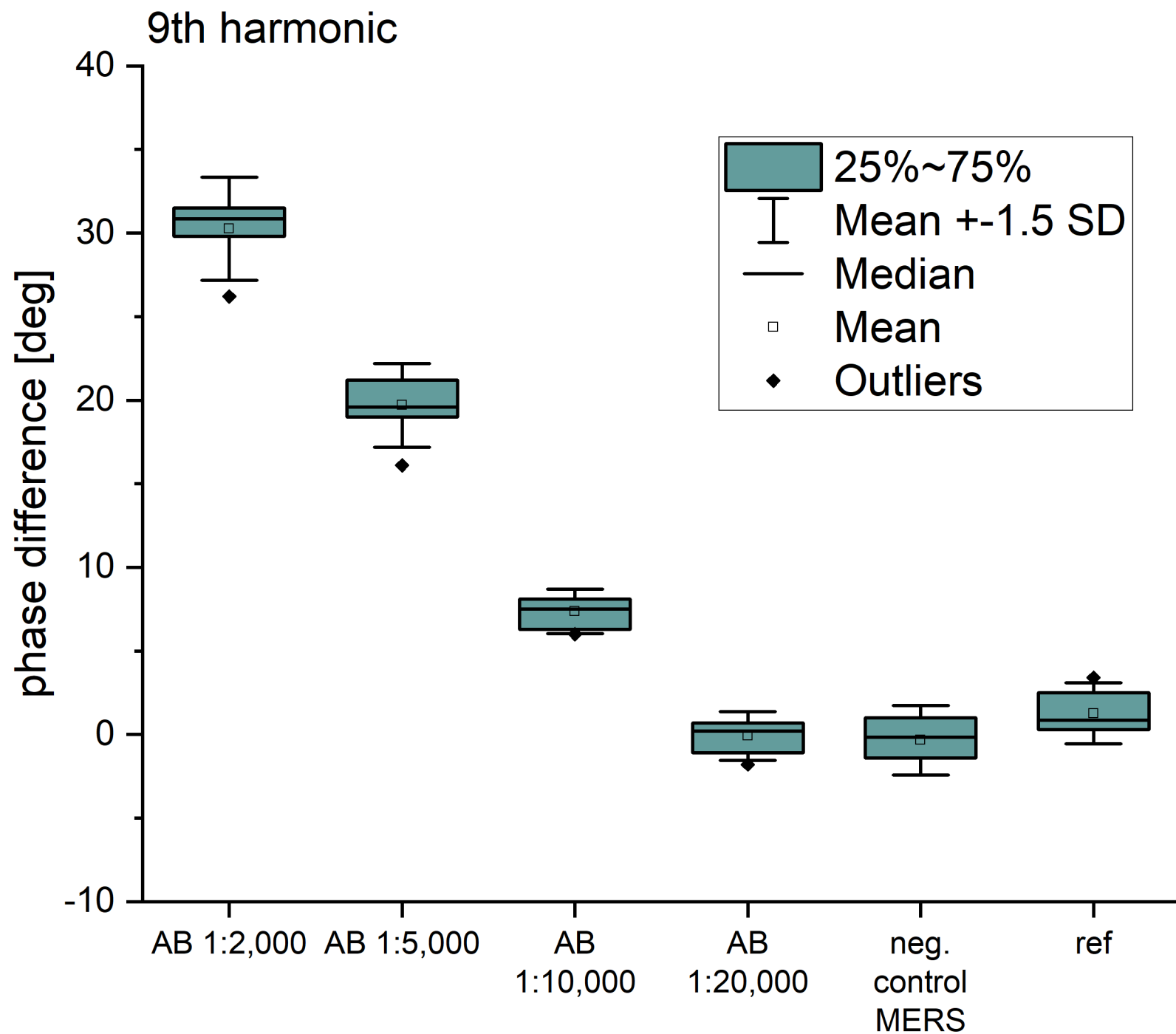

Supplement: Supplementary file 7 — Source Data [file 41467_2022_34941_MOESM7_ESM.zip › SI_fig16/SI_fig16_3.pdf]

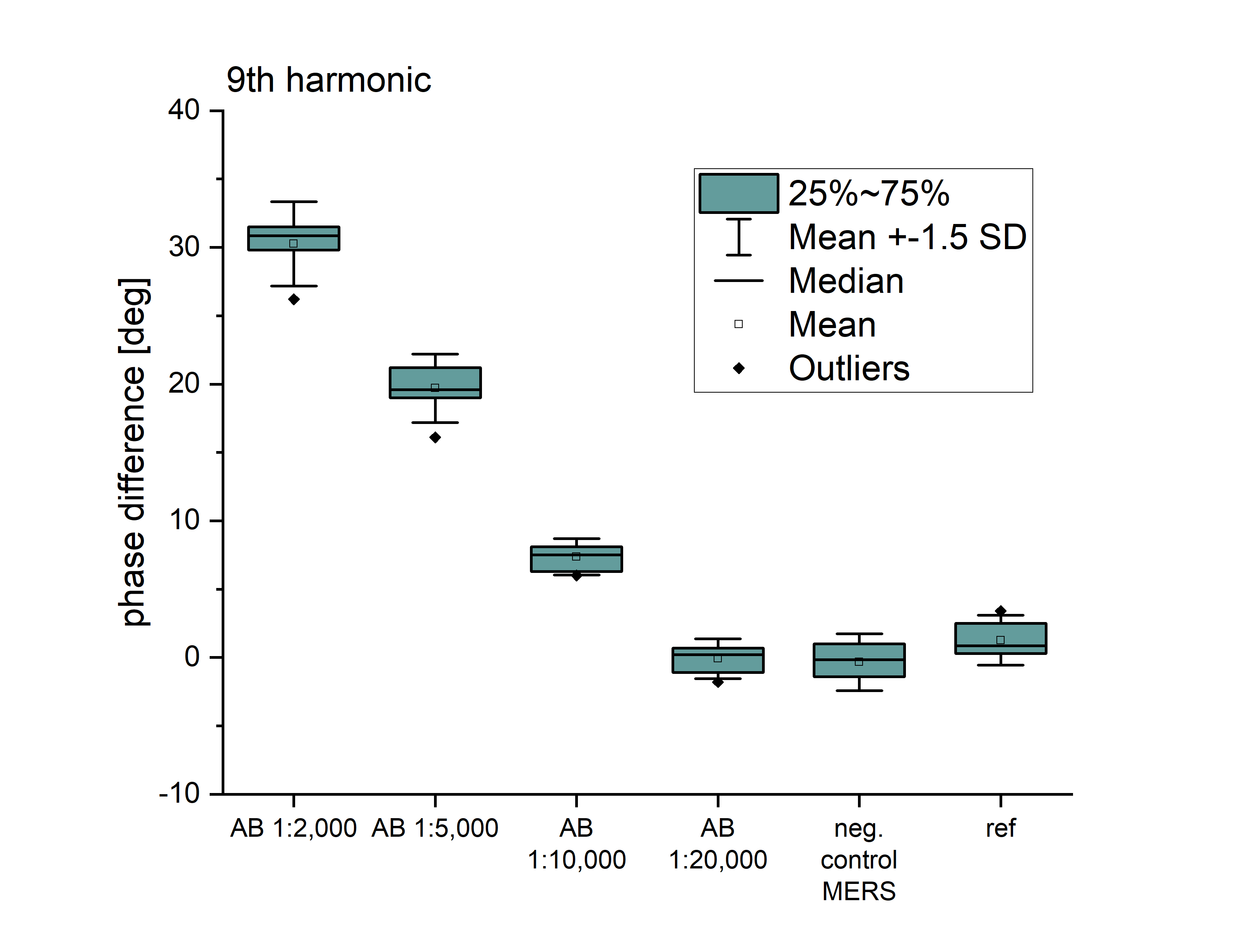

Supplement: Supplementary file 7 — Source Data [file 41467_2022_34941_MOESM7_ESM.zip › SI_fig16/SI_fig16_3.png]

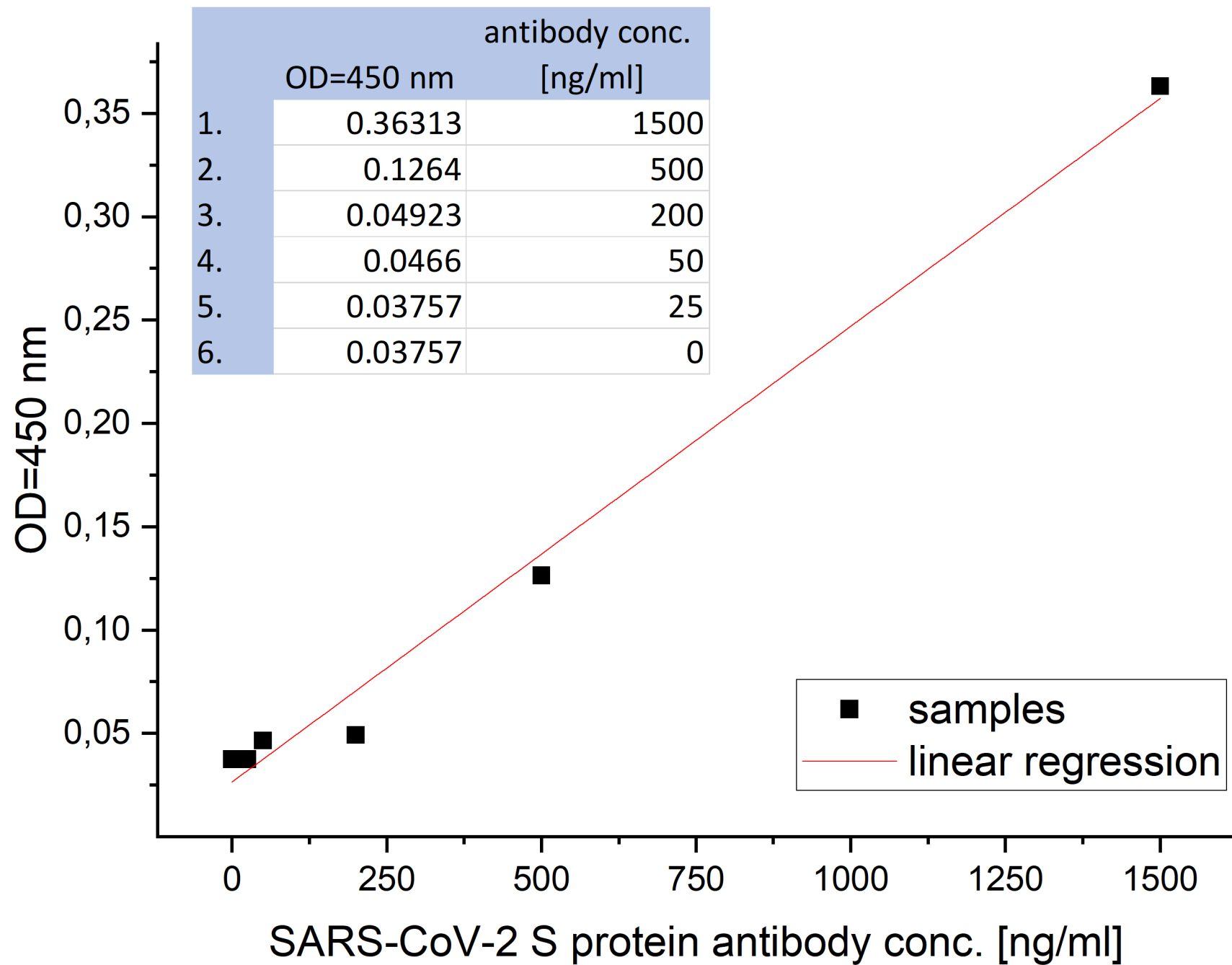

Supplement: Supplementary file 7 — Source Data [file 41467_2022_34941_MOESM7_ESM.zip › SI_fig17/SI_fig17.pdf]

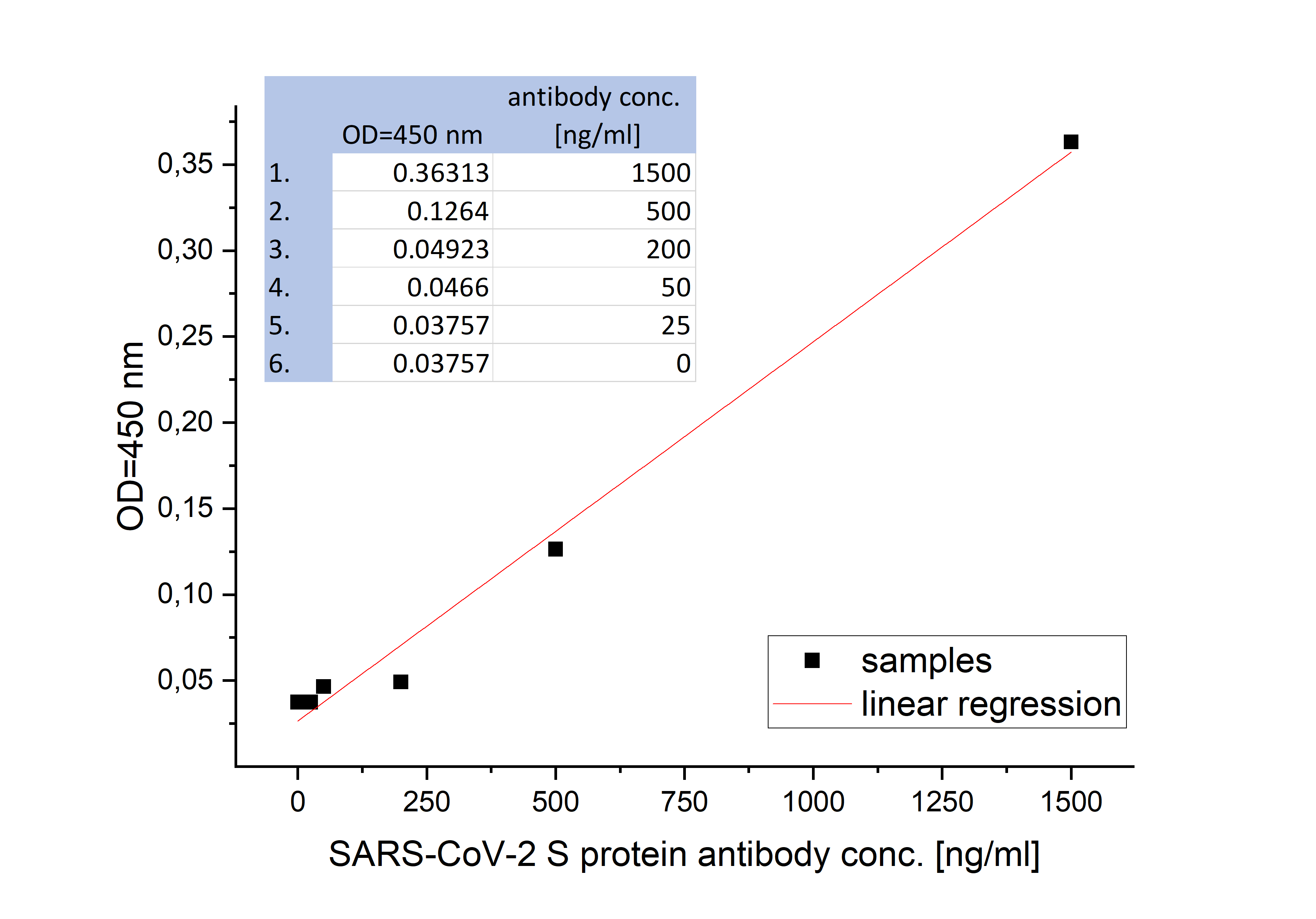

Supplement: Supplementary file 7 — Source Data [file 41467_2022_34941_MOESM7_ESM.zip › SI_fig17/SI_fig17.png]

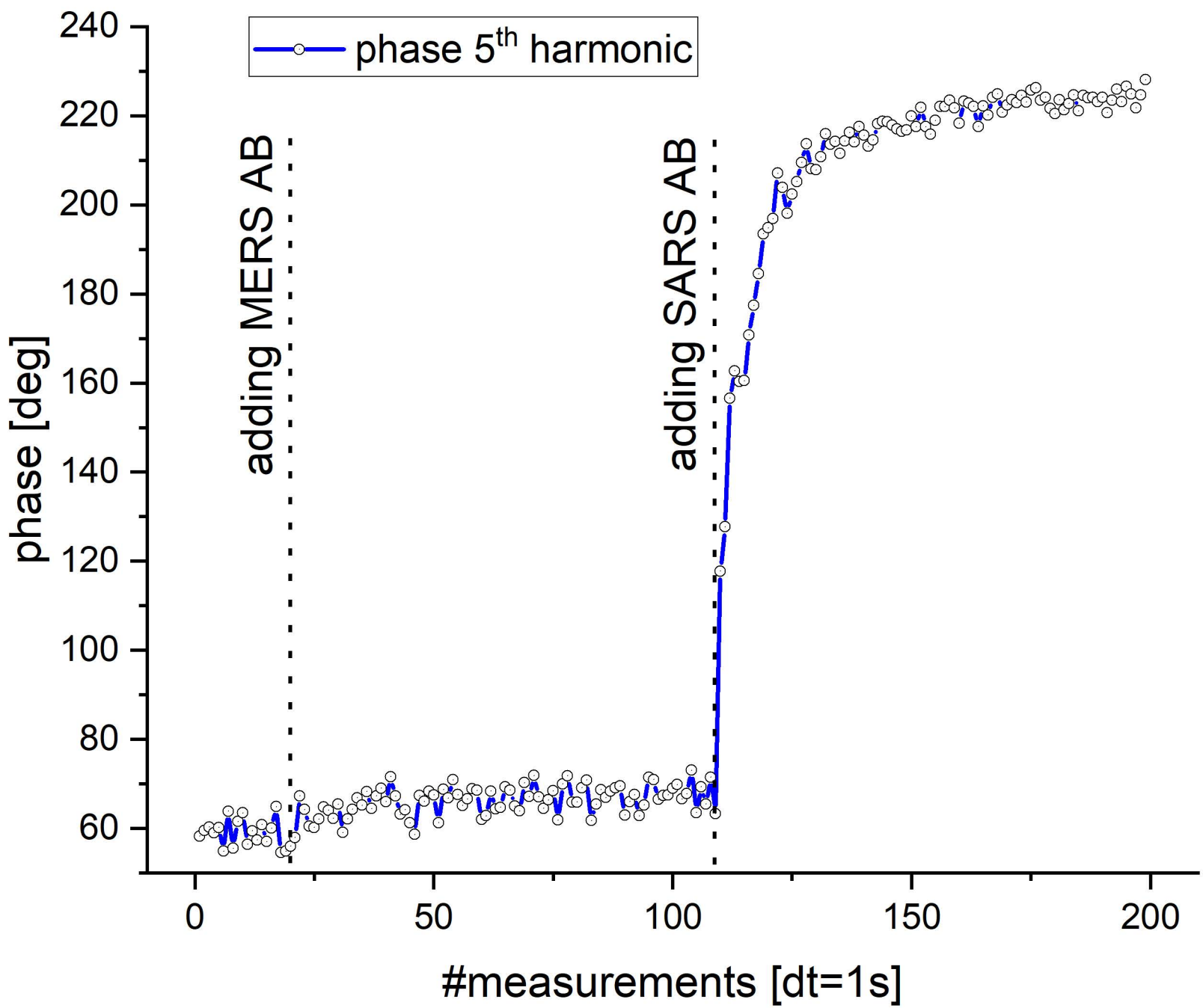

Supplement: Supplementary file 7 — Source Data [file 41467_2022_34941_MOESM7_ESM.zip › SI_fig18/SI_fig18.pdf]

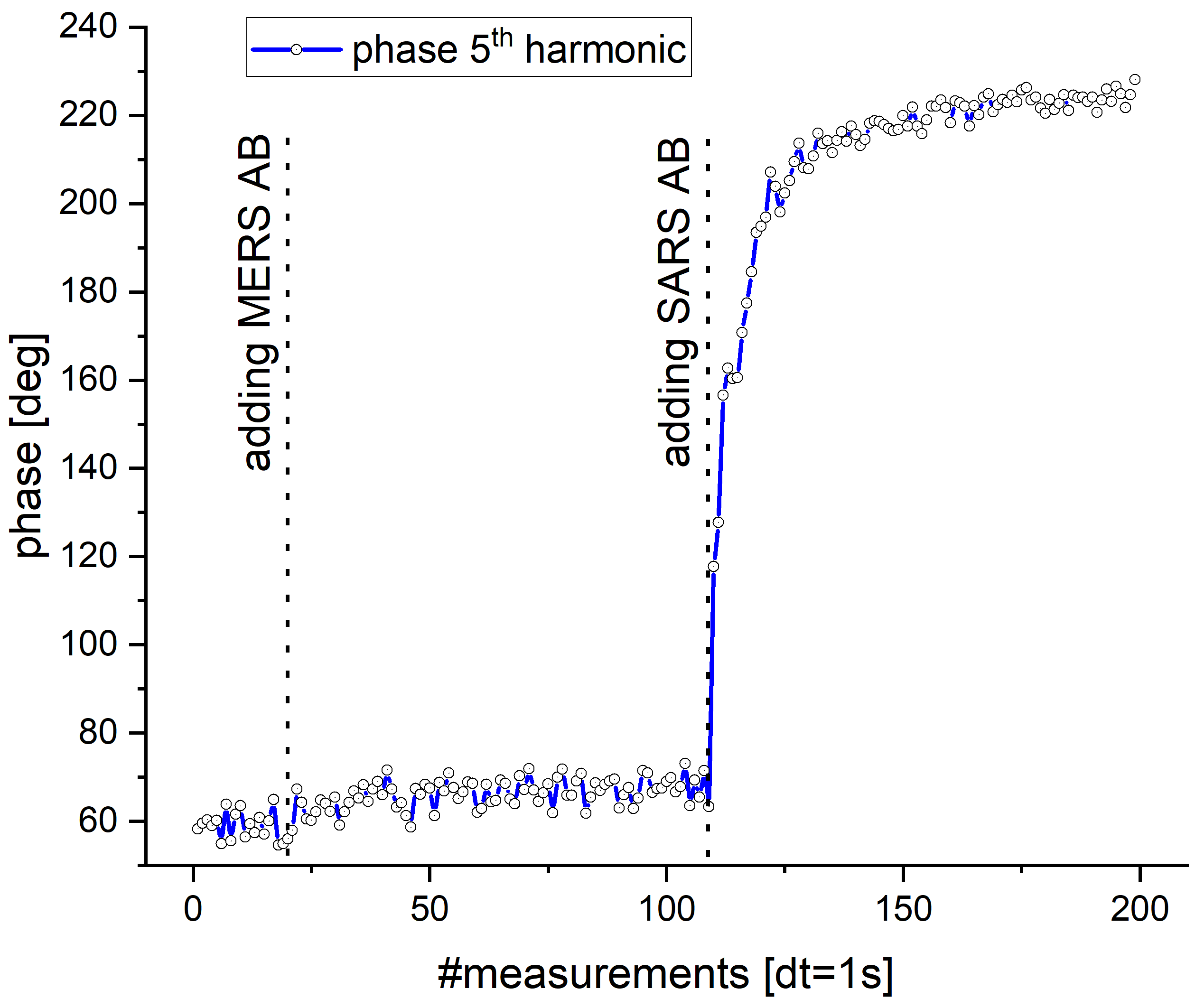

Supplement: Supplementary file 7 — Source Data [file 41467_2022_34941_MOESM7_ESM.zip › SI_fig18/SI_fig18.png]
